# Zinc finger protein ZNF384 is an adaptor of Ku to DNA during classical non-homologous end-joining

**DOI:** 10.1038/s41467-021-26691-0

**Published:** 2021-11-12

**Authors:** Jenny Kaur Singh, Rebecca Smith, Magdalena B. Rother, Anton J. L. de Groot, Wouter W. Wiegant, Kees Vreeken, Ostiane D’Augustin, Robbert Q. Kim, Haibin Qian, Przemek M. Krawczyk, Román González-Prieto, Alfred C. O. Vertegaal, Meindert Lamers, Sébastien Huet, Haico van Attikum

**Affiliations:** 1 Department of Human Genetics, Leiden University Medical Center, Leiden, The Netherlands; 2 Univ Rennes, CNRS, IGDR (Institut de génétique et développement de Rennes)—UMR 6290, BIOSIT–UMS3480, F-35000 Rennes, France; 3 Institut de Biologie François Jacob, Institute of Cellular and Molecular Radiobiology, Université Paris-Saclay, Université de Paris, CEA, F-92265 Fontenay-aux-Roses, France; 4 Department of Cell and Chemical Biology, Leiden University Medical Center, Leiden, The Netherlands; 5 Department of Medical Biology, Amsterdam University Medical Centers (location AMC), Cancer Center Amsterdam, Amsterdam, The Netherlands; 6 Institut Universitaire de France, F-75000 Paris, France

**Keywords:** DNA-binding proteins, Non-homologous-end joining

## Abstract

DNA double-strand breaks (DSBs) are among the most deleterious types of DNA damage as they can lead to mutations and chromosomal rearrangements, which underlie cancer development. Classical non-homologous end-joining (cNHEJ) is the dominant pathway for DSB repair in human cells, involving the DNA-binding proteins XRCC6 (Ku70) and XRCC5 (Ku80). Other DNA-binding proteins such as Zinc Finger (ZnF) domain-containing proteins have also been implicated in DNA repair, but their role in cNHEJ remained elusive. Here we show that ZNF384, a member of the C2H2 family of ZnF proteins, binds DNA ends in vitro and is recruited to DSBs in vivo. ZNF384 recruitment requires the poly(ADP-ribosyl) polymerase 1 (PARP1)-dependent expansion of damaged chromatin, followed by binding of its C2H2 motifs to the exposed DNA. Moreover, ZNF384 interacts with Ku70/Ku80 via its N-terminus, thereby promoting Ku70/Ku80 assembly and the accrual of downstream cNHEJ factors, including APLF and XRCC4/LIG4, for efficient repair at DSBs. Altogether, our data suggest that ZNF384 acts as a ‘Ku-adaptor’ that binds damaged DNA and Ku70/Ku80 to facilitate the build-up of a cNHEJ repairosome, highlighting a role for ZNF384 in DSB repair and genome maintenance.

## Abstract (web summary)

Classical non-homologous end-joining (cNHEJ) is the dominant pathway used by human cells to repair DNA double-strand breaks (DSBs) and maintain genome stability. Here the authors show that PARP1-driven chromatin expansion allows the recruitment of ZNF384, which in turn recruits Ku70/Ku80 to facilitate cNHEJ.

## Introduction

DNA double-strand breaks (DSBs) represent one of the most toxic lesions that can occur in the human genome. If left unrepaired or repaired incorrectly, they can lead to loss of genetic information, thereby contributing to the development of diseases, including cancer^1^. In order to maintain genomic stability, cells have evolved pathways for the signaling and repair of these DSBs^1^. DSB repair can occur by either homologous recombination (HR) or non-homologous end-joining (NHEJ). HR is the more faithful repair pathway, which is active in the S and G2 phases of the cell cycle. It requires end resection to form large stretches of single-stranded DNA (ssDNA), which in turn become coated by the ssDNA-binding complex RPA and the recombinase RAD51. Collectively, these and several other auxiliary factors contribute to HR by using the sister chromatid as a template for repair^2^. In contrast, the dominant repair pathway in human cells is canonical non-homologous end-joining (cNHEJ), which requires minimal DNA-end processing and is initiated by the binding of Ku70/Ku80 heterodimers to the broken ends, followed by activation of DNA-PKcs kinase and recruitment of APLF via its conserved Ku-binding motif (KBM). This facilitates the assembly of the XLF–XRCC4–LIG4 complex, which stimulated by PAXX, ligates the broken ends predominantly in an error-free manner^3^. When cNHEJ is disabled, DSB repair can also occur via alternative non-homologous end-joining (aNHEJ), which seals the broken ends in an error-prone fashion by microhomology usage and in a manner dependent on the XRCC1-Ligase III complex or POLQ^3^. Alternatively, in the case of more extensive end-resection, microhomology usage may lead to deleterious, RAD52-dependent repair of DSBs via single-strand annealing (SSA)^4^.

Efficient detection and repair of DSBs is complicated by the packaging of DNA into chromatin. ATP-dependent chromatin remodeling enzymes and a wide plethora of enzymes that induce post-translational modifications (PTMs) on damaged chromatin, including but not limited to acetylation, methylation, and ubiquitylation, are therefore required to change chromatin structure at DSB sites to facilitate repair^5–7^. One of these enzymes is poly(ADP-ribosyl) polymerase 1 (PARP1), which becomes activated upon binding to DNA breaks and promotes chromatin expansion by the formation of poly(ADP-ribose) (PAR) chains on itself and adjacent nuclear proteins, such as histones, as well by facilitating the recruitment of ATP-dependent chromatin remodelers in the vicinity of these breaks^8–10^. This increases chromatin accessibility and the recruitment of several DSB repair proteins, including Ku70/Ku80 and XRCC4, via direct PAR binding or DNA binding^9,11^.

Interestingly, a number of transcription factors have also been shown to localize at sites of DNA damage either in a PARP/PAR-dependent manner or via their DNA-binding domains^11,12^. Their role in DSB repair, is, however, largely unknown. One such class of transcription factors are Zinc Finger (ZnF) domain-containing proteins. ZnF domains exist in ~5% of all human proteins and bind to a large variety of substrates, including DNA, RNA, lipids, and post-translational modifications (PTMs)^13,14^. Due to their versatile binding ability, ZnF proteins play roles in different cellular processes, such as transcription regulation, signal transduction, and cell migration^15^. Interestingly, recent studies have implicated ZnF domain-containing proteins as new players in DSB repair^13^. For instance, ZMYND8 was found to play a role in transcription repression during DSB repair via HR^16^, whereas ZNF830 promotes HR by facilitating RBBP8 (CTIP)-dependent DNA-end resection^17^. ZNF281, on the other hand, was shown to promote XRCC4-dependent NHEJ of DSBs^18^. Together, these findings suggest a more important role for ZnF domain proteins in DSB repair than previously anticipated, although their mode of action is still poorly understood.

Here, we describe an important regulatory role for the C2H2-type ZnF protein ZNF384 in DSB repair by cNHEJ. ZNF384 is recruited to sites of DNA damage in a manner dependent on PARP1/PAR-mediated chromatin expansion followed by binding to the exposed DNA via its internal C2H2 domain. Moreover, ZNF384 physically interacts with Ku70/Ku80 via its N-terminus and both its interaction with DNA and Ku70/Ku80 are critical for efficient Ku70/Ku80 loading. This in turn allows for the assembly of a complete repairosome that includes cNHEJ proteins such as APLF and XRCC4/LIG4, thereby facilitating cNHEJ. Collectively, our data show that zinc-finger protein ZNF384 is an adapter of Ku to DNA during DSB repair via cNHEJ.

## Results

### ZNF384 is recruited to DNA damage sites and interacts with NHEJ proteins

**Fig. 1:**
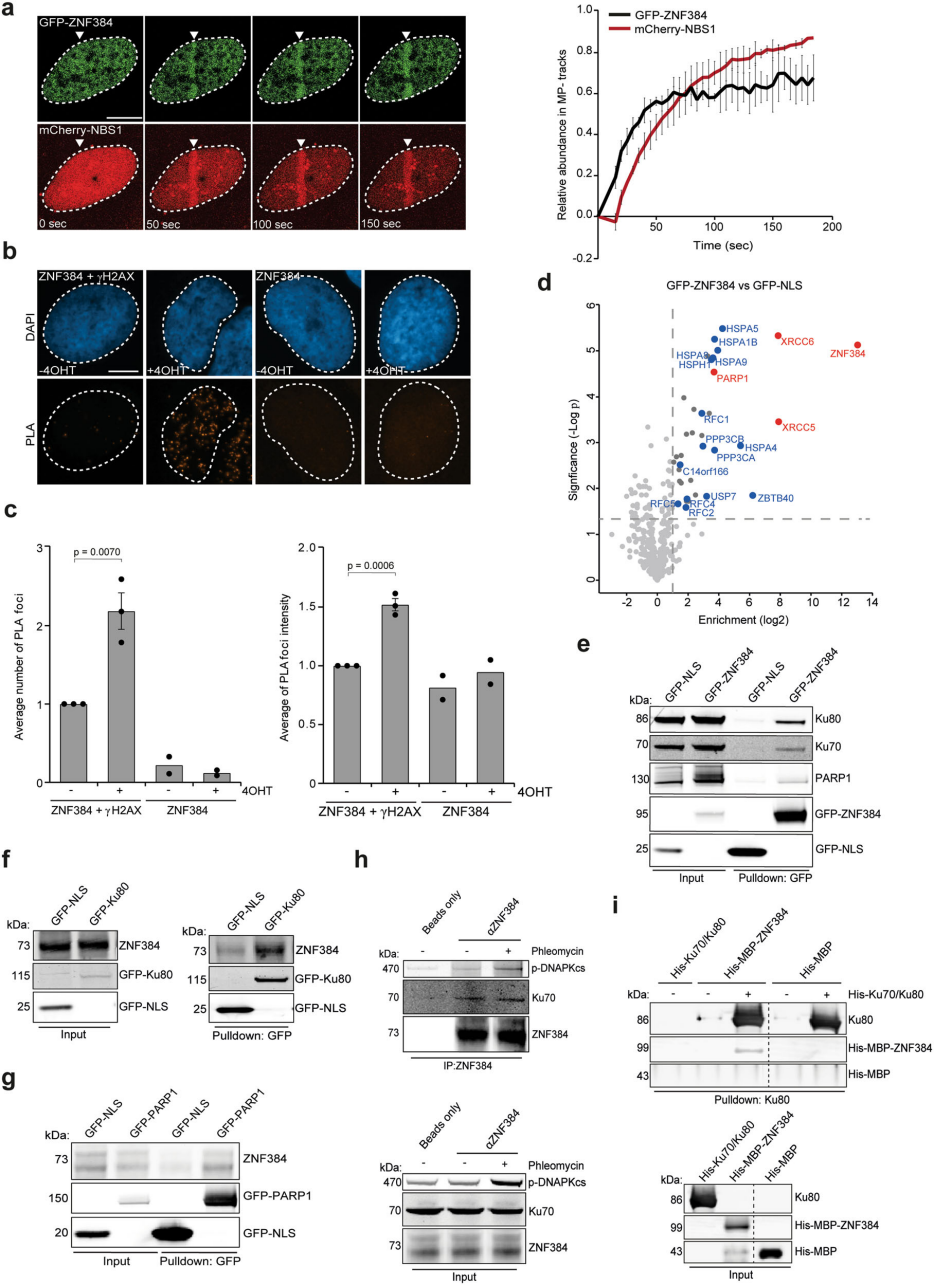
ZNF384 is recruited to DNA damage sites and interacts with NHEJ proteins. **a** Recruitment of GFP-ZNF384 to 800 nm multiphoton tracks in U2OS Flp-In/T-Rex *ZNF384* KO cells. mCherry-NBS1 was used as a DNA damage marker (left panel). White triangles indicate irradiated regions. Quantification of the data is plotted on a timescale as relative abundance in tracks. Peak values were set to 1. The graph represents the mean ± SD of >30 cells acquired in 2–3 independent experiments (right panel). **b** PLA of ZNF384 and γH2AX in AsiSI-ER-U2OS cells treated with 4-OHT for DSB induction. PLA foci were quantified after 5 h of DSB induction. **c** Quantification of (**b**). The mean ± SEM of PLA foci formation and foci intensity from >200 cells acquired in 2–3 independent experiments are shown. Statistical significance was calculated using the two-tailed Student’s *t* test. **d** Volcano plot depicting the statistical differences of the MS analysis on GFP-ZNF384 versus GFP-NLS pull-downs. The enrichment is plotted on the *x* axis and the significance (*t* test −log2 *P* value) is plotted on the *y* axis. NHEJ factors are shown in red and several hits are shown in blue (see also Supplementary Table 1). **e** Pull-down of the indicated GFP fusion proteins in U2OS Flp-In/T-Rex cells. Blots were probed for GFP, Ku70, Ku80, and PARP1. **f** Pull-downs of the indicated GFP fusion proteins in Hela cells. Blots were probed for GFP and ZNF384. **g** Pull-downs of the indicated GFP fusion proteins in Hela cells. Blots were probed for GFP and ZNF384. **h** Immunoprecipitation (IP) of endogenous ZNF384 from 500 µM Phleomycin-treated U2OS cells. Control IP contained beads only. Blots were probed for ZNF384, p-DNA-PKcs (S2056), and Ku70. **i** In vitro Ku80 pull-down in the presence or absence of His-Ku70/Ku80 and His-MBP or His-MBP-ZNF384. Control IP contained beads only. Blots were probed for Ku80 and MBP. Scale bar 5 μm. Source data are provided as a Source Data file.

ZNF384 was among the candidate ZnF proteins that localize at sites of DNA damage induced by laser micro-irradiation^12^. To validate this finding, we transiently co-expressed GFP-tagged ZNF384 (isoform 2, containing 6 C2H2 motifs) and the DNA damage sensor mCherry-NBS1 in *ZNF384* knockout (KO) U2OS cells (Supplementary Fig. 1A) and measured their recruitment to sites of DNA damage induced by multiphoton irradiation using live-cell imaging (Supplementary Fig. 1B). GFP-ZNF384 was recruited to NBS1-marked DNA damage sites within 1 min and remained enriched at these sites for at least 3 minutes (Fig. 1a). We also observed the accumulation of endogenous ZNF384 at UV-A laser-induced DNA damage, which was completely abolished following ZNF384 knockdown, confirming the specificity of the ZNF384 antibody (Supplementary Fig. 1C). Since multiphoton and UV-A laser micro-irradiation may induce a variety of lesions other than DSBs, we next examined whether ZNF384 is specifically recruited to DSBs. First, we monitored its accumulation at chromatin regions micro-irradiated by ultrasoft X-rays (USX)^19^. Endogenous ZNF384 accumulated at USX-induced DSBs, co-localizing with γH2AX (Supplementary Fig. 1D, E), and with the core cNHEJ proteins Ku70 and XRCC4 (Supplementary Fig. 1F, G). Second, we measured the colocalization between ZNF384 and γH2AX at *Asi*SI nuclease-induced DSBs the proximity ligation assay (PLA) (Fig. 1b). We observed a clear colocalization between ZNF384 and γH2AX at these DSBs (Fig. 1c), as well as between TP53BP1 (53BP1) and γH2AX (Supplementary Fig. 1H, I and ref. ^20^), showing the validity of the approach. Together, these observations demonstrate that ZNF384 is recruited to DSBs.

To gain insight into ZNF384’s function at sites of DNA damage, we aimed to identify possible interaction partners of ZNF384. To this end, we generated U2OS Flp-In/T-Rex cells stably expressing inducible GFP-tagged ZNF384 or GFP-NLS, and performed GFP-trap-based pull-downs followed by label-free mass spectrometry (MS) (Fig. 1d). Our analysis revealed that ZNF384 interacts with 24 proteins that were at least twofold enriched in GFP-ZNF384 pull-downs when compared to those of GFP-NLS (Fig. 1d and Supplementary Table 1). Interestingly, Ku70/Ku80 and PARP1 belonged to the top interactors, all of which regulate DSB repair via cNHEJ^9,21^. GFP pull-downs coupled to western blot analysis confirmed that GFP-tagged ZNF384 interacts with endogenous Ku70, Ku80, and PARP1 (Fig. 1e), while reciprocal pull-downs of both GFP-Ku80 (Fig. 1f) and GFP-PARP1 (Fig. 1g) revealed interactions with endogenous ZNF384. Moreover, we also confirmed the ZNF384-Ku70 interaction endogenously (Fig. 1h), using *ZNF384* knockdown cells to show the specificity of the ZNF384 signal in the Ku70 immunoprecipitation (Supplementary Fig. 1J). To rule out an indirect interaction between these proteins, we purified His-MBP-tagged ZNF384 and addressed its ability to bind Ku70/Ku80 in vitro. In agreement with our in vivo pull-down results, we found that recombinant ZNF384 bound recombinant Ku70/Ku80, demonstrating a direct protein–protein interaction (Fig. 1i). These findings suggest that ZNF384 forms a complex with Ku70/Ku80 and PARP1, manifesting a possible role for ZNF384 in NHEJ.

### ZNF384 recruitment to DNA damage sites requires the activity of PARP1

Because of ZNF384’s interaction with Ku70/Ku80 and PARP1, we first analyzed whether it is recruited to DNA breaks via Ku80. We observed that GFP-ZNF384 recruitment to UV-A laser micro-irradiation induced DNA damage, as well as that of the DNA damage marker NBS1 (Supplementary Fig. 2A, B), remained unchanged in cells depleted of Ku80 or its interaction partner in the DNA-PK complex, DNA-PKcs kinase (Supplementary Fig. 2A, B)^22^. Moreover, we also found endogenous ZNF384 at γH2AX-marked UV-A laser-inflicted DNA damage to remain unaffected in cells depleted of Ku70 (Supplementary Fig. 2C, D), Ku80, or DNA-PKcs (Supplementary Fig. 2E), which was confirmed in *Xrs-5* KO hamster cells defective for Ku80 (Supplementary Fig. 2F, G) and ref. ^23^, ruling out effects of incomplete knockdown. In addition, inhibition of DNA-PKcs kinase activity did not exert any effect on the recruitment of GFP-ZNF384 (Supplementary Fig. 2H) and endogenous ZNF384 (Supplementary Fig. 2I, J), suggesting that ZNF384 recruitment is independent of DNA-PK.

**Fig. 2:**
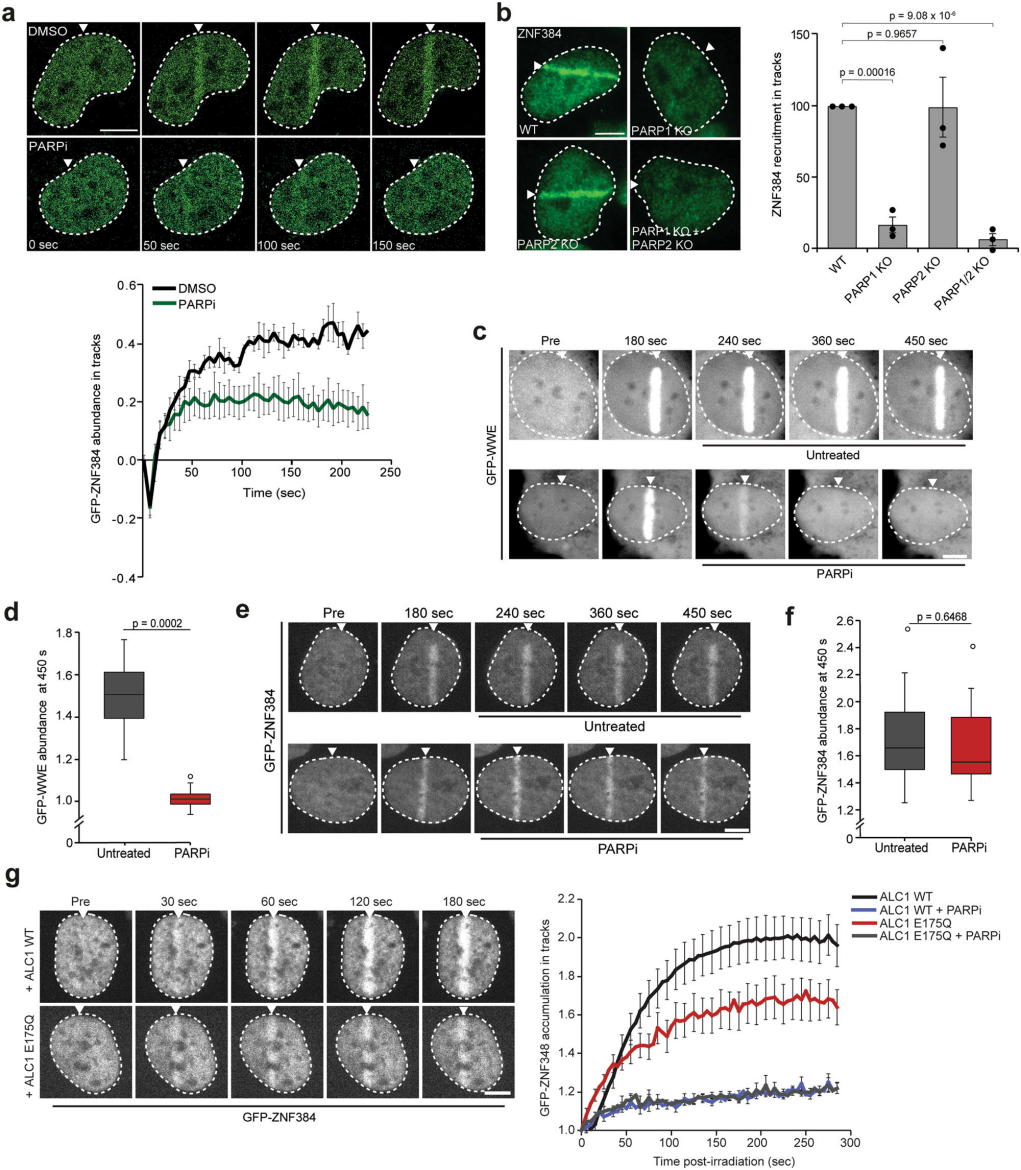
PARP1 activity facilitates DNA binding of ZNF384 at sites of damage. **a** GFP-ZNF384 recruitment to 800 nm multiphoton tracks in stable U2OS Flp-In/T-Rex cells treated with PARP inhibitor (PARPi) for 1 h before micro-irradiation (top panel). Quantification of the data is presented as the mean ± SEM of >35 cells acquired in three independent experiments (bottom panel). **b** ZNF384 recruitment to 365 nm UV-A tracks 10 min after DNA damage induction in BrdU-sensitized wild-type (WT) and the indicated KO U2OS cells (left panel). The mean ± SEM of >180 cells from three independent experiments is shown (right panel). **c** Confocal images showing accumulation of GFP-WWE at sites of 405 nm laser micro-irradiation in Hoechst-sensitized U2OS cells. Cells were left untreated or treated with PARPi 180 s after DNA damage induction. **d** Boxplot limits correspond to the 25th and 75th percentiles and the center line in the box indicates the median value of the accumulation of GFP-WWE at 450 s post irradiation from 23–25 cells from a representative of three independent experiments. **e** As in (**c**), except for GFP-ZNF384. **f** As in (**d**), except for GFP-ZNF384 from 21–27 cells. Boxplot limits correspond to the 25th and 75th percentiles and the center line in the box indicates the median value. The whiskers extend 1.5 times the interquartile range. **g** GFP-ZNF384 recruitment to 405 nm laser tracks in U2OS cells overexpressing iRFP-ALC1 wild-type (WT) and iRFP-ALC1 ATPase-dead (E175Q) treated with PARPi for 1 h before micro-irradiation (left panel). GFP-ZNF384 recruitment is displayed as intensity integrated over the damaged area (right panel). The mean ± SEM from 13–16 cells from a representative of three independent experiments is shown. White triangles indicate irradiated regions. Scale bar 5 μm. All *P* values were calculated using the unpaired Student’s *t* test, assuming unequal variances. Source data are provided as a Source Data file.

Next, we treated cells with the PARP1 and PARP2 inhibitor (PARPi) olaparib (Fig. 2a) and found this treatment to impair the recruitment of GFP-ZNF384 at multiphoton laser micro-irradiation (Fig. 2a), as well as UV-A laser micro-irradiation induced DNA damage (Supplementary Fig. 3A, B). Similarly, knockout of PARP1 alone or in combination with PARP2 completely impaired ZNF384 recruitment, whereas knockout of PARP2 alone, and knockdown of PARP3 had no major effect (Fig. 2b and Supplementary Fig. 3C–F). PARP3 knockdown, however, impaired Ku80 recruitment as expected (Supplementary Fig. 3G, H and ref. ^24^). Importantly, we previously showed that PARP1 itself is still recruited to sites of DNA damage in PARPi-treated cells^25^, suggesting that the recruitment of ZNF384 does not involve a physical interaction between ZNF384 and PARP1, but rather relies on PARP1’s activity. Possibly, this interaction is important for the PARP1-dependent PARylation of ZNF384, which we and others observed in response to DSB induction (Supplementary Fig. 3I and ref. ^26^. Collectively, these results suggest that ZNF384 is rapidly recruited to DSB-containing tracks in a manner dependent on the activity of PARP1.

### PARP1-dependent chromatin unfolding facilitates DNA binding of ZNF384 at DNA damage sites

We next asked whether the PARP1 activity-dependent recruitment of ZNF384 to DNA breaks could be due to the direct binding of ZNF384 to PARP1-generated PAR moieties. To investigate this, we used a previously established fluorescence three-hybrid assay (Supplementary Fig. 4A)^27^. This assay measures the ability of a lacO-anchored putative “ADP-ribose-binding” protein of interest to interact with PARylated PARP1 that is naturally released from sites of laser-induced DNA damage and is then free to diffuse and bind to the lacO-anchored PAR-binder. Indeed, we observed recruitment of PARylated PARP1 to the lacO-anchored macrodomain of macroH2A1.1, a well-characterized PAR-binding protein^11,28^, which was abolished following treatment with PARPi, but remained unaffected by treatment with PARGi (likely due to the availability of a limited number of lacO-anchored macrodomain molecules) (Supplementary Fig. 4B–D). In contrast, we did not observe an interaction between lacO-anchored ZNF384 and PARylated PARP1 (Supplementary Fig. 4E, F). To corroborate and extend these findings, we used a second independent approach that can discriminate whether ZNF384 is recruited by binding to PAR, or to DNA that becomes exposed upon PAR-dependent chromatin relaxation. In this assay *ZNF384* KO cells were micro-irradiated and, 240 seconds post irradiation, after the completion of the initial wave of PARP/PAR-dependent chromatin relaxation^9,27,29^, PARPi was added to acutely block PARP enzymatic activity. Under these conditions, PAR-binding proteins are rapidly released from the damaged area, while proteins that bind DNA are maintained^11^. Indeed, we observed that the WWE domain of RNF146, which is a known PAR-binder^30^, was rapidly released from the irradiated area (Fig. 2c, d and Supplementary Fig. 5A). While its binding was nearly completely reversed at 450 seconds post irradiation due to rapid degradation of PAR (Supplementary Fig. 5A), counteracting the removal of PAR chains by adding PARGi to the PARPi-treated cells preserved the accumulation of the WWE domain (Supplementary Fig. 5A, B). In contrast, PARPi treatment did not revert the recruitment of GFP-ZNF384 at the damaged area (Fig. 2e, f), suggesting that ZNF384 does not bind PAR, but rather associates with DNA. To corroborate these findings, we investigated whether DNA binding of ZNF384 depends on PARP/PAR-dependent chromatin relaxation, which we and others have shown to facilitate the association of DNA-binding proteins with DNA at sites of damage^9,27,29^. We found that overexpression of the ATP-dependent chromatin remodeler ALC1, which enhances chromatin unfolding without affecting PAR-signaling (Supplementary Fig. 5C and ref. ^27^), increased ZNF384 accumulation at DNA damage sites when compared to that after overexpression of ATPase-dead (E175Q) ALC1 (Fig. 2g). PARPi treatment inhibited ZNF384 recruitment in both WT ALC1 and ATPase-dead (E175Q) ALC1 overexpressing cells (Fig. 2g), consistent with the PARP-dependent recruitment of these proteins^8^. Interestingly, we did not find a significant enrichment of ZNF384 in chromatin-enriched extracts from cells treated with phleomycin (Supplementary Fig. 5D, E), or a change in ZNF384 turnover at DNA lesions as measured by FRAP (Supplementary Fig. 5F–H), suggesting that the binding of ZNF384 to damaged DNA is not qualitatively different from its binding to undamaged DNA and is mostly triggered by the increased accessibility of DNA consecutive to PAR-driven chromatin relaxation. This behavior of ZNF384 is comparable to that of the DNA-binding domain BZIP of C/EBPa (Supplementary Fig. 5I and ref. ^11^) and the chromatin remodeler CHD4 (Supplementary Fig. 5I and ref. ^27^), which were both shown to recruit to DNA lesions due to increased accessibility of damaged DNA through PAR-dependent chromatin unfolding. In contrast, the ZNF384 interaction partners and DNA-end binding proteins Ku70/Ku80 showed a clear slowing of its turnover following DNA damage (Supplementary Fig. 5J). Thus, ZNF384 recruitment is dependent on PAR-dependent chromatin unfolding, allowing ZNF384 to bind to the exposed damaged DNA.

**Fig. 3:**
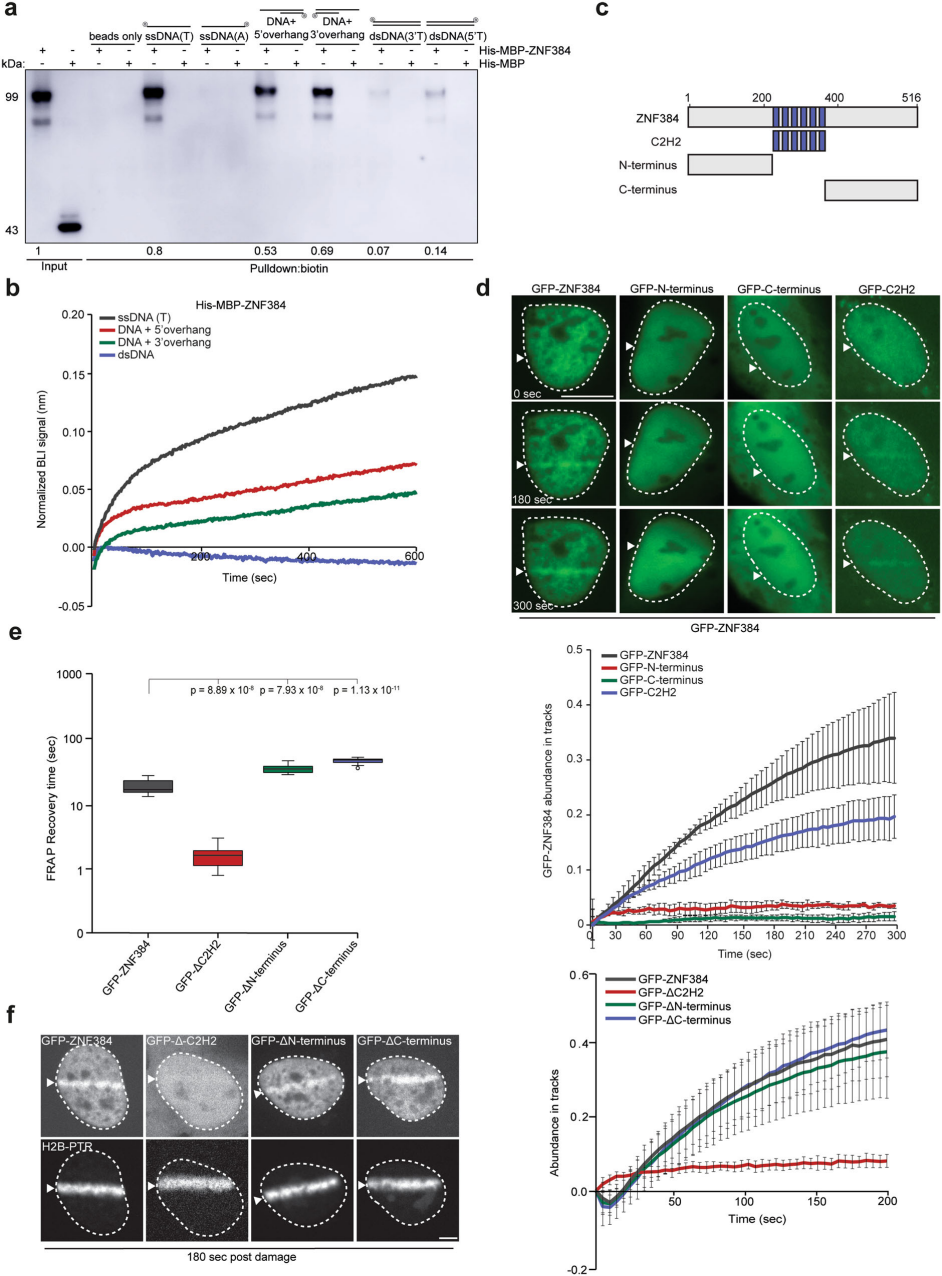
ZNF384 is recruited to sites of DNA damage via its C2H2 DNA-binding motif. **a** DNA pull-downs of the indicated biotinylated DNA substrates in the presence of His-MBP-C2H2 or His-MBP (control). Blots were probed for MBP. His-MBP-ZNF384 pull-down signals were normalized to the Input signal, which was set to 1. The mean from four independent experiments is indicated below the blot. His-MBP was not detectable in pull-downs. A representative experiment is shown. **b** DNA binding of His-MBP-ZNF384 to the indicated biotinylated DNA substrated as measured by BLI. Quantified data are plotted on a timescale and normalized to His-MBP (control). **c** Schematic representation of full-length ZNF384 protein and its domains (C2H2, N-terminus, and C-terminus). **d** Live-cell imaging of the recruitment of the indicated GFP-ZNF384 proteins to DNA damage tracks generated by 365 nm UV-A laser micro-irradiationin BrdU-sensitized *ZNF384* KO U2OS Flp-In/T-Rex cells. mCherry-NBS1, which was co-expressed with the GFP-ZNF384 proteins, served as a DNA damage marker. Representative images are shown. White triangles indicate irradiated regions. Scale bars: 10 µm (upper panel). Quantification of the data is shown as mean ± SEM from 30–40 cells (lower panel). **e** Quantification of FRAP measurements to assess the local dynamics of the indicated GFP-ZNF384 constructs. 12 cells per condition were analyzed. Boxplot limits correspond to the 25th and 75th percentiles and the center line in the box indicates the median value. The whiskers extend 1.5 times the interquartile range. *P* values were calculated using an unpaired Student’s *t* test, assuming unequal variances. **f** Live-cell imaging of the recruitment of the indicated GFP-ZNF384 proteins to DNA damage tracks generated by 405 nm laser micro-irradiation in Hoechst-sensitized *ZNF384* KO U2OS Flp-In/T-Rex cells. White triangles and photoactivatable H2B-PTR, which were co-expressed with the indicated GFP-ZNF384 proteins, indicate irradiated regions. Representative images are shown. Scale bars 4 µm (left panel). Data are presented as mean values ± SEM. *P* values were calculated using an unpaired Student’s *t* test, assuming unequal variances (right panel). Source data are provided as a Source Data file.

Previous work indeed showed that ZNF384 belongs to one of the few C2H2-type of ZnF proteins reported having unique DNA-binding affinity, particularly for homopolymeric dA·dT DNA consensus elements enriched in the genome^31^. We performed biolayer interferometry (BLI) experiments and in vitro DNA pull-down experiments using purified His-MBP-tagged ZNF384 and confirmed that His-MBP-ZNF384, in contrast to His-MBP alone, has a high affinity to bind T-rich ssDNA (Supplementary Fig. 6A, B), as opposed to A-rich ssDNA (Fig. 3a). Extending this finding, we also found ZNF384 to bind double-stranded (ds)DNA with either a 3′- or 5′ overhang in both BLI and in vitro DNA pull-down assays, albeit with a seemingly lower affinity when compared to ssDNA (Fig. 3a, b). Importantly, ZNF384 bound poorly, if at all, to dsDNA, and did not show RNA binding (Supplementary Fig. 6C). Moreover, it showed reduced colocalization with 5-ethynyl uridine (5-EU) labeled RNA compared to Hoechst labeled DNA as quantified by the Pearson correlation coefficient (Supplementary Fig. 6D). Collectively, these results suggest that ZNF384 recruitment to DNA damage sites is dependent on PARP-induced chromatin relaxation and its affinity to bind damaged DNA.

### ZNF384 is recruited to sites of DNA damage via its C2H2 DNA-binding motif

The fact that ZNF384 is recruited to DNA damage sites and binds DNA, encouraged us to investigate whether the C2H2, N-terminal or C-terminal domain of ZNF384 is implicated in this process (Fig. 3c). To this end, we purified His-MBP-tagged versions of these ZNF384 domains (Supplementary Fig. 6A), and assessed their ability to bind dsDNA with a 3′overhang, a common substrate found at DNA breaks, in vitro by BLI and DNA pull-down assay. As expected, full-length ZNF384 was able to bind this DNA substrate, whereas its N-terminus and C-terminus revealed very poor to no binding (Supplementary Fig. 7A, B). In contrast, the C2H2 domain showed stronger binding to this substrate, which was even comparable to that of full-length ZNF384 in the BLI assay (Supplementary Fig. 7A, B). Finally, we also observed that the C2H2 domain, similar to full-length ZNF384, has affinity for ssDNA, as well as for dsDNA with a 3′- or 5′-overhang, albeit that the affinity for the latter two substrates was seemingly lower when compared to that for ssDNA (Supplementary Fig. 7C, D). Together, these findings show that ZNF384 binds different DNA substrates through its C2H2 domain.

Given the different DNA-binding affinities of these ZNF384 domains, we next assessed their relevance for ZNF384 recruitment to DNA damage sites. To this end, we studied the recruitment of GPF-tagged versions of the C2H2, N-terminal or C-terminal domains to UV-A laser micro-irradiation inflicted DNA damage (Fig. 3d) in *ZNF384* KO U2OS cells. This was to avoid the possible dimerization of any of the GFP-tagged domains with endogenous ZNF384 as observed for GFP-ZNF384 and endogenous ZNF384 (Supplementary Fig. 7E). mCherry-NBS1 was co-expressed in these cells to control for DNA damage induction (Supplementary Fig. 7F). In agreement with our in vitro experiments, we found that the C2H2 domain was still recruited, while recruitment of the N-terminus and C-terminus was completely abolished (Fig. 3d). Consistently, we also found the colocalization between ZNF384 and DNA to be dependent on its C2H2 motif (Supplementary Fig. 6D).

Next, we sought to test the DNA-binding affinity of ZNF384-deletion mutants in vivo. We generated a ∆C2H2 mutant lacking the six C2H2 motifs, (∆C2H2), as well as mutants lacking the N-terminus (∆N-terminus) or C-terminus (∆C-terminus) (Supplementary Fig. 7G), and estimated the ability of full-length ZNF384 and ∆C2H2 to bind DNA using FRAP. In this assay, strong DNA-binding affinity corresponds to a slow FRAP recovery and vice versa. While full-length ZNF384 showed a slow recovery after photobleaching of the damaged area, we observed a fast recovery of ∆C2H2. In contrast, ∆N-terminus and ∆C-terminus showed a slower FRAP recovery compared to full-length ZNF384, suggesting that the deleted domains slightly destabilize ZNF384’s interaction with DNA (Fig. 3e and Supplementary Fig. 7H). We then assessed the recruitment of these GFP-tagged deletion mutants to DNA damage 405 laser micro-irradiation in Hoechst-sensitized *ZNF384* KO U2OS cells. Core histone H2B fused to photoactivatable PATagRFP (PTR) was co-expressed to define the damaged area. We found that ∆C2H2 dramatically impaired ZNF384 recruitment, while ∆N-terminus and ∆C-terminus had no major effect on recruitment (Fig. 3f). Collectively, these findings suggest that the C2H2 motif is important for DNA binding and accumulation of ZNF384 at DNA damage sites.

### ZNF384 modulates Ku70/Ku80 dynamics at DNA damage sites

The repair of DSBs by cNHEJ depends on the binding of the Ku70/Ku80 heterodimer to broken DNA ends, followed by the recruitment of the DNA-PKcs kinase (Mari, Florea et al.^32^). This in turn leads to the recruitment of the XRCC4–LIG4 complex, which ultimately seals the broken ends (Frit, Ropars et al.^33^). Given the interaction between ZNF384 and Ku70/Ku80, we sought to address if ZNF384 is involved in the loading of Ku70/Ku80 at DNA. To this end, we first monitored the levels of Ku70 in chromatin-enriched extracts from cells depleted of ZNF384 or Ku80 (Supplementary Fig. 8A). While *Ku80* knockdown reduced Ku70 levels on chromatin, depletion of ZNF384 had no impact (Supplementary Fig. 8A). Next, we monitored the impact of ZNF384 on Ku70 dynamics at DNA by FRAP. Within 2 seconds after photobleaching, we observed a small increase in fluorescence recovery of GFP-Ku70 in ZNF384-depleted cells compared to that in control cells (Supplementary Fig. 8B), suggesting that ZNF384 has a modest effect on Ku70’s DNA binding. To further investigate this finding, we assessed the DNA-binding affinities of both ZNF384 and Ku70 by comparing their relative residence times as measured by Fluorescence Correlation Spectroscopy (FCS) (Supplementary Fig. 8C). Interestingly, we observed that ZNF384 has a higher residence time compared to Ku70 (Supplementary Fig. 8D), suggesting that ZNF384 is more tightly bound to DNA, thereby impacting the association of Ku70/Ku80 with DNA.

**Fig. 4:**
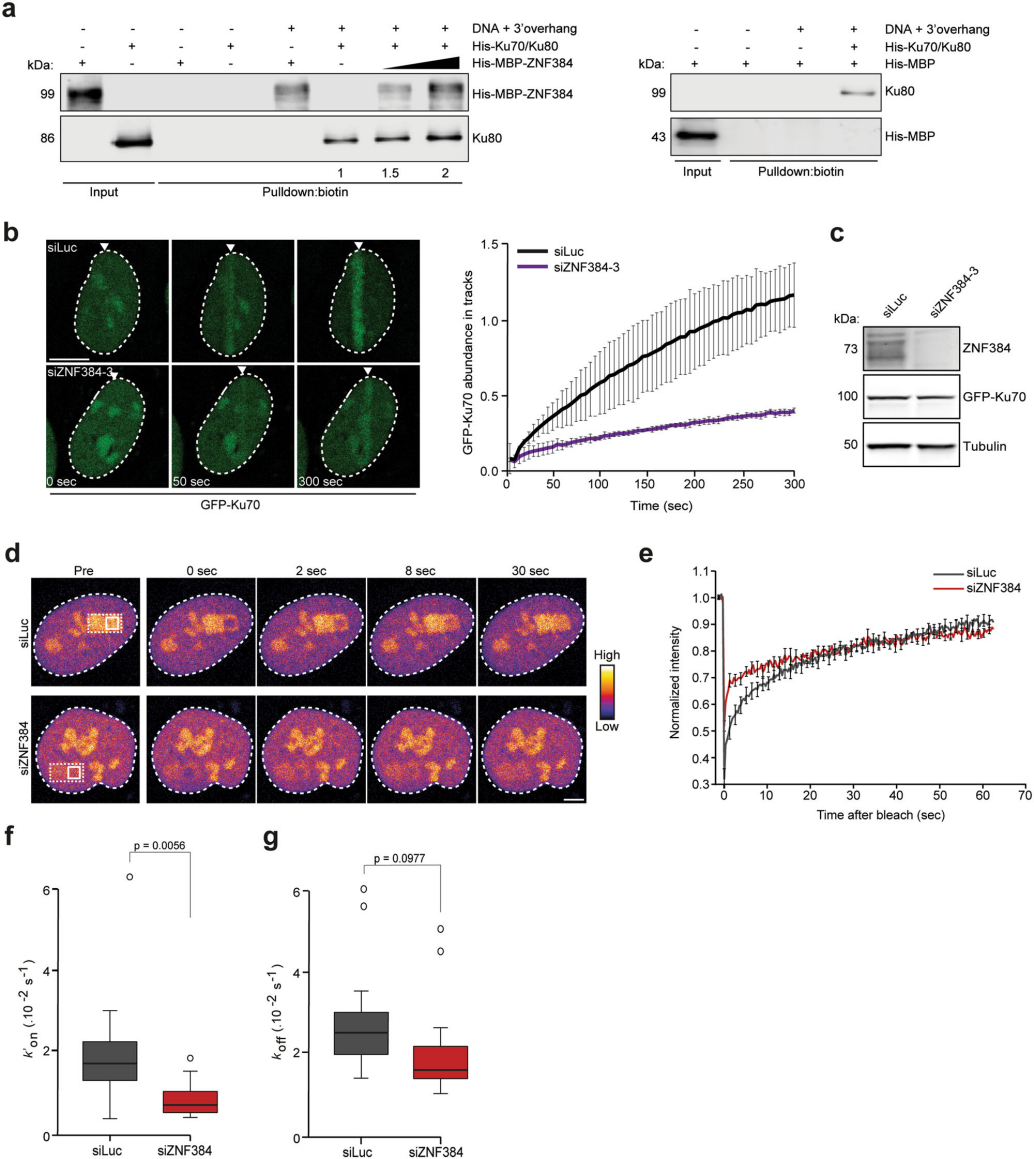
ZNF384 modulates Ku70/Ku80 dynamics at DNA damage sites. **a** DNA pull-downs of biotinylated DNA with a 3’-overhang in the presence of His-Ku70/Ku80, His-MBP-ZNF384, or His-MBP alone or His-Ku70/Ku80 in combination with His-MBP-ZNF384 or His-MBP. Blots were probed for MBP and Ku80. Ku80 pull-down signals were normalized to that in the pull-down lacking His-MBP-ZNF384, which was set to 1. The mean from four independent experiments is indicated below the blot. His-MBP was not detectable in pull-downs. A representative experiment is shown. **b** GFP-Ku70 recruitment to 800 nm multiphoton tracks in RPE1-hTERT cells transfected with the indicated siRNAs (left panel). White triangles indicate irradiated regions. Quantification of the data is presented as the mean ± SD from >60 cells acquired in two independent experiments. Scale bar 5 μm. **c** Western blot analysis of ZNF384 expression in cells from (**b**). Tubulin is a loading control. **d** Representative images of RPE1-hTERT cells transfected with the indicated siRNAs, in which FRAP measurements were performed to assess the local turnover of GFP-Ku70 at the sites of DNA damage. DNA damage was induced in the region indicated with a dashed line. Subsequent FRAP was induced in a subarea within the DNA damage region, as indicated with an unbroken line. Images are pseudocolored according to the look-up table displayed on the right. Scale bar 4 μm. **e** Normalized FRAP curves from (**d**). **f** Association (*k’*_on_) rates of GFP-Ku70 measured by FRAP after fitting of the curves from (**e**). **g** Dissociation (*k*_off)_ rates of GFP-Ku70 after fitting of the curves from (**e**). Data from (**e**–**g**) was collected from 15 cells per condition. Boxplot limits correspond to the 25th and 75th percentiles and the center line in the box indicates the median value from a representative of two independent replicates. Statistical significance was calculated using the unpaired Student’s *t* test, assuming unequal variances. Source data are provided as a Source Data file.

Based on these findings, we next examined whether ZNF384 has a potential stimulatory role on Ku70/Ku80’s binding to dsDNA with a 3′-overhang in vitro. To this end, we performed DNA pull-down assays using purified ZNF384 and Ku70/Ku80. Importantly, while Ku70/Ku80 bound dsDNA with a 3′-overhang in the absence of ZNF384^34^ their binding was enhanced in the presence of increasing amounts of ZNF384 (Fig. 4a and Supplementary Fig. 8E). To examine whether ZNF384 also affects the loading of Ku70/Ku80 at damaged DNA in vivo, we depleted ZNF384 in cells expressing endogenously GFP-tagged Ku70 (Britton, Coates et al.^35^), and subjected these cells to multiphoton laser micro-irradiation. Importantly, ZNF384 depletion reduced GFP-Ku70 accumulation at sites of DNA damage as compared to that in control cells (Fig. 4b, c). Given that ZNF384 is recruited to DNA breaks in a PAR-dependent manner (Fig. 2a) and regulates the loading of Ku70/Ku80, we asked whether Ku70/Ku80 is also recruited to sites of DNA damage in a PAR-dependent manner. Indeed, we found PARPi treatment to impair the recruitment of Ku70 to DNA breaks (Supplementary Fig. 8F), while the accumulation of the DNA damage marker NBS1 remained unaffected (Supplementary Fig. 8G, H). To better understand the impact of ZNF384 on the dynamics of Ku70, we assessed its turnover at DNA lesions by FRAP. Within 10 seconds after photobleaching, we observed a faster fluorescence recovery of GFP-Ku70 in ZNF384-depleted cells compared to that in control cells (Fig. 4d, e), suggesting that ZNF384 contributes to the association of Ku70 with DNA lesions. To be able to extract quantitative characteristics from the FRAP data, we first tried to fit the recovery curves with single-population models (Sprague, Pego et al.^36^). However, none of them could accurately fit the experimental curves (Supplementary Fig. 9A), indicating that Ku displays a more complex behavior. We then examined Ku dynamics by FCS as this method is able to assess fast protein turnover more accurately. The fit of the correlation curves showed that Ku follows two-population dynamics at DNA damage sites (Supplementary Fig. 9B). We infer that the fast population refers to Ku molecules diffusing through the nucleus and displaying only very transient interactions with chromatin, while the slow population corresponds to Ku molecules that bind DNA lesions more stably. To characterize specifically the behavior of this slow population, we restricted the fitting of the FRAP curves to the timepoints after 3 seconds post photobleaching, which could be well adjusted by a reaction-limited model (Supplementary Fig. 9C). Using this model, we were able to estimate binding (*k*′_on_) and unbinding (*k*_off_) rates of Ku at sites of DNA damage by FRAP. The *k*′_on_ is a pseudo-first-order association rate corresponding to the product of the actual binding rate *k*_on_ and the local density of DNA damage sites dictated by the irradiation conditions, which was similar between the different conditions. To confirm the applicability of this model, we also verified that the *k*_off_ parameter estimated with this model was independent of the size of the bleached area (Supplementary Fig. 9D). Based on these fits, we observed a reduction in the *k*′_on_ in ZNF384-depleted cells, suggesting a decreased Ku70-binding rate (Fig. 4f). In contrast, no significant impact of ZNF384 depletion on the estimated *k*_off_ was observed (Fig. 4g). Together these results imply that ZNF384 facilitates the recruitment and subsequent binding, rather than the retention of Ku70/Ku80, at sites of DNA damage.

### The C2H2 and N-terminal domains of ZNF384 are critical for loading Ku70/Ku80 at DSBs

**Fig. 5:**
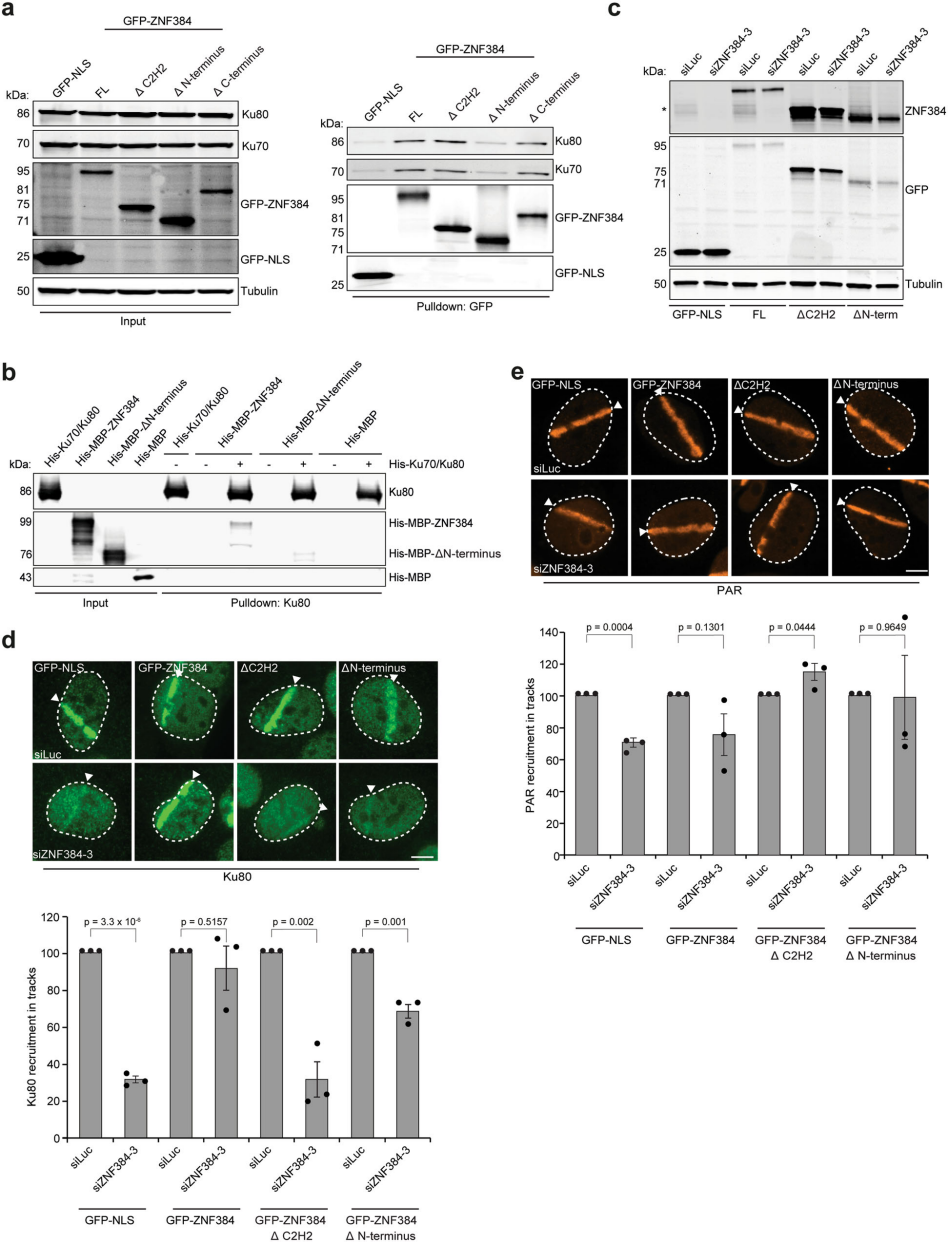
The C2H2 motifs and N-terminus of ZNF384 are required for Ku70/Ku80 loading at DSBs. **a** Pull-downs of the indicated GFP fusion proteins in U2OS Flp-In/T-Rex cells. Blots were probed for Ku70, Ku80, Tubulin, and GFP. The data shown represent three independent experiments. **b** In vitro Ku80 pull-down in the presence or absence of His-Ku70/Ku80 and His-MBP, His-MBP-ZNF384, or His-MBP-∆N-terminus. Control IP contained beads only. Blots were probed for Ku80 and MBP. The data shown represent two independent experiments. **c** Western blot analysis of the expression of endogenous ZNF384 and ectopic GFP-ZNF384 full-length and deletion mutants. Tubulin is a loading control. The asterisk (*) indicates endogenous ZNF384. The data shown represent three independent experiments. **d** Accumulation of endogenous Ku80 at 365 nm UV-A tracks in BrdU-sensitized U2OS Flp-In/T-Rex cells expressing siRNA-resistant doxycycline (dox)-inducible GFP-ZNF384, GFP-ZNF384 ΔC2H2, and GFP-ZNF384 ΔN-terminus following transfection with the indicated siRNAs. Cells were subjected to laser micro-irradiation and 10 min later fixed and immunostained. White triangles indicate irradiated regions (upper panel). Quantification of endogenous Ku80 levels in laser tracks is presented as ±SEM of >150 cells acquired in 3 independent experiments (lower panel). **e** As in (**d**), except for PAR (upper panel). Quantification of endogenous PAR levels in laser tracks is presented as the mean ± SEM from three independent experiments. Data were normalized to siLuc, which was set to 100% (lower panel). Statistical significance was calculated using the unpaired Student’s *t* test, assuming unequal variances. Scale bar 5 μm. Source data are provided as a Source Data file.

Having shown that ZNF384 promotes the loading of Ku70/Ku80 at DNA breaks and considering that ZNF384 and Ku70/Ku80 physically interact, we next sought to investigate whether the interaction between these proteins is important for the efficient recruitment of Ku70/Ku80 to DNA breaks. We therefore set out to map the region in ZNF384 that is required for the interaction with Ku70/Ku80, making use of our set of ZNF384-deletion mutants (Supplementary Fig. 7G). Using GFP pull-down assays, we found that GFP-tagged ZNF384, ∆C2H2, and ∆C-terminus associated with endogenous Ku70 and Ku80 with equal efficiency (Fig. 5a), suggesting that the C2H2 motifs and C-terminus are dispensable for the interaction. On the contrary, ∆N-terminus showed an almost complete loss of Ku70/Ku80 binding, indicating that the N-terminus of ZNF384 mediates its interaction with Ku70 and Ku80 (Fig. 5a). In agreement with our in vivo pull-down results, we found that recombinant ∆N-terminus bound less efficiently to Ku70/Ku80 when compared to recombinant ZNF384 (Fig. 5b).

We next asked whether the N-terminus of ZNF384 has any functional relevance for Ku70/Ku80 recruitment. To this end, we used the Flp-In/T-rex system to establish U2OS cells stably expressing inducible siRNA-resistant GFP-tagged versions of either ZNF384, ∆C2H2 or ∆N-terminus. U2OS cells stably expressing GFP-NLS served as a control (Fig. 5c). While expression of GFP-ZNF384 fully restored Ku80 accumulation at UV-A laser micro-irradiation inflicted damage, expression of ∆N-terminus only partially rescued this Ku80 defect (Fig. 5d). In contrast, the expression of ∆C2H2 completely failed to rescue the Ku80 accumulation defect (Fig. 5d). DNA damage induction was similar in all conditions based on equal PAR levels in laser tracks (Fig. 5e). These results suggest that the binding of ZNF384 at DNA breaks via its C2H2 motif, as well as the interaction between its N-terminus and Ku70/Ku80 contribute to efficient Ku80 recruitment.

Finally, we asked if ZNF384 is responsible for Ku70/Ku80 complex formation. To this end, we performed GFP pull-downs on cells expressing endogenously GFP-tagged Ku70 (Britton, Coates et al.^35^) that were depleted of ZNF384 and left untreated or exposed to ionizing radiation (IR). While we observed that GFP-Ku70 and endogenous Ku80 interact, as expected, loss of ZNF384 did not impact this interaction, neither in untreated nor in IR-exposed cells (Supplementary Fig. 10A). This suggests that ZNF384 is not involved in Ku70/Ku80 complex formation, but rather the loading of this complex at sites of DNA damage.

### ZNF384 promotes Ku-dependent loading of APLF and XRCC4/LIG4 at DSBs

**Fig. 6:**
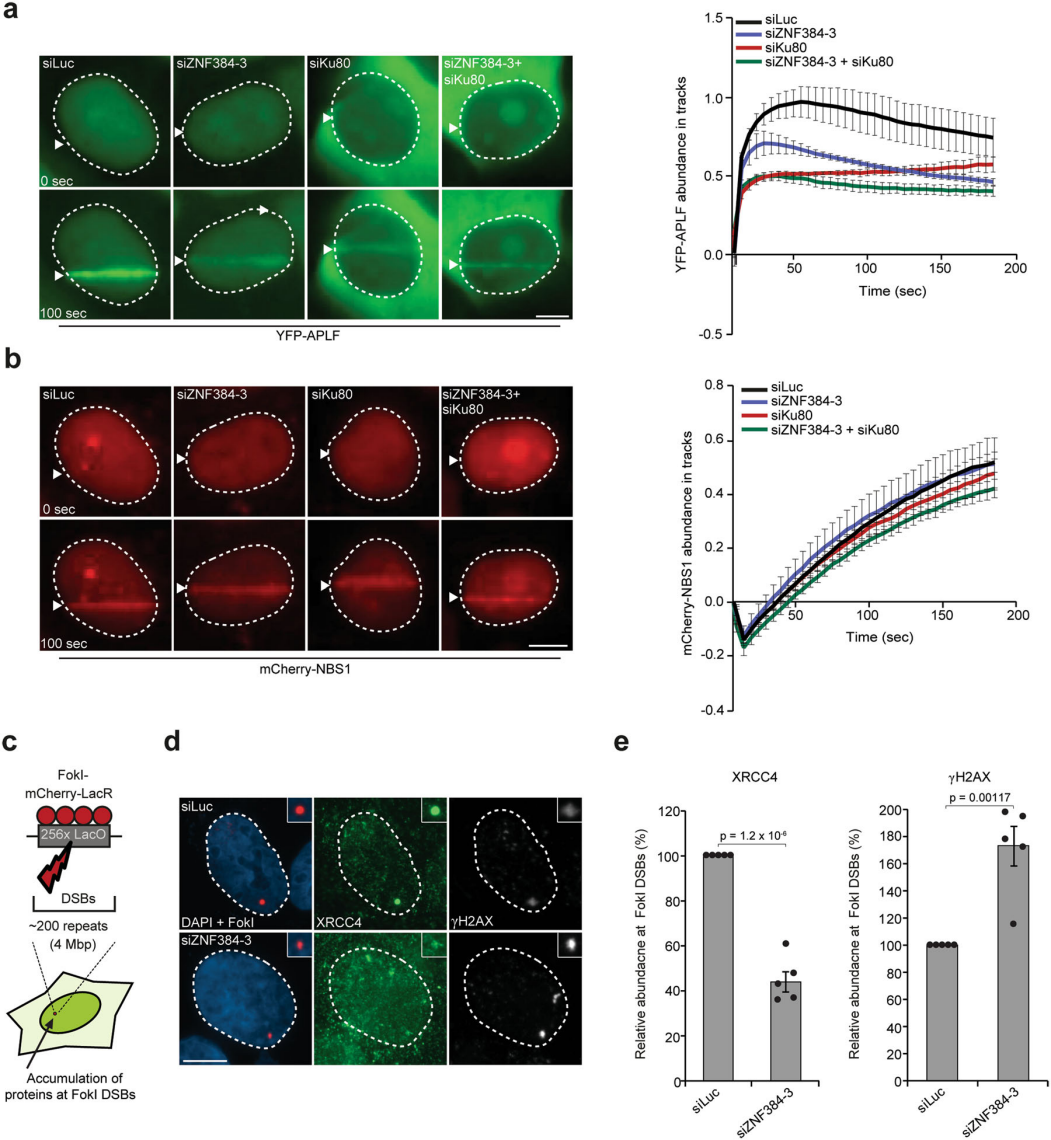
ZNF384 promotes Ku-dependent loading of APLF and XRCC4/LIG4 at DSBs. **a** Live-cell imaging of the recruitment of YFP-APLF to 365 nm UV-A tracks in BrdU-sensitized U2OS cells transfected with the indicated siRNAs. mCherry-NBS1, which was co-expressed with YFP-APLF, served as a DNA damage marker. Representative images are shown. White triangles indicate irradiated regions (left panel). Quantification of data is presented as the mean values ±SEM from 60 cells acquired in three independent experiments (right panel). **b** As in (**a**), except for mCherry-NBS1 (left panel). Quantification of data is presented as the mean values ± SEM from 60 cells acquired in three independent experiments (right panel). **c** Schematic of the system in U2OS 2-6-3 cells used to locally induce multiple DSBs upon tethering of the FokI endonuclease. **d** Accumulation of XRCC4 (green) to γH2AX-marked (white) DSBs induced by FokI-mCherry-LacR at a LacO array (red) in cells transfected with the indicated siRNAs. **e** Quantification of XRCC4 and γH2AX in cells from (**d**) is presented as the mean ± SEM of >200 cells acquired in five independent experiments. Data were normalized to siLuc control which was set to 100%. Statistical significance was calculated using the two-tailed unpaired Student’s *t* test, assuming unequal variances. Scale bar 5 μm. Source data are provided as a Source Data file.

Next, we asked if ZNF384 affects the accumulation of factors that act downstream of Ku70/Ku80. We first measured the recruitment of APLF, which physically interacts with Ku80 at DSBs via its conserved Ku-binding motif (KBM)^37^. To this end, YFP-tagged APLF and mCherry-NBS1 were co-expressed in ZNF384-, Ku80-, and ZNF384/Ku80-depleted U2OS cells, and examined for their localization at sites of DNA damage. ZNF384 and Ku80 depletion impaired APLF recruitment to sites of DNA damage, while the DNA damage marker NBS1 remained unaffected (Fig. 6a, b and ref. ^37^). Ku80 depletion also reduced nuclear retention of APLF^38^, which we validated in Ku80 knockdown cells (Supplementary Fig. 10B, C), as well as in *Ku80* KO mouse embryonic stem cells (Supplementary Fig. 10D). Interestingly, double knockdown of ZNF384 and Ku80 did not further impair APLF accumulation, suggesting that these proteins function epistatically to recruit APLF (Fig. 6a, b).

The fact that ZNF384 promotes the consecutive accumulation of Ku70/Ku80 and APLF made us wonder if ZNF384 could be involved in Ku-APLF complex formation. To assess this, we performed GFP pull-downs after transient expression of YFP-APLF in wild-type and ZNF384-depleted U2OS cells. Endogenous Ku80 was co-precipitated in both conditions (Supplementary Fig. 10E). Moreover, ZNF384 and Ku70 showed comparable recruitment kinetics (Supplementary Fig. S10F, G), while APLF was recruited earlier (Supplementary Fig. 10H). This suggests that ZNF384 does not impact Ku-APLF complex stability, but rather promotes the loading of these factors at sites of DNA damage.

The Ku-APLF interaction is critical for recruitment of the XRCC4/LIG4 complex to DNA beaks^3,37,39,40^. Given that ZNF384 promotes both Ku-APLF loading at sites of DNA damage, we next examined if ZNF384 also affects XRCC4 recruitment. To this end, we measured the levels of endogenous XRCC4 at bona fide DSBs induced by tethering of a Lactose repressor (LacR)-tagged FokI nuclease at a stably integrated Lactose operator (LacO) array in U2OS cells (Fig. 6c)^41^. While we detected XRCC4 accumulation at FokI-induced DSBs in control cells, as expected, its levels were dramatically reduced in ZNF384-depleted cells (Fig. 6d, e). This indicates that ZNF384 acts at bona fide DSBs to facilitate the Ku-APLF-dependent accumulation of XRCC4/LIG4 complexes.

### ZNF384 promotes recruitment of cNHEJ proteins independently of DNA-PKcs

Given that ZNF384 promotes the accumulation of XRCC4/LIG4 in a Ku70/Ku80-dependent manner, we wondered how this is linked to DNA-PKcs, which is recruited and activated by DSB-bound Ku to promote the loading of XRCC4/LIG4^42,43^. Western blot analysis detected induction of phosphorylated (p)-DNA-PKcs (S2056) after IR (Supplementary Fig. 11A). Surprisingly, neither Ku80 nor ZNF384 depletion affected p-DNA-PKcs (S2056) activation in U2OS cells (Supplementary Fig. 11A). Moreover, normal levels of p-DNA-PKcs (S2056) activation were observed in *Ku80* KO mouse embryonic stem cells, ruling out that the lack of phenotype in Ku80-depleted cells was due to incomplete knockdown (Supplementary Fig. 11B and ref. ^44^. To corroborate these findings, we examined the interplay between ZNF384, Ku, and DNA-PKcs during the loading of XRCC4 at sites of DNA damage. We found that ZNF384, Ku80, and DNA-PKcs depletion impaired XRCC4 accumulation (Supplementary Fig. 11C–F), which is in line with previous results^42,45^. Double knockdown of ZNF384 and Ku80 did not result in an additive effect on XRCC4 accumulation when compared to that in ZNF384- or Ku80-depleted cells (Supplementary Fig. 11C–F). This suggests that these proteins act epistatically to recruit XRCC4, which is in line with their epistatic role in recruiting APLF to DNA breaks (Fig. 6a, b). In contrast, double knockdown of ZNF384 and DNA-PKcs resulted in a larger effect on XRCC4 accumulation when compared to that of ZNF384 or DNA-PKcs depletion alone (Supplementary Fig. 11C–F), suggesting redundant functions for these proteins in XRCC4 recruitment.

Given that ZNF384 and Ku were recruited to sites of DNA damage via PAR-driven processes (Fig. 2a and Supplementary Fig. 8F), and that ZNF384 and DNA-PKcs function redundantly, we next asked whether DNA-PKcs is also recruited to DNA breaks in a PAR-dependent manner. Indeed, PARPi treatment impaired the recruitment of DNA-PKcs (Supplementary Fig. 11G), while the accumulation of the DNA damage marker NBS1 remained unaffected (Supplementary Fig. 11H, I). To further understand the interplay between ZNF384, Ku and DNA-PKcs with PARP1, epistasis analysis were performed. We found that ZNF384, DNA-PKcs, and PARP1 depletion impaired XRCC4 accumulation, which is in line with our previous results (Supplementary Fig. 12A, B and ref. ^9^). However, double knockdown of either ZNF384 or DNA-PKcs with PARP1 did not result in an additive effect on XRCC4 accumulation (Supplementary Fig. 12C, D). Together these results suggest that PARP activity drives two parallel pathways for DSB repair by NHEJ, one of which relies on the ZNF384-mediated ligation of broken ends via Ku-APLF-XRCC4, the other on DNA-PKcs-XRCC4.

### ZNF384 promotes DSB repair via cNHEJ

**Fig. 7:**
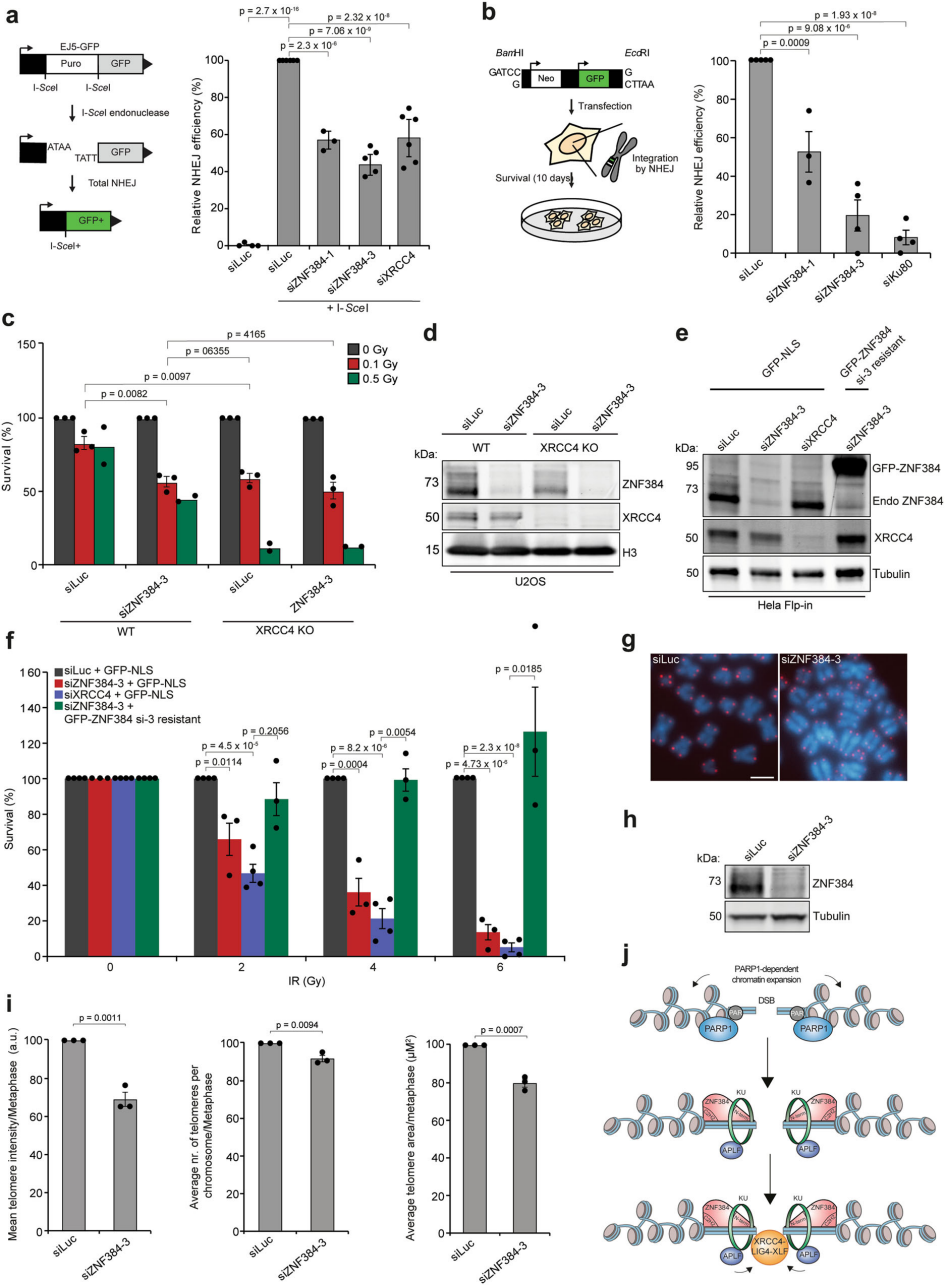
ZNF384 promotes DSB repair via cNHEJ. **a** Schematic of the EJ5-GFP reporter for NHEJ (left panel). Quantification of EJ5-GFP-positive U2OS cells transfected with the indicated siRNAs and I-*Sce*I expression vector. I-*Sce*I transfection was corrected by co-transection with mCherry expression vector. The mean ± SEM of 3–5 independent experiments is shown (right panel). Data were normalized to the siLuc control which was set to 100%. **b** Schematic of the random plasmid integration assay (left panel). Quantification of plasmid integration efficiencies in U2OS cells transfected with the indicated siRNAs (right panel). The mean ± SEM of 3–6 independent experiments is shown. Data were normalized to siLuc control which was set to 100%. **c** Relative survival efficiency in WT and *XRCC4* KO U2OS cells transfected with the indicated siRNAs and exposed to the indicated doses of IR. The mean ± SEM of 2–3 independent experiments is shown. Data were normalized to unirradiated conditions and set to 100%. **d** Western blot analysis of the expression of endogenous ZNF384 from cells in (**c**). Tubulin is a loading control. **e** Expression levels of endogenous ZNF384 and dox-inducible siRNA-resistant GFP-ZNF384 in Hela Flp-In/TRex cells. Tubulin is a loading control. **f** Effect of inducible expression of GFP-NLS and siRNA-resistant GFP-tagged ZNF384 on the survival of stable Hela Flp-In/TRex after transfection with indicated siRNAs and IR treatment. The mean ± SEM of 3–4 independent experiments is shown. Data were normalized to siLuc control which was set to 100%. **g** Representative FISH images of metaphases from HCT116 cells transfected with the indicated siRNAs. Scale bar 5 μm **h** Western blot analysis of the expression of endogenous ZNF384 from cells in (**g**). Tubulin is a loading control. **i** Quantifications per metaphase from (**h**) are presented as the mean ± SEM of 75 chromosomes acquired in three independent experiments. Data were normalized to siLuc control, which was set to 100%. **j** Model for how ZNF384 works as an adaptor of Ku to DNA during DSB repair by cNHEJ (see text for details). All *P* values were calculated using the two-tailed unpaired Student’s *t* test, assuming unequal variances. Source data are provided as a Source Data file.

The interaction between ZNF384 and cNHEJ factors (Fig. 1d), its PARP/PAR-dependent recruitment (Fig. 2a), as well as its ability to load Ku (Fig. 4b), APLF (Fig. 6a), and XRCC4/LIG4 (Fig. 6e) at DSBs, encouraged us to assess whether ZNF384 supports DSB repair via NHEJ. To this end, we first used the well-established EJ5-GFP reporter assay, which relies on the restoration of GFP expression following repair of I-*Sce*I endonuclease-induced DSBs that flank a puromycin gene that separates a GFP gene from a CMV promoter (Fig. 7a). Flow cytometric analysis of GFP fluorescence revealed that NHEJ was reduced following ZNF384 knockdown, which was comparable to the effect observed upon XRCC4 knockdown (Fig. 7a). Cell cycle profiles remained unaffected in these cells, ruling out the effects of cell cycle misregulation (Supplementary Fig. 13A, B). Importantly, knockdown of ZNF384 did not affect the steady-state levels of several factors involved in NHEJ (Supplementary Fig. 13C), albeit that the expression of XRCC4 was slightly reduced (Supplementary Fig. 13C). However, a semi-quantitative analysis revealed that this effect was not consistent, neither in U2OS (Supplementary Fig. 13D) nor in HeLa Flp-In cells (Supplementary Fig. 13E), suggesting that indirect effects due to transcriptional misregulation are unlikely. Furthermore, ZNF384 depletion did not have a major impact on the steady-state levels of the checkpoint kinases ATM and CHK1 (Supplementary Fig. 13F–H), nor affected the IR-induced phosphorylation of ATM (at S1981) and CHK1(at S345) (Supplementary Fig. 13I, J), the latter of which was used as a readout for ATR activation^46^. This suggests that ZNF384 does not contribute to DSB repair by regulating ATM or ATR activation.

The EJ5-GFP reporter provides a readout for total NHEJ activity, including cNHEJ and aNHEJ^47^. To address whether ZNF384 specifically affects Ku70/Ku80-, APLF-, and XRCC4/LIG4-dependent NHEJ, we measured random plasmid integration into the genome via cNHEJ (Fig. 7b and refs. ^9,48^). Indeed, ZNF384 knockdown, similar to that of Ku80 depletion, impaired random plasmid integration, indicating an important role for ZNF384 in cNHEJ (Fig. 7b).

Next, we asked whether ZNF384 plays a unique role in cNHEJ or also affects HR. Interestingly, loss of ZNF384 only very moderately impaired HR in the well-established DR-GFP reporter (Supplementary Fig. 14A), and rendered cells only mildly sensitive to treatment with PARPi (Supplementary Fig. 14B, C), in contrast to HR-deficient cells depleted of BRCA1^49^. Given that ZNF384 is critical for Ku70/Ku80 loading at DSBs and that loss of Ku70/Ku80 has been linked to increased DNA-end resection and HR levels^50,51^, we assessed whether ZNF384 affects DNA-end resection. Cells depleted of ZNF384 did not show a significant difference in RPA foci number and foci intensity (Supplementary Fig. 14D–G), which is consistent with the fact that ZNF384 loss did not impact HR (Supplementary Fig. 14A). Ku80-depleted cells also did not show changes in DNA-end resection (Supplementary Fig. 14D–G), which is in agreement with another report showing that end resection remained unaffected in *Ku70/Ku80* double KO MEF cells^52^. Corroborating these findings, we observed that the accumulation of the core HR protein RAD51 into DSB-containing foci induced by IR was not affected in ZNF384-depleted S-phase cells (Supplementary Fig. 14H). Together these results suggest that ZNF384 does not play a major role in DSB repair via HR, but rather promotes cNHEJ.

When cNHEJ is compromised, DSB repair mostly occurs via aNHEJ, causing a loss of accurate end-joining and a switch to error-prone repair due to deletion formation and microhomology usage^53^. To test whether impaired cNHEJ in ZNF384-depleted cells impacts the mutational signature at repair junctions, we used a previously published NHEJ reporter in GC92 cells^54^. This reporter consists of a CD4 gene separated from a CMV promoter by an H2Kd-CD8 cassette that is flanked by I*-Sce*I cleavage sites. DSB induction by I-*Sce*I expression leads to re-ligation of the CMV promoter to the CD4 gene after which repair junctions can be amplified and Sanger-sequenced (Supplementary Fig. 15A). Indeed, depletion of the cNHEJ factor Ku80 resulted in an increase in the formation of larger deletions and usage of larger stretches of microhomology (Supplementary Fig. 15B, C). Moreover, although ZNF384 depletion only caused a modest effect on the total deletion frequency, among these events the proportion of larger deletions and use of microhomology during repair was increased and resembled that observed after Ku80 depletion (Supplementary Fig. 15B, C), corroborating a role for ZNF384 in cNHEJ.

In further support of these findings, we found that ZNF384 loss impaired clonogenic survival of U2OS cells and VH10-SV40-immortalized fibroblasts following exposure to IR-induced DSBs similar to that observed after XRCC4 depletion (Fig. 7c, d and Supplementary Fig. 15D, E). Interestingly, double depletion of ZNF384 and XRCC4 did not result in an increased sensitivity to IR (Fig. 7c, d), again indicating that these proteins function epistatically to promote cNHEJ-dependent repair DSBs. Finally, we employed the Flp-In/T-Rex system to establish HeLa cells stably expressing inducible siRNA-resistant GFP-tagged ZNF384 or GFP-NLS (Fig. 7e). We found that expression of siRNA-resistant GFP-ZNF384, but not that of GFP-NLS, almost fully rescued the IR sensitivity observed after ZNF384 loss, the latter of which being comparable to that observed after XRCC4 loss (Fig. 7f). This indicates that the IR sensitivity and underlying cNHEJ defect are not caused by off-targets of the siRNAs against ZNF384. The increase in IR sensitivity likely resulted from an accumulation of unresolved DSBs, as indicated by the increase in γH2AX foci following IR exposure of ZNF384-depleted G1 cells, which resembled the phenotype of Ku80-depleted cells (Supplementary Fig. 15F–H^55^). Interestingly, Ku80 has been implicated in telomere length maintenance (Jaco, Muñoz et al., 2004^56^). To study whether ZNF384 is similarly involved in this process, we performed fluorescence in situ hybridization (FISH) using a PNA probe to label all telomeres and use their intensity as a proxy for telomere length (Fig. 7g)^57^. Strikingly, knockdown of ZNF384, similar to that of Ku80^56^, significantly reduced telomere length in metaphase spreads when compared to that in control cells (Fig. 7h, i). Collectively, our results demonstrate that ZNF384 promotes efficient DSB repair via cNHEJ and is involved in telomere length maintenance (Fig. 7j).

## Discussion

In this study, we uncover an important role of the poorly characterized ZnF protein ZNF384 in DSB repair via cNHEJ (Fig. 7j). First, we demonstrate that ZNF384 is recruited to sites of DNA damage and interacts with Ku70/Ku80 and PARP1. Second, we show that ZNF384 recruitment requires PARP1/PAR-dependent chromatin remodeling, which promotes the binding of ZNF384 to the exposed DNA via its C2H2 motifs. Third, ZNF384 stimulates the binding of Ku70/Ku80 at DNA breaks, on the one hand through physical interaction with this complex, and on the other hand through its affinity for DNA. This way, it promotes the assembly of a functional cNHEJ complex that includes APLF and the XRCC4/LIG4 complex. Finally, ZNF384 promotes NHEJ in EJ5-GFP reporter assays and random plasmid integration assays, and functions epistatically with both Ku and XRCC4 during this repair process. Thus, ZNF384 functions as an “adaptor station” for the proper assembly of repair proteins at DSBs, thereby promoting efficient repair by cNHEJ (Fig. 7j).

### PARP1/PAR-dependent chromatin unfolding allows ZNF384 binding to damaged DNA

Our findings reveal that ZNF384 is recruited to sites of DNA damage following chromatin unfolding driven by the activity of PARP1, but not PARP2 or PARP3. Several other cNHEJ repair proteins, including Ku70/Ku80 and XRCC4, are also recruited in a manner dependent on the activity of PARP1. This may involve their binding to PARP1-associated PAR chains or to the damaged DNA itself^9,58^. Using three independent approaches, we confirmed that ZNF384 does not bind to PARP1-associated PAR chains at sites of DNA damage. Instead, ZNF384 recruitment occurs in a manner dependent on the PARP1-induced relaxation of the damaged chromatin, making the DNA available for ZNF384 to bind via its C2H2 DNA-binding motif. This is consistent with other reports showing that ZNF384 directly binds to DNA, particularly to homopolymeric dA·dT consensus sequences in vitro^31,59^. We confirmed and extended this finding by showing that ZNF384, through its C2H2 domain, also efficiently binds dsDNA substrates containing 5′- or 3′-overhangs as opposed to dsDNA with blunt ends. DSBs with such protruding ends have been shown to be preferentially repaired by cNHEJ, while blunt ends are mostly subjected to polymerase theta-mediated end-joining^44^. Thus, the preferential binding of ZNF384 to dsDNA with 5′- or 3′-overhangs is consistent with its role in cNHEJ. However, it is important to note that the DNA substrates used in our study also contain T-rich consensus sequences. These sequences are considered the most abundant simple repetitive motifs in the human genome that are frequently expanded due to DNA replication slippage^60^. The fact that DSBs can occur throughout the genome, including at homopolymeric dA·dT repeats, raises the question whether the C2H2 DNA-binding domain of ZNF384 binds to a specific DNA context or binds to lesions in any given sequence context, the latter of which would be in line with a more general role of ZNF384 in cNHEJ. Elucidating the nature of its DNA sequence-specific binding mode will be key in further unraveling how ZNF384 acts at DSBs to promote cNHEJ.

### ZNF384 serves as a “Ku-adapter” at sites of DNA damage

ZNF384 promotes the accumulation of Ku70/Ku80, but not their retention at sites of damage. This raises the question how the recruitment of these proteins is regulated at the level of DNA binding. FCS analysis revealed that ZNF384 had a significantly higher residence time compared to Ku70, suggesting that ZNF384 binds stronger to DNA as compared to Ku70. Moreover, ZNF384 stimulates the binding of Ku70/Ku80 on DNA in vitro, suggesting that ZNF384 is the dominant binding force within the ZNF384–Ku complex, in which it serves as a platform that assists in the positioning of Ku70/Ku80 on DNA. Indeed, we identified two regions in ZNF384 that contribute to Ku70/Ku80 recruitment at sites of DNA damage: the N-terminus, which mediates the interaction with Ku70/Ku80, and the internal C2H2 motifs, which ensure DNA binding. The region in Ku70/Ku80 that is responsible for the Ku-ZNF384 interaction, as well as its relevance for cNHEJ, remains to be established. Collectively, these findings suggest that ZNF384 may act as a “Ku-adapter” that (1) senses DNA damage, (2) binds to DNA upon PARP/PAR-induced chromatin relaxation, and (3) guides Ku70/Ku80 for efficient loading at DNA breaks. To further support this model, which suggests a co-operative mode of action between ZNF384 and Ku70/Ku80, future studies may focus on understanding the spatio-temporal dynamics of these proteins at individual DSBs.

### ZNF384 and DNA-PKcs act redundantly during cNHEJ protein assembly

How does ZNF384 promote the proper Ku-dependent build-up of downstream NHEJ proteins at sites of DNA damage? ZNF384 and Ku80 act epistatically to promote APLF recruitment to sites of DNA damage. Moreover, a direct interaction between APLF and Ku’s conserved KBM region has previously been shown to promote XRCC4 recruitment^37^. Our data reveal that ZNF384 is not implicated in Ku-APLF complex formation, instead suggesting that ZNF384 loads XRCC4 at DSBs by promoting the recruitment of Ku70/Ku80 and thereby also APLF. Although our mass-spectrometry analysis for ZNF384-interacting proteins did not detect APLF, we cannot exclude the possibility that physical interactions between these proteins also contribute to XRCC4/LIG4-dependent cNHEJ.

Previous work suggested that XRCC4/LIG4 assembly also depends on the recruitment and activation of DNA-PKcs by Ku-bound DNA ends^61^. However, DNA-PKcs activity remained unaffected in ZNF384- or Ku80-depleted human cells, as well as in *Ku80* KO mouse embryonic stem cells. This raises the question whether ZNF384 and Ku70/Ku80 promote XRCC4/LIG4 accumulation independently of DNA-PKcs. Our epistasis analysis suggests that ZNF384 cooperates with Ku70/Ku80, but functions independently of DNA-PKcs to promote XRCC4 accumulation at DNA breaks. This is in line with a recent report showing that Ku and XRCC4/LIG4 are sufficient for DNA-end synapsis independently of DNA-PKcs in vitro^62^. Furthermore, it may be possible that APLF dictates the functional redundancy between ZNF384 and DNA-PKcs, as APLF is recruited to sites of DNA damage via Ku, PARP3^39,40^, and ZNF384 to promote the loading of XRCC4. Finally, DNA-PKcs has been reported to have additional roles beyond NHEJ such as in mitosis, during which DNA-PKcs autophosphorylation appears to be largely independent of Ku^63,64^. This suggests the existence of Ku and DNA-PKcs independent mechanisms and may explain the redundancy of ZNF384 and DNA-PKcs during cNHEJ. Future work may not only provide more insight into how ZNF384 functions independently of DNA-PKcs during the assembly of a functional cNHEJ repairosome, but would also help to deepen our understanding of how DNA-PKcs function is linked to cNHEJ driven by ZNF384-Ku70/Ku80.

### ZNF384 and other ZnF proteins in cNHEJ

We reveal an important role for ZNF384 in stimulating efficient cNHEJ in human cells. However, ZNF384 is not the only ZnF protein involved in cNHEJ. For instance, APLF and ZBTB24 possess distinct ZnF domains (PBZ in APLF and C2H2 in ZBTB24) that are required for the build-up of a functional NHEJ complex by binding to auto-mono(ADP-ribosyl)ated (MAR) PARP3, PARylated PARP1, or DNA, respectively, at DSBs^18,37,65^. This suggests that the versatile substrate recognition ability by distinct domains in ZnF proteins may play an important role in the cNHEJ process. To this end, it is interesting to note that several other ZnF proteins are recruited to sites of DNA damage in a PAR-dependent manner^12^. Although it remains to be established whether this involves direct PAR binding or binding to damaged DNA, these findings suggest that ZnF proteins may play a more important role in DNA repair than previously anticipated. Future mechanistic studies will undoubtedly improve our understanding of their crucial role in diverse biological processes, including DNA damage repair, thereby increasing our understanding of genome stability maintenance.

## Methods

### Cell lines

U2OS, HeLa, VH10-SV40, and SV40 T-transformed GM639 human fibroblasts cells were cultured in 5% CO_2_ at 37 °C in DMEM (Dulbecco’s modified Eagle’s medium), and DMEM and DMEM F-12 (Ham) supplemented with 10% fetal calf serum and antibiotics. RPE1-hTERT cells expressing endogenous GFP-KU70 were a gift from Steve Jackson^35^. U2OS cells with stably integrated EJ5-GFP or DR-GFP reporters were a gift from Jeremy Stark and Maria Jasin^47,66^. SV40 large T-transformed GM639 human fibroblasts with a stably integrated GC92 reporter were a gift from Bernard Lopez^54^. U2OS cells stably expressing cell cycle marker mKO-Cdt1 were previously generated^9^. U2OS AsiSI-ER- cells were a gift from Gaelle Legube^67^. U2OS 2-6-5 cells stably expressing ER-mCherry-LacR-FokI-DD were a gift from Roger Greenberg^41^. HeLa and stable GFP-Ku80 expressing HeLa cells were a gift from Dik van Gent (Erasmus Medical Center, Rotterdam, the Netherlands). WT and *Ku80* KO 129/Ola-derived IB10 mouse embryonic stem cells were a kind gift from Marcel Tijsterman^44^. PARP1, PARP2, and PARP1/PARP2 knockout U2OS cells were a kind gift from Nicholas Lakin^68^. XRCC4 knockout U2OS cells were generated by co-transfection of pKLV-U6gRNA-EF(BbsI)-PGKpuro2ABFP (Addgene) containing XRCC4 gRNA (5′-GATGACATGGCAATGGAAA-3′) with pSpCas9(BB)-2A-GFP (PX458) containing Cas9 (Addgene). ZNF384 knockout U2OS Flp-In/T-rex cells were generated by co-transfection of pKLV-U6gRNA-EF(BbsI)-PGKpuro2ABFP (Addgene) containing ZNF384 gRNA (5′-CCACCTCTGAGAACAGGAGACTC-3′) with pSpCas9(BB)-2A-GFP (PX458) containing Cas9 (Addgene). HeLa Flp-In/T-Rex and U2OS Flp-In/T-Rex cells, which were generated using the Flp-In/T-REx system (ThermoFisher Scientific), were a gift of Geert Kops (University Medical Center Utrecht, the Netherlands) and Stephen Taylor (University of Manchester, UK). These cells were used to stably express inducible versions of GFP-NLS and GFP-APLF as well as siRNA-resistant GFP-ZNF384, GFP-∆N-terminus (1-209), GFP-∆C2H2 (205-410)^∆^, GFP-∆C-terminus (401-516)^∆^ by co-transfection of pCDNA5/FRT/TO-Puro plasmid encoding GFP or GFP-tagged ZNF384 and deletion mutants (5 µg), together with pOG44 plasmid encoding the Flp recombinase (1 µg). After selection on 1 µg/mL puromycin, single clones were isolated and expanded. Both HeLa Flp-In/T-REx clones and U2OS Flp-In/T-Rex were incubated with 2 µg/mL doxycycline for 24 h to induce expression of cDNAs. All cells were authenticated by STR profiling and tested negative in routinely performed mycoplasma tests.

### Chemicals

Cells were treated with Phleomycin (InvivoGen) at the indicated concentrations for 1 h and collected for further analysis. The PARP inhibitor olaparib (Selleck Chemicals) and DNA-PK inhibitor NU-7441 (Selleck Chemicals) were both used at a final concentration of 10 μM, whereas PARGi (PDD00017273, Sigma) inhibitor was used at a concentration of 25 μM. H_2_O_2_ was used at a concentration of 0.5 mM.

### Transfections, siRNAs, and plasmids

Cells were transfected with siRNAs using RNAiMAX (Invitrogen) according to the manufacturer’s instructions. Cells were transfected twice with siRNAs at 0 and 24 h at a concentration of 40 nM and analyzed 48 h after the second transfection unless otherwise indicated. siRNA sequences are listed in Supplementary Table 2. Cells were transfected with plasmid DNA using Lipofectamine 2000 (Invitrogen) according to the manufacturer’s instructions and analyzed 24–48 h after transfection. The expression vector for full-length human ZNF384 (pCDNA3.1-FLAG-ZNF384-WT, isoform 2 with six C2H2 motifs), which was a gift from Myriam Alcalay^69^, was amplified and cloned into pCDNA5/FRT/TO-Puro as a *HindIII/KpnI* fragment (Supplementary Table 3). Deletion constructs were generated by site-directed mutagenesis PCR (Supplementary Table 3). siZNF384-3-resistant ZNF384 cDNA was generated by introducing the underlined mutations CGACAGCATAATAAGGACAAG by overlap PCR and cloned as *HindIII/KpnI* fragment into pCDNA5/FRT/TO-Puro-ZNF384-WT (Supplementary Table 2). All ZNF384 expression constructs were verified using Sanger sequencing. All other plasmids were described previously: pmCherry-PARP1^70^, pmEGFP-macroH2A1.1 macrodomain^11^, GFP-WWE (from RNF146)^8^, H2B-PTR, GFP-BZIP^11^, GFP-CHD4 and YFP-APLF^71^.

### Generation of DSBs by ionizing radiation (IR)

IR was delivered to cells by an YXlon X-ray generator machine (200 KV, 4 mA, dose rate 1 Gy/min) or a Faxitron Cabinet X-ray System Model RX-650 (130 kVp, dose rate 1.85 Gy/min).

### 365 nm UV-A laser micro-irradiation

Cells were grown on 18 mm coverslips and sensitized with 10 µM 5′-bromo-2-deoxyuridine (BrdU) for 24 h as described^9^. For micro-irradiation, the cells were placed in a Chamlide TC-A live-cell imaging chamber that was mounted on the stage of a Leica DM IRBE wide-field microscope stand (Leica) integrated with a pulsed nitrogen laser (Micropoint Ablation Laser System; Andor). The pulsed nitrogen laser (16 Hz, 364 nm) was directly coupled to the epifluorescence path of the microscope and focused through a Leica 40x HCX PLAN APO 1.25–0.75 oil-immersion objective. The growth medium was replaced by CO_2_-independent Leibovitz’s L15 medium supplemented with 10% FCS and cells were kept at 37 °C. The laser output power was set to 72–80 to generate strictly localized sub-nuclear DNA damage. Cells were micro-irradiated (two iterations per pixel) within 5 min using Andor IQ software (version 3.6). Following micro-irradiation, cells were incubated for the indicated timepoints at 37 °C in Leibovitz’s L15 and subsequently fixed with 4% formaldehyde before immunostaining. Images of fixed samples were acquired on a Zeiss AxioImager M2 or D2 wide-field fluorescence microscope equipped with ×40, ×63, and ×100 PLAN APO (1.4 NA) oil-immersion objectives (Zeiss), an HXP 120 metal-halide lamp used for excitation and the following filters: DAPI (excitation filter: 350/50 nm, dichroic mirror: 400 nm, emission filter: 460/50 nm), GFP/Alexa 488 (excitation filter: 470/40 nm, dichroic mirror: 495 nm, emission filter: 525/50 nm), mCherry (excitation filter: 560/40 nm, dichroic mirror: 585 nm, emission filter: 630/75 nm), Alexa 555 (excitation filter: 545/25 nm, dichroic mirror: 565 nm, emission filter: 605/70 nm), Alexa 647 (excitation filter: 640/30 nm, dichroic mirror: 660 nm, emission filter: 690/50 nm). Images were recorded using ZEN 2012 software (blue edition, version 1.1.0.0) and analyzed in ImageJ (version 1.48) as described previously (Luijsterburg, de Krijger et al.^9^). Briefly, the average pixel intensity of laser tracks was measured within the locally irradiated area (Idamage), in the nucleoplasm outside the locally irradiated area (Inucleoplasm), and in a region not containing cells in the same field of view (Ibackground). The level of protein accumulation relative to the protein level in the nucleoplasm was calculated as follows: ((Idamage −  Ibackground)/(Inucleoplasm − Ibackground) – 1).

### 405 nm laser micro-irradiation

Laser micro-irradiation for local photoactivation and DNA damage induction at 405 nm was performed using a single-point scanning head (iLas2 from Roper Scientific) coupled to the epifluorescence backboard of a Nikon Ti-E inverted microscope equipped with a spinning-disk scan head CSU-X1 from Yokogawa at a rotation speed of 5000 rpm, a Plan APO ×60/1.4 N.A oil-immersion objective lens and a sCMOS ORCA Flash 4.0 camera. The fluorescence of EGFP and mCherry/activated PATagRFP were excited with lasers at 490 and 561 nm, respectively. Bandpass filters adapted to the fluorophore emission spectra were used for fluorescence detection. Images were acquired using Metamorph software (version 7.8.2.0). Cells were sensitized with media containing 0.3 μg/mL Hoechst 33342 for 1 h at 37 °C. Prior to imaging, the medium was replaced with CO_2_-independent phenol red-free Leibovitz’s L15 medium (Life Technologies) supplemented with 20% FCS. Cells were irradiated with a 16-μm line through the nucleus to simultaneously induce DNA damage and photoactivate PATagRFP. The 405 nm laser power was measured at the beginning of each experiment and set to 125 μW at the sample level to ensure reproducibility. For PAR-3H experiments and ZNF384 recruitment experiments, images were collected every 5 s for 10 min. For ZNF384 and WWE recruitment with late PARP inhibitor treatment, Z-stacks (1-μm steps) of irradiated nuclei were collected every 30 s for 15 min. Image collection was paused 3 min post damage and olaparib was added to the imaging media to a final concentration of 30 μM. For protein recruitment analysis, a custom-made MATLAB (MathWorks) program R2014b (version 8.4.0.150421) (available upon request). For reviewing purposes, the following link can be used: https://github.com/sehuet/Singh-image-processing was used to segment the site of damage *(I*_*d*_) as determined by the photoactivated H2B area, the total nuclear fluorescence (*I*_*nd*_), and an area of background outside of the cell (*I*_*bg*_). Protein accumulation at sites of damage (*A*_*d*_) was calculated as:

1 $${A}_{d}=\frac{{I}_{d}-{I}_{{bg}}}{{I}_{n}-{I}_{{bg}}}$$

The intensity within the micro-irradiated area was then normalized to the intensity prior to damage induction. Chromatin relaxation was determined by measuring the change in thickness of the photoconverted H2B line^8^.

### Multiphoton laser micro-irradiation

Cells were grown on 18-mm coverslips. For micro-irradiation, cells were placed in a Chamlide CMB magnetic chamber and the growth medium was replaced by CO_2_-independent Leibovitz’s L15 medium supplemented with 10% FCS and antibiotics. Laser micro-irradiation was performed on a Leica SP5 confocal microscope equipped with an environmental chamber set to 37 °C. DNA damage-containing tracks (1.5-μm width) were generated with a Mira mode-locked titanium-sapphire (Ti:Sapphire) laser (*l* = 800 nm, pulse length = 200 fs, repetition rate = 76 MHz, output power = 80 mW) using a UV-transmitting 63 × 1.4 NA oil-immersion objective (HCX PL APO; Leica). Confocal images were recorded before and after laser irradiation at 5-s time interval over a period of 3–5 min. Images after multiphoton micro-irradiation of living cells were recorded using LAS-AF software (Leica, light version 1.0.0) and analyzed with ImageJ (version 1.48) as described previously^9^. The average pixel intensity of laser tracks was measured within the locally irradiated area (Idamage), in the nucleoplasm outside the locally irradiated area (Inucleoplasm) and in a region not containing cells in the same field of view (Ibackground). The level of protein accumulation relative to the protein level in the nucleoplasm was calculated as follows: ((Idamage − Ibackground)/(Inucleoplasm − Ibackground) – 1).

### Ultrasoft X-ray irradiation and imaging

The U2OS Flp-In/T-Rex cells stably expressing inducible GFP-tagged ZNF384 were incubated with doxycycline (2 μM) for 2.5 h before being irradiated using the previously described ultrasoft X-ray system^19^. To obtain locally concentrated DSBs, a custom-designed irradiation mask with parallel apertures (2.5-μm wide) was placed between the bottom of the cell culture dishes and the X-ray source. Cells were irradiated for 10 s at 40 mA emission current and 6 KeV acceleration voltage, resulting in approximately 1000 DSBs per irradiated area. Images of cells and of the irradiation mask were collected five min after exposure. For experiments involving immunofluorescence imaging, cells were exposed as described above, without doxycycline preincubation. Five min after irradiation, cells were fixed and immunostained. Wide-field 3D images were acquired using a Leica DMi8 microscope (×63/1.4 NA) and deconvolved using Huygens Professional (version 19.10). Confocal images of immunostained cells were captured using a Leica SP8-X SMD (×63/1.4 NA).

### Proximity ligation assay (PLA)

U2OS AsiSI-ER cells were seeded on 12-mm coverslips and after 24 h treated with 1 µM 4-hydroxytamoxifen (4-OHT, Sigma) for 5 h to induce DSBs. Subsequently, cells were fixed in 4% paraformaldehyde and permeabilized in 0.5% TritonX-100. Primary antibodies rabbit anti-53BP1 (NOVUS Biologicals NB100–304), mouse anti-γH2AX (Millipore clone JBW301), and rabbit anti-ZNF384 (ATLAS antibodies HPA004051) were used to stain selected proteins. Proximity Ligation Assay was performed with Duolink In Situ PLA Probe Anti-Mouse Plus (Sigma) and Anti-Rabbit Minus (Sigma), and with Duolink In Situ Detection Reagents Orange (Sigma) according to the manufacturer’s instructions. Finally, secondary antibodies anti-rabbit coupled to Alexa 488 (Invitrogen) and anti-mouse coupled to Alexa 647 (Invitrogen) were used to stain selected proteins in immunofluorescence. The number and intensity of PLA foci per cell were analyzed by the imaging software ImageJ version 1.48.

### Fluorescence recovery after photobleaching of ZNF384

FRAP of GFP-tagged ZNF384 constructs was performed on a Nikon Ti-E inverted microscope equipped with a spinning-disk scan head CSU-X1 from Yokogawa at a rotation speed of 5000 rpm, a Plan APO ×60/1.4 N.A oil-immersion objective lens, and a sCMOS ORCA Flash 4.0 camera. The fluorescence of EGFP was excited with lasers at 490 nm. Bandpass filters adapted to the fluorophore emission spectra were used for fluorescence detection. Local bleaching within a 4-μm diameter circular area in the cell nucleus was performed using a dedicated single-point scanning head (iLas2 from Roper Scientific) coupled to the epifluorescence backboard of the microscope. Images were collected at 2 images/second. To estimate fluorescence recovery kinetics, the mean fluorescence intensity inside the bleached area was measured by automatic segmentation using a custom-made MATLAB (MathWorks) program R2014b (version 8.4.0.150421) (available upon request). This routine allowed for background subtraction from the intensity measurements and correction for photobleaching due to imaging by dividing the intensity in the bleached area with the one measured for the whole nucleus. The recovery time was the time required to recover half of the fluorescence signal lost upon photobleaching.

### Fluorescence recovery after photobleaching of Ku70

Fluorescence Recovery After Photobleaching (FRAP) of Ku70 was performed on a Zeiss LSM880 confocal setup equipped with a Plan APO ×63/1.2 N.A. water immersion objective. Samples were maintained at 37 °C using a heating chamber. GFP fluorescence was excited at 488 nm and emission was detected at 500–590 nm. DNA damage was induced in a 6 × 2 µm area of the cell nucleus with a pulsed infrared laser set at 800 nm (Mai Tai, Spectra-Physics). Regions of interest of sizes ranging between 1 and 4 µm^2^ located inside the previously irradiated area were bleached using a 488-nm laser. Images of the subsequent fluorescence recovery were collected at 4 frames per second using Zen Black (version 14.0.9.201). After background subtraction, the fluorescence recovery kinetics were obtained by dividing the signal within the bleached area to the one measured in the unbleached part of the damaged region.

### Fluorescence correlation spectroscopy of Ku70

Fluorescence correlation spectroscopy (FCS) of Ku70 was performed on a Zeiss LSM880 confocal microscope equipped with a C-Apo ×40/1.2 N.A water immersion lens. GFP fluorescence was excited with a 488 nm laser and single emitted photons at wavelengths ranging between 500 and 550 nm were detected and counted on the GaAsP spectral detector. The laser power used for FCS measurements was adjusted to minimize photobleaching. FCS acquisition lasted 30 s to reduce the noise in the autocorrelation curves. Samples were maintained at 37 °C using a heating chamber. FCS curves were detrended for slow fluctuations using Fluctuation Analyzer 4G^72^.

### Mathematical models for fitting of the FRAP and FCS data

The diffusion-limited model used to fit the FRAP curves is expressed as,

2 $${frap}\left(t\right)={e}^{\frac{-{{\rm T}}_{D}}{2t}}\left[{I}_{0}\left(\frac{{{\rm T}}_{D}}{2t}\right)+{I}_{1}\left(\frac{{{\rm T}}_{D}}{2t}\right)\right]$$

where T_*D*_ is the characteristic diffusion time within the bleached area and *I*_*0*_ and *I*_*1*_ are modified Bessel functions of the first kind. The parameter T_*D*_ varies with the diffusion coefficient of Ku70 but also, in the case of transient interactions with DNA, it depends on the *K*_*d*_ of this interaction^36^. The reaction-limited model assumes that Ku70 interacts with the DNA breaks according to the following reaction:

3 $${Free}+{BS}\begin{array}{c}\mathop{\to }\limits^{{k}_{{on}}}\\ \mathop{\leftarrow }\limits_{{k}_{{off}}}\end{array}{Bound}$$

with *Free* and *Bound* referring to the binding state of Ku and *BS* to the break site. The mathematical expression of the reaction-limited model is then as follows:

4 $${frap}\left(t\right)=1-\frac{{k^{\prime} }_{{{{{{{\mathrm{on}}}}}}}}}{{k^{\prime} }_{{{{{{{\mathrm{on}}}}}}}}+{k}_{{{{{{{\mathrm{off}}}}}}}}}{e}^{-{k}_{{{{{{{\mathrm{off}}}}}}}}t}$$

where *k’*_on_ is the pseudo-first-order association rate corresponding to the product of the association rate *k*_on_ by the local concentration of break sites [*BS*] and *k*_off_ is the dissociation rate. The one-population model used to fit the correlation curves is expressed as

5 $$G\left(t\right)=\frac{1}{{2}^{3/2}N}{\left(1+\frac{t}{{\rm T}}\right)}^{-1}{\left(1+\frac{t}{{\omega }^{2}{\rm T}}\right)}^{-1/2}$$

where *N* is the number of tagged molecules in the focal volume, τ is the residence time in the focal volume, and ω is the structural parameter of the focal volume, which was fixed to 6. Similarly, the two-population model used to fit the correlation curves is expressed as

6 $$G\left(t\right)=\frac{1}{{2}^{3/2}N}\left[{f}_{1}{\left(1+\frac{t}{{{\rm T}}_{1}}\right)}^{-1}{\left(1+\frac{t}{{\omega }^{2}{{\rm T}}_{1}}\right)}^{-1/2}+(1-{f}_{1}){\left(1+\frac{t}{{{\rm T}}_{2}}\right)}^{-1}{\left(1+\frac{t}{{\omega }^{2}{{\rm T}}_{2}}\right)}^{-1/2}\right]$$

where τ_1_ and τ_2_ are the residence times of the two populations in the focal volume and *f*_*1*_ is the fraction of the molecules belonging to the population displaying a residence time τ_1_.

### Fluorescence three-hybrid assay

Fluorescence three-hybrid/PAR-3H assays were performed as described^27^. Briefly, GFP-tagged proteins were tethered to a genomically integrated LacO array using a LacI-GFP trap in U2OS-2B2 cells^73^ expressing mCherry-PARP1. Cells were sensitized with Hoechst and micro-irradiated with 405 nm light to induce DNA damage. If the GFP-tagged protein of interest is able to bind PAR, PARylated mCherry-PARP1, which is generated at sites of DNA damage, will enrich at the LacO array after DNA damage induction. The mCherry-PARP1 signal intensity at the LacO array was quantified pre and 30, 60, and 120 s post DNA damage induction. The average intensity at the lacO array was normalized to the average intensity of the nucleus and corrected for background signal.

### Immunofluorescence analysis

Cells were either directly fixed with 2% formaldehyde in PBS for 20 min at room temperature (RT), or pre-extracted with 0.5% Triton-X100 (Serva) in PBS on ice for 2 min prior to fixation. Alternatively, cells were fixed, post-extracted with 0.25% Triton-X100 (Serva) in PBS and treated with 100 mM glycine in PBS for 20 min to block unreacted aldehyde groups. Cells were then rinsed with PBS and equilibrated in wash buffer (PBS containing 0.5% BSA). Antibody incubation steps and washes were in wash buffer. Primary antibodies were incubated for 1–2 h at room temperature. Detection was done using goat anti-mouse or goat anti-rabbit Ig coupled to Alexa 488, 555, or 647 (1:1500; Invitrogen Molecular probes) or Cy3-conjugated goat anti-mouse secondary antibody (1:100; Jackson Immuno Research). All antibodies are listed in Supplementary Table 4. Samples were incubated with 0.1 μg/mL 4′, 6-diamidino-2-phenylindole dihydrochloride (DAPI) and mounted in Polymount.

### DSB reporter assay

U2OS 2-6-5 cells stably expressing ER-mCherry-LacR-FokI-DD^41^ were treated for 5 h with 1 μM Shield-1 (Clontech Laboratories UK Ltd) and 1 μM 4-hydroxytamoxifen (4-OHT, Sigma-Aldrich) to induce DSBs.

### Pull-down and co-immunoprecipitation assays

GFP pull-downs were performed on U2OS Flp-In/T-Rex cells expressing GFP-NLS, GFP-ZNF384 or the indicated GFP-tagged ZNF384 mutants and on HeLa and GFP-Ku80-expressing HeLa cells, while untransfected U2OS cells were used for co-immunoprecipitation assays. Cells were lysed in EBC buffer (50 mM Tris, pH 7.5, 150 mM NaCl, 0.5% NP-40, 2 mM MgCl_2_, protease inhibitor cocktail tablets) with 500 units benzonase. Samples were incubated for 1 h at 4 °C under constant mixing. 50 μL input sample was collected in a separate tube and mixed with 2× Laemmli buffer. The cleared lysates were subjected to GFP pull-down with GFP-Trap beads (Chromotek) or immunoprecipitation using a specific antibody (or corresponding IgG control) that was conjugated to Protein G-coupled agarose beads (Millipore 16–201). The beads were then washed six times with EBC buffer and boiled in 2× Laemmli buffer along with the input samples. Samples were subjected to western blot analysis.

### Sample preparation and mass spectrometry

For mass spectrometry, U2OS Flp-In/T-Rex cells expressing GFP-NLS and GFP-ZNF384 were pelleted and lysed in EBC-1 buffer (50 mM Tris, pH 7.5, 150 mM NaCl, 0.5% NP-40, 2 mM MgCl_2_, protease inhibitor cocktail tablets) with 500 units benzonase. Samples were incubated for 1 h at 4 °C under constant mixing followed by high speed centrifugation for 10 min at 4 °C. Protein concentration was measured by Qubit in the cleared lysates, equalized and transferred to tubes containing GFP-Trap beads (Chromotek). After 90 min of incubation at 4 °C under rotating condition, the beads were washed four times with EBC-2 buffer (50 mM Tris pH 7.5, 150 mM NaCl, 1 mM EDTA, and protease inhibitor cocktail tablets) and three times with 50 mM ammonium bicarbonate followed by overnight digestion using 2.5 μg trypsin at 37 °C under constant shaking. Digestion was terminated with 1% trifluoroacetic acid and centrifuged for 5 min at high speed to precipitate insoluble fractions. Consequently, C18 cartridges were prepared by washing two times with acetonitrile followed by two times with 0,1% acetic acid. Peptides were loaded on the cartridge, while bound peptides were washed two times with 0.1% acetic acid and eluted with 1 mL 80% acetonitrile/0.1% acetic acid and lyophilized.

Mass spectrometry was performed essentially as previously described^74^. Samples were analyzed on a Q-Exactive Orbitrap mass spectrometer (Thermo Scientific, Germany) coupled to an EASY-nanoLC 1000 system (Proxeon, Odense, Denmark). Digested peptides were separated using a 15-cm fused silica capillary (ID: 75 μm, OD: 375 μm, Polymicro Technologies, California, USA) in-house packed with 1.9-μm C18-AQ beads (Reprospher-DE, Pur, Dr. Maisch, Ammerburch-Entringen, Germany). Peptides were separated by liquid chromatography using a gradient from 2 to 95% acetonitrile with 0.1% formic acid at a flow rate of 200 nl/minute for 65 min. The mass spectrometer was operated in positive-ion mode at 2.8 kV with the capillary heated to 250 °C, and in a data-dependent acquisition (DDA) mode with a top seven method. Full-scan MS spectra were obtained with a resolution of 70,000, a target value of 3 × 10^6^, and a scan range from 400 to 2000 *m/z*. Maximum Injection Time (IT) was set to 50 ms. Higher-Collisional Dissociation (HCD) tandem mass spectra (MS/MS) were recorded with a resolution of 35,000, a maximum IT of 20 ms, a target value of 1 × 10^5^ and a normalized collision energy of 25%. The precursor ion masses selected for MS/MS analysis were subsequently dynamically excluded from MS/MS analysis for 60 s. Precursor ions with a charge state of 1 and greater than 6 were excluded from triggering MS/MS events. Three replicates were included per condition with two technical repeats each.

### Mass-spectrometry data analysis

Raw mass-spectrometry files were analyzed with MaxQuant software (v1.5.5.1) as described^75^ with the following modifications from default settings: the maximum number of mis-cleavages by trypsin/p was set to 3, label-free quantification (LFQ) was enabled disabling the Fast LFQ feature. The Match-between-runs feature was enabled with a match time window of 0.7 min and an alignment time window of 20 min. We performed the search against an in silico digested UniProt reference proteome for Homo sapiens (June 8, 2020). Analysis output from MaxQuant was further processed in the Perseus computational platform (version 1.5.5.3)^75^. Proteins identified as common contaminants, only identified by site and reverse peptide, were filtered out, and then all the LFQ intensities were log2 transformed. Different biological repeats of each condition were grouped and only protein groups identified in all three biological replicates in at least one condition were included for further analysis. Missing values were imputed using Perseus software by normally distributed values with a 1.8 downshift (log2) and a randomized 0.3 width (log2) considering total matrix values. Volcano plots were generated, and Student’s *t* tests were performed to compare the different conditions. Spreadsheets from the statistical analysis output from Perseus were further processed in Microsoft Excel for comprehensive visualization and analysis of the data (Supplementary Table 1).

### Western blot analysis

Cells were lysed in 2× Laemmli buffer and proteins were separated by sodium dodecyl sulfate-polyacrylamide gel electrophoresis (SDS-PAGE) using 4–12% pre-cast polyacrylamide gels (BioRad or Invitrogen) and MOPS running buffer (Invitrogen). Next, proteins were transferred onto nitrocellulose membranes (Millipore). Protein expression was analyzed by immunoblotting with the indicated primary antibodies (Supplementary Table 3) and secondary CF680 goat anti-rabbit or CF770 goat anti-mouse Ig antibodies (1:5000, Biotium). Membranes were scanned and analyzed using an Odyssey Infrared Imaging System (Licor; V3.0) and Image Studio Lite (version 5.2). Uncropped blots are provided in the source data file.

### Chromatin fractionation

Chromatin fractionation was performed using a previously published protocol^76^ with few modifications. Briefly, 100.000–150.000 cells were grown per 6-cm dish for 24 h and then transfected with siRNAs. Next, the cells were treated with 500 µM phleomycin for 1 h, washed three times with PBS, and incubated in NETN extraction buffer (100 mM NaCl, 1 mM EDTA, 20 mM Tris-Cl pH 8, 0.5% NP-40 + proteasome inhibitors). After 15 min of incubation on ice, samples were taken for the chromatin-unbound fraction and mixed with the same amount of 2× Laemmli buffer. Cells were washed with PBS, lysed, and incubated in Laemmli buffer with benzonase for 15 min to obtain the chromatin-bound fraction. Samples were heated for 7 min at 80 °C and subjected to western blot analysis.

### MBP-based protein purifications

For MBP-based purification, cultures of *Escherichia Coli* BL21-CodonPlus (DE3)-RIL cells containing pET-His6-MBP,pET-His6-MBP-ZNF384, pET-His6-MBP-C2H2, pET-His6-MBP-N-terminus, and pET-His6-MBP-C-terminus plasmids were grown to an OD_600_ of 0.3 absorbance units. To start induction of protein expression, 0.3 mM IPTG was added to the culture followed by incubation overnight at 20 °C. After centrifugation, cell pellets were frozen and stored at −80 °C. For protein purification, cell pellets were lysed in 5 ml B-per™ Bacterial Protein Extraction Reagent (ThermoFisher Scientific) supplemented with protease inhibitors (Sigma) and 15 kU rLysozyme (Merck) until the lysates were clear. The viscosity of the lysate was decreased by the addition of 125 units benzonase or sonication. The lysate was centrifuged for a centrifuge for 10 min at 21,000 × *g* at 4 °C in a table centrifuge 21,000 × *g*. For the ZNF384 FL, N-terminus and C- terminus fragments, the supernatant was loaded on a column packed with 0.75 ml Amylose Resin High Flow (NEB) installed in ÄKTA pure protein purification system (Cytiva). The column was washed with buffer A (20 mM Tris pH 7.4, 200 mM NaCl, 1 mM EDTA, 10 mM β-ME) and the proteins were eluted with buffer B (buffer A + 10 mM maltose). For the ZNF384 C2H2 fragment, two purification steps were performed. First, the supernatant was loaded on HiTrap SP HP Strong Cation Exchange column (Cytiva) and proteins were eluted using a linear gradient from 50 to 1000 mM NaCl in 20 mM Tris pH 7.4, 10 mM β-ME collecting 2 ml fractions. Second, the fractions containing C2H2 were loaded onto the Amylose column, followed by washing and elution with buffer A and B, respectively.

### Biotinylated DNA substrates

Biotinylated DNA substrates (Supplementary Table 5) were used at a concentration of 1 pmol/µl. dsDNA substrates were made by annealing complementary oligo’s (Supplementary Tables 2 and 4) in reaction buffer (10 mM Tris pH 7.5, 150 mM KCl, 5 mM MgCl_2_, 0.25% Tween-20, 3.5 mM DTT, 5% glycerol). Annealing was done in a PCR machine heating for 2 min to 95 °C, then gradually cooling over a period of 45 min to 25 °C.

### DNA pull-down assay

DNA-binding reactions were done at 4 °C in 40 µl reaction buffer containing 0.1% BSA, 10 pmol biotinylated DNA substrate (Supplementary Table 5), and ~50 fmol of purified MBP or the different MBP-tagged ZNF384 proteins. After 30 min, the reaction buffer containing 10 µl Dynabeads M-280 streptavidin suspension and 0.1% BSA was added and samples were incubated for 15 min at 4 °C. After this incubation, beads were washed three times using 200 µl reaction buffer and loaded on 4–12% polyacrylamide Bis-Tris gel. After electrophoresis, proteins were blotted onto PVDF membranes for one hour at 50 Volt. Membranes were incubated at room temperature for 1 h with mouse monoclonal anti-MBP antibody (NEB), followed by 1 h incubation with goat anti-mouse HRP antibody (Bethyl Laboratories), and imaged by AI680 imager (GE) with ECL. For Ku70/Ku80 DNA-binding reactions to 3′-overhang DNA, His-MBP-ZNF384 or His-MBP, 100 fmol Ku70/Ku80 heterodimer, and 10 pmol 3′-overhang biotinylated oligo were incubated in 40 µl reaction buffer (10 mM Tris pH 7.5, 150 mM KCl, 5 mM MgCl_2_, 0.25% Tween-20, 3.5 mM DTT, 5% glycerol) with 0.1% BSA for 30 min at 4 °C. After 30 min, reaction buffer containing 10 µl Dynabeads M-280 streptavidin suspension and 0.1% BSA was added and samples were incubated for 30 min at 4 °C. After this incubation, beads were washed three times with 200 µl reaction buffer, Laemmli sample buffer was added and samples were incubated for 10 min at 95 °C. For separation by electrophoresis, samples were loaded on Bolt 4–12% polyacrylamide Bis-Tris Mini Protein gel (ThermoFisher). After electrophoresis, western blotting was performed and the blots were stained using mouse anti-MBP (NEB) and rabbit anti-Ku80 (Santa Cruz) as primary antibodies and goat anti-Rabbit^CF680^ and goat anti-Mouse^CF770^ (Biotium) as secondary antibodies for detection with an Odyssey Infrared Imaging System (Licor; V3.0).

### In vitro pull-down assay

Protein reactions and washing steps were all done at room temperature. In total, 15 µl Dynabeads™ M-280 Sheep anti-Mouse IgG (Thermo Scientific) were incubated with 2 µg mouse anti-Ku80 antibody (Santa Cruz) in 40 µl PBS containing 0.1% BSA for 30 min. Beads were collected using a magnetic rack. After discarding the supernatant, beads were washed with 200 µl PBS containing 0.1% BSA and incubated for 30 min with 6 pmol of recombinant His-Ku70/Ku80 (Sino Biological) in 10 µl reaction buffer (10 mM Tris pH 7.5, 150 mM KCl, 5 mM MgCl_2_, 0.25% Tween-20, 3.5 mM DTT, 5% glycerol). Beads were collected and the supernatant was discarded. Beads were then washed with 200 µl reaction buffer and 10 µl of 0.2% BSA was added to the reaction buffer. After 15 min, 10 µl reaction buffer with 1 pmol of purified His-MBP-tagged protein was added and samples were incubated for 30 min. Beads were collected and the supernatant was removed. Beads were subsequently washed four times with 200 µl reaction buffer. Samples were heated in Laemmli sample buffer for 10 min at 95 °C. For electrophoresis, samples were loaded on Bolt 4–12% polyacrylamide Bis-Tris Mini Protein gel (ThermoFisher). After electrophoresis, Western blotting was performed and the blots were stained using mouse anti-MBP (NEB) and rabbit anti-Ku80 (Santa Cruz) as primary antibodies and goat anti-Rabbit^CF680^ and goat anti-Mouse^CF770^ (Biotium) as secondary antibodies for detection with an Odyssey Infrared Imaging System (Licor; V3.0).

### Biolayer interferometry (BLI) measurements

BLI measurements were done on an OctedRed System (Sartorius), shaking the assay plate (1000 rpm) at 298 K. All steps were performed in BLI buffer (10 mM Tris PH 7.5, 150 mM KCl, 5 mM MgCl_2_, 0.05% (v/v) Tween-20, 0.1% (w/v) BSA, 1 mM DTT). Biotinylated DNA substrates (Supplementary Table 5) were immobilized on streptavidin sensors pre-equilibrated in BLI buffer, after which a washout in BLI buffer was done. Then, 400 nM of purified MBP or the different MBP-tagged ZNF384 proteins was used to measure the association of the analyte. The resulting data were processed using the ForteBio Data Analysis software (version 7.1.0.38).

### Quantitative fluorescence in situ hybridization (FISH) of telomeres

Telomere FISH was based on a previously published protocol^57^. Briefly, HCT116 cells were harvested following 2 h of colcemid (Sigma) incubation. After hypotonic swelling, cells were fixed in methanol/acetic acid, dropped on slides, and dried at 37 °C overnight. The next day slides were treated with RNaseA (R4642; Sigma), pepsin (P7000; Sigma) at pH 2, followed by formaldehyde fixation, washes in PBS, dehydration in ethanol, and air drying. Hybridization mixture containing 70% formamide, 0.3 μg/ml Cy3-conjugated (C3TA2)3 peptide nucleic acid (PNA) probe in 10 mM Tris (pH 7.5) was added to the slide, after which a coverslip was added followed by DNA denaturation for 1.5 min at 80 °C. After hybridization for 2 h at room temperature, slides were washed with 70% formamide/10 mM Tris/pH 7.2, and 0.05 M Tris/0.15 M NaCl containing 0.05% Tween-20. Slides were stained with DAPI, dehydrated with ethanol, air dried, and mounted in Aquapolymount (Polysciences). Images were acquired on a Zeiss AxioImager M2 wide-field fluorescence microscope with 63x PLAN APO (1.4 NA) oil-immersion objectives (Zeiss). Integrated density and area of single telomeres were measured with ImageJ (version 1.48) by using Threshold, Polygon, and Analyze Particles functions, subsequently. While obtaining images we noticed a variation of telomere signals between metaphases and less so within one metaphase spread. Therefore, after subtraction of background values, the average integrated density per telomere of each metaphase was calculated and plotted.

### DR-GFP and EJ5-GFP reporter assays

U2OS cells containing either a stably integrated copy of the DR-GFP or EJ5-GFP reporter were used to measure the repair of I-*Sce*I-induced DSBs by HR or NHEJ^47,66^. Briefly, DR-GFP U2OS cells or EJ5-GFP U2OS cells treated with siRNA for 48 h were co-transfected with an mCherry expression vector and the I-*Sce*I expression vector pCBASceI^66^. Forty-eight hours later, the percentage of GFP-positive cells among the mCherry-positive cells was determined by FACS on a BD LSRII flow cytometer (BD Bioscience) using FACSDiva software version 5.0.3. An example of the gating strategy can be found in Supplementary Fig. S15I. Quantifications were performed with FACSDiva™ (BD Biosciences).

### Random plasmid integration assay

U2OS cells were seeded (day 1) and transfected with siRNAs the following day (day 2). Later at day 2, the cells were transfected with 2 μg gel-purified *Bam*HI/*Eco*RI-linearized pEGFP-C1 plasmid. The cells were subsequently transfected twice with siRNAs at 24 and 36 h after the first transfection (day 3 and day 4, respectively). On day 5, cells were collected, counted, seeded, and grown in medium without or with 0.5 mg/mL G418. The transfection efficiency was determined on the same day by FACS analysis using GFP fluorescence as a measure. The cells were incubated at 37 °C to allow colony formation and medium was refreshed on days 8 and 12. On day 15, the cells were washed with 0.9% NaCl and stained with methylene blue (2.5 g/L in 5% ethanol, Sigma-Aldrich). Colonies of more than 50 cells were scored. Random plasmid integration efficiency was scored as the number of G418-resistant colonies normalized by the plating efficiency, which was determined by the number of colonies formed on plates without G418 and corrected for the transfection efficiency.

### Analysis of repair junctions in the GC92 reporter

Sequence analysis of repair junctions in the GC92 reporter was performed as described^54^. Briefly, GC92 fibroblasts were first transfected with siRNAs and 48 h later with the I-*Sce*I expression vector pCBASce^66^. 48 h later, genomic DNA was extracted using phenol:chloroform:isoamyl alcohol (25:24:1 v/v, Invitrogen). PCR was performed on the genomic DNA using the CMV1 and CD4int primers (Supplementary Table 3) to amplify repair junctions. PCR products were cloned into pGEM-T easy vector (Promega). Colony PCR was performed using M13 primers (Supplementary Table 3) on individual bacterial colonies to amplify repair junctions, which were subjected to Sanger sequencing using the M13 FW primer (Supplementary Table 3). Sequences were analyzed using a custom Sanger sequence analyzer, as described previously^44^.

### Cell survival assays

VH10-SV40 cells were transfected with siRNAs, trypsinized, seeded at low density, and exposed to IR. For HeLa Flp-In/T-Rex, cDNAs were expressed by adding Dox for 24 h after siRNA transfection. U2OS cells were seeded at low densities and exposed to increasing doses of olaparib. After 7 days, the cells were washed with 0.9% NaCl and stained with methylene blue (2.5 g/L in 5% ethanol, Sigma-Aldrich). Colonies of more than 20 cells were scored.

### Cell cycle profiling

Cells were fixed in 70% ethanol, followed by DNA staining with 50 µg/mL propidium iodide in the presence of RNaseA (0.1 mg/mL; Sigma). Cell acquisition and quantification were performed on a BD LSRII flow cytometer (BD Bioscience) using FACSDiva software version 5.0.3.

### Statistics and reproducibility

Results were confirmed in multiple cell lines or by using complementary approaches. All experiments yielding micrographs, pull-down experiments, and western blot analysis were performed independently at least twice, but often three times. The MS experiments were performed in triplicate. Statistical analysis was carried out using the two-tailed Student’s *t* test (*P* < 0.05). Boxplots were generated using R (version 4.0.5) and R Studio (version 1.4.1106).

### Reporting summary

Further information on research design is available in the Nature Research Reporting Summary linked to this article.

## Supplementary information

Supplementary Information

Reporting Summary

## Data Availability

The data that support this work are available from the corresponding author upon reasonable request. The mass-spectrometry proteomics data generated in this study and shown in Fig. 1d and Supplementary Table 1 have been deposited to the ProteomeXchange Consortium via the PRIDE partner repository (https://www.ebi.ac.uk/pride/)^77^. Access can be obtained with the dataset identifier PDX020417. In addition, publicly available reference datasets for Homo sapiens (June 8, 2020) were used to search against an in Silico-digested UniProt reference proteome. Source data are provided with this paper.

## Source data

Source Data

## Code availability

The code used in this work is available on Github at https://github.com/sehuet/Singh-image-processing.
