# Supplementary material for: Zinc finger protein ZNF384 is an adaptor of Ku to DNA during classical non-homologous end-joining
Source: Nat Commun. 2021 Nov 12;12:6560. doi: 10.1038/s41467-021-26691-0 (PMC8589989; doi:10.1038/s41467-021-26691-0)
Supplement: Supplementary file 1 — Supplementary Information [file 41467_2021_26691_MOESM1_ESM.pdf]

Supplementary Information

Zinc finger protein ZNF384 is an adaptor of Ku to DNA during classical non-homologous end-joining

Jenny Kaur Singh, Rebecca Smith, Magdalena B. Rother, Anton J.L. de Groot, Wouter W. Wiegant, Kees Vreeken, Ostiane D'Augustin, Robbert Q. Kim, Haibin Qian, Przemek M. Krawcyk, Román González-Prieto, Alfred C.O. Vertegaal, Meindert Lamers, Sébastien Huet[3,5] and Haico van Attikum

This file contains:

- Supplementary Figure 1
- Supplementary Figure 2
- Supplementary Figure 3
- Supplementary Figure 4
- Supplementary Figure 5
- Supplementary Figure 6
- Supplementary Figure 7
- Supplementary Figure 8
- Supplementary Figure 9
- Supplementary Figure 10
- Supplementary Figure 11
- Supplementary Figure 12
- Supplementary Figure 13
- Supplementary Figure 14
- Supplementary Figure 14
- Supplementary Figure 15
- Supplementary Table 1
- Supplementary Table 2
- Supplementary Table 3
- Supplementary Table 4
- Supplementary Table 5
- Supplementary References

Singh et al_Supplementary Fig.1

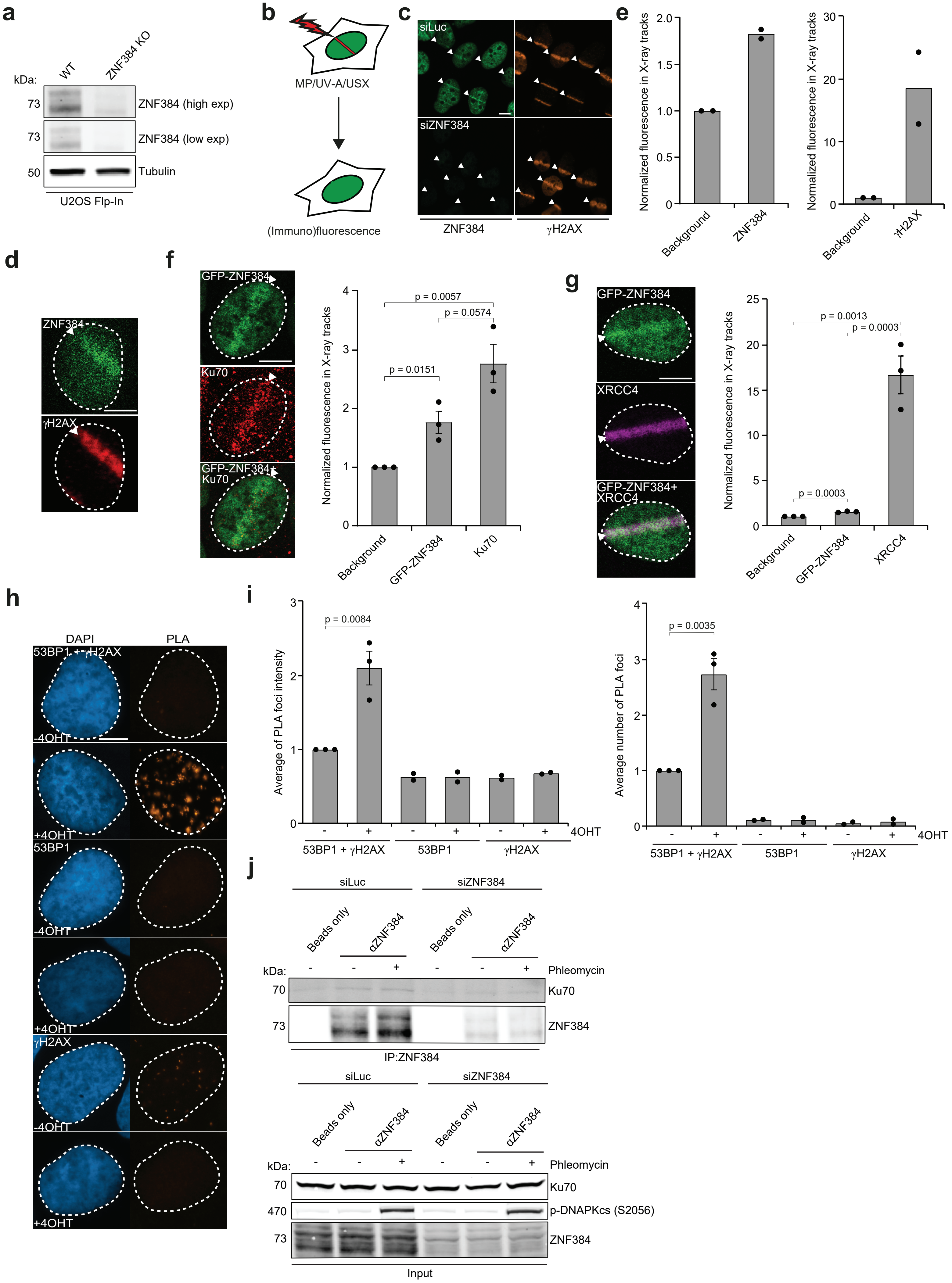

Supplementary Fig. 1 – related to Fig. 1. ZNF384 is recruited to DNA damage sites (A) Western blot analysis of ZNF384 expression in cells from Figure 1A. Tubulin is a loading control. Data shown represent 2 independent experiments. (B) Schematic representation of the laser micro-irradiation approach to study protein accumulation at sites of DNA damage. An 800 nm multiphoton (MP) laser, 365 nm UV-A laser or Ultra-soft X-ray (USX) irradiation was used. (C) Representative images of two independent experiments of the accumulation of ZNF384 at γH2AX-marked 365 nm UV-A tracks in cells transfected with the indicated siRNAs. Cells were fixed and immunostained 10 minutes after laser micro-irradiation. White triangles indicate irradiated regions. (D) Accumulation of endogenous ZNF384 at γH2AX-marked DNA damage inflicted by USX irradiation in U2OS Flp-In/T-Rex cells expressing doxycycline (dox)-inducible GFP-ZNF384. Cells were fixed and immunostained 5 minutes after USX irradiation. White triangles indicate irradiated regions. (E) Quantification of ZNF384 and γH2AX accumulation in cells from D. The mean of at least 40 cells acquired in 2 independent experiments is shown. Data were normalized to the nuclear background outside the irradiated area, which was set to 1. (F) Accumulation of GFP-ZNF384 at sites of Ku70-marked DNA damage inflicted by USX irradiation in U2OS Flp-In/T-Rex cells expressing doxycycline (dox)-inducible GFP-ZNF384. Cells were fixed and immunostained 5 minutes after laser micro-irradiation. White triangles indicate irradiated regions. (left panel). Quantification of ZNF384 and Ku70 is presented as the ±SEM of >45 cells acquired in 3 independent experiments. Data were normalized to the nuclear background outside the irradiated area, which was set to 1 (right panel). P-values were calculated using the two-tailed unpaired Student's *t* test. (G) As in F, but for GFP-ZNF384 and XRCC4. Quantification of ZNF384 and XRCC4 is presented as the ±SEM of 60 cells acquired in 3 independent experiments. P-values were calculated using the two-tailed unpaired Student's *t* test, assuming unequal variances. (H) PLA of 53BP1 and ZNF384 and γH2AX in *Asi*SI–ER-U2OS cells treated with 4-OHT for DSB induction. PLA foci were scored after 5 hours of DSB induction. (I) Quantification of H. PLA foci formation and foci intensity from >200 cells acquired in 2-3 independent experiments are shown. Statistical significance was calculated with the two-tailed Student's *t* test, assuming unequal variances. (J) Immunoprecipitation (IP) of endogenous ZNF384 from 500μM Phleomycin treated U2OS cells transfected with the indicated siRNAs. Control IP contained beads only. Blots were probed for ZNF384 and Ku70. Data shown represent 2 independent experiments. Scale bar 5 μm, except for C in which scale bar is 10 μm. Source data are provided as Source Data file.

Singh et al_Supplementary Fig. 2

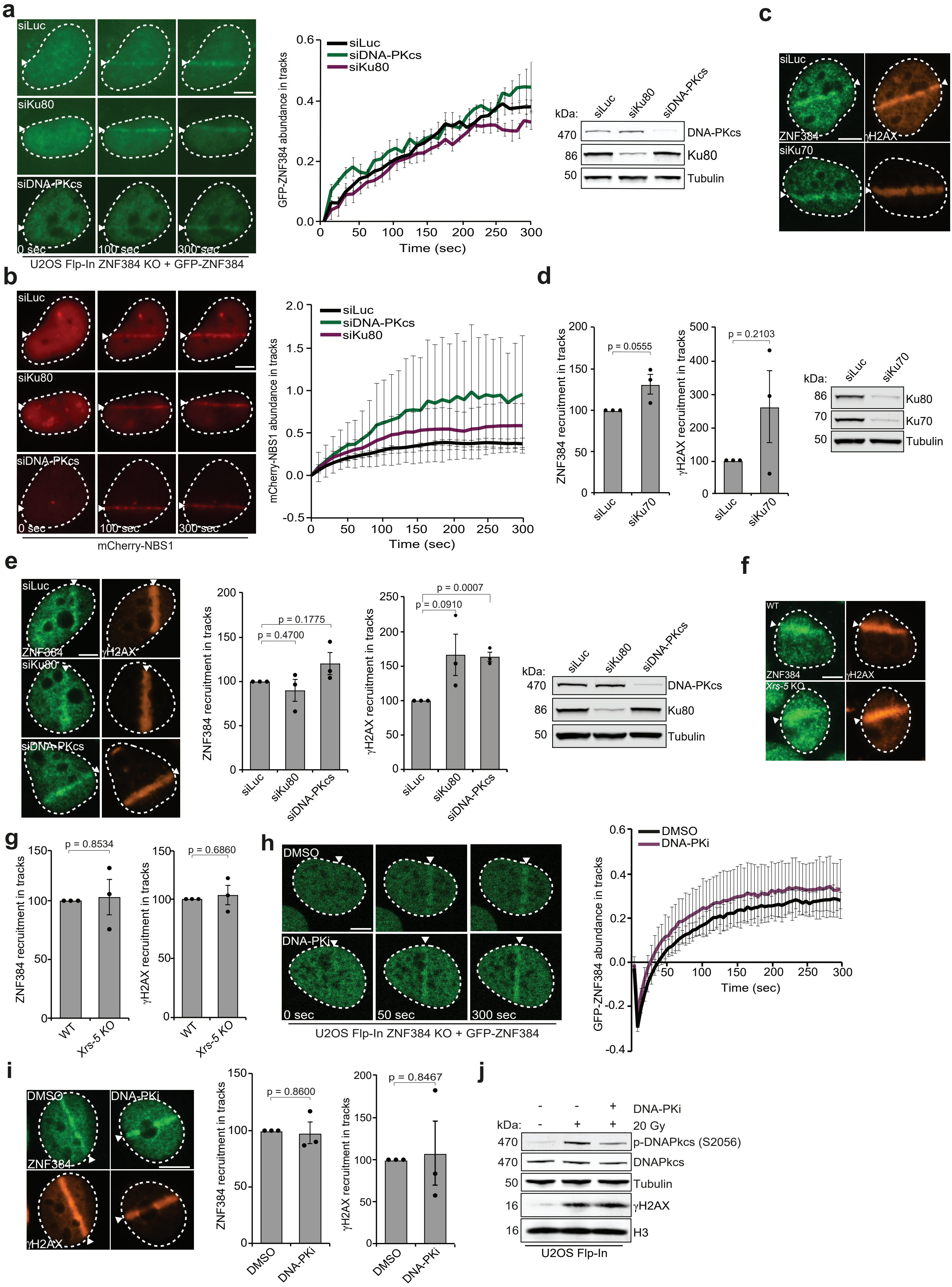

Supplementary Fig. 2 – related to Fig. 1. ZNF384 is recruited to DNA damage sites independent of Ku70/Ku80 and DNA-PKcs (A) Recruitment of GFP-ZNF384 to 365 nm UV-A tracks in BrdU-sensitized U2OS Flp-In/T-Rex *ZNF384* KO cells. White triangles indicate irradiated regions (left panel). Quantification of the data is plotted on a time scale as relative abundance in tracks. The graph represents the mean ±SEM of >40 cells acquired in 3 independent experiments (middle panel). Western blot analysis of Ku80 and DNA-Pkcs expression. Tubulin is a loading control. Data shown represent 2 independent experiments (right panel). (B) As in A, except for mCherry-NBS1. (C) Accumulation of endogenous ZNF384 at 365 nm UV-A tracks in BrdU-sensitized U2OS cells following transfection with siLuc and siKu70. Cells were fixed and immunostained 10 minutes after laser micro-irradiation. White triangles indicate irradiated regions. γH2AX was used as a DNA damage marker. (D) Quantification of endogenous ZNF384 and γH2AX levels in laser tracks in cells from D is presented as the mean ±SEM of >180 cells acquired in 3 independent experiments (left panel). Western blot analysis of Ku70 and K80 expression in cells. Tubulin is a loading control. Data shown represent 2 independent experiments (right panel). (E) Accumulation of endogenous ZNF384 at 365 nm UV-A tracks in BrdU-sensitized U2OS cells following transfection with siLuc, siKu80 and siDNA-PKcs. Cells were fixed and immunostained 10 minutes after laser micro-irradiation. White triangles indicate irradiated regions. γH2AX was used as a DNA damage marker (left panel). Quantification of endogenous ZNF384 and γH2AX levels in laser tracks is presented as the mean ±SEM of >180 cells acquired in 3 independent experiments (middle panel). Western blot analysis of Ku80 and DNA-Pkcs expression. Tubulin is a loading control. Data shown represent 2 independent experiments (right panel). (F) Accumulation of endogenous ZNF384 at 365 nm UV-A tracks in BrdU-sensitized wildtype (WT) and *xrs-5* KO hamster cells. Cells were fixed and immunostained 10 minutes after laser micro-irradiation. White triangles indicate irradiated regions. γH2AX was used as a DNA damage marker. (G) Quantification of F. Endogenous ZNF384 and γH2AX levels in laser tracks are presented as the mean ±SEM of >150 cells acquired in 3 independent experiments. (H) Kinetics of GFP-ZNF384 recruitment to 800 nm laser tracks in U2OS Flp-In/T-Rex *ZNF384* KO cells treated for 1 hour with DNA-PKcs inhibitor prior to laser micro-irradiation. White triangles indicate irradiated regions (left panel). Quantification of GFP-ZNF384 in laser tracks is presented as the mean ±SD from 2 independent experiments (right panel). (I) Accumulation of endogenous ZNF384 at 365 nm UV-A tracks in BrdU-sensitized U2OS cells treated for 1 hour with DNA-PKcs inhibitor prior to laser micro-irradiation. Cells were fixed and immunostained 10 minutes after laser micro-irradiation. White triangles indicate irradiated regions. γH2AX was used as a DNA damage marker (left panel). Quantification of endogenous ZNF384 and γH2AX levels in laser tracks is presented as the mean ±SEM of >180 cells acquired in 3 independent

experiments (right panel). Statistical significance was calculated with the two-tailed Student's *t* test, assuming unequal variances. Scale bar 5 µm. (J) Western blot analysis of p-DNA-PKcs (S2056) levels in IR- and DNA-PKcs inhibitor-treated cells used in I. Cells were treated with DNA-PKi 1 hour before IR exposure. Blots were probed for p-DNA-PKcs (S2056), DNA-PKcs, γH2AX. Tubulin and H3 are loading controls. Source data are provided as Source Data file.

Singh et al_Supplementary Fig.3

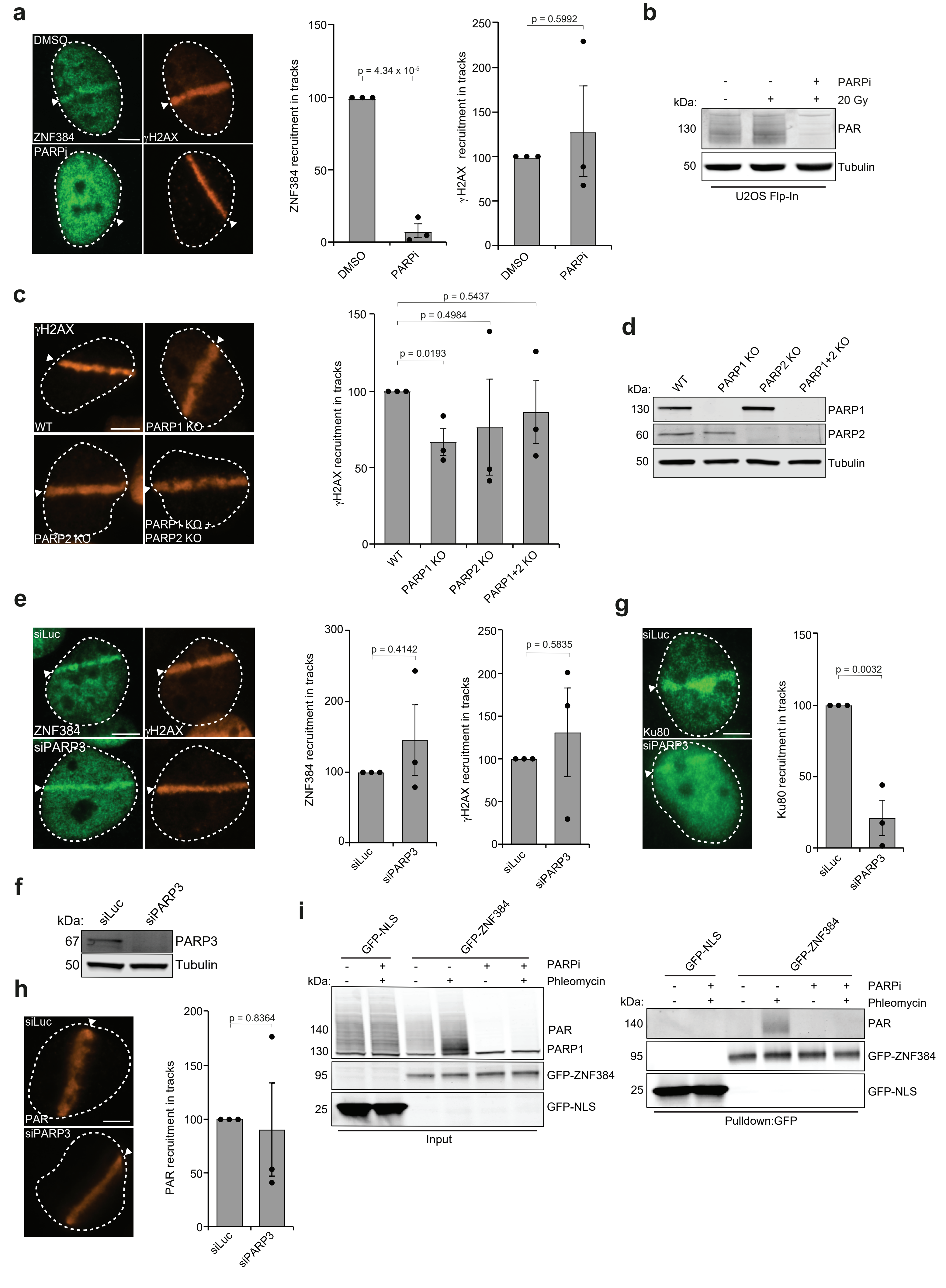

Supplementary Fig. 3 – related to Fig. 1. ZNF384 is recruited to DNA damage sites via PARP1/PAR (A) Accumulation of endogenous ZNF384 at 365 nm UV-A tracks in BrdU-sensitized U2OS cells treated for 1 hour with PARPi prior to laser micro-irradiation. Cells were fixed and immunostained 10 minutes after laser micro-irradiation. White triangles indicate irradiated regions. γH2AX was used as a damage marker (left panel). Quantification of endogenous ZNF384 and γH2AX levels in laser tracks is presented as the mean ±SEM of >200 cells acquired in 3 independent experiments (right panel). (B) Western blot analysis of PAR levels in IR treated and PARPi treated cells used in Figure 2A and Figure S3A. Cells were treated with PARPi 1hr before the IR treatment. Tubulin is a loading control. Data shown represent 2 independent experiments. (C) γH2AX accumulation at 365 nm UV-A tracks 10 minutes after DNA damage induction in BrdU-sensitized wildtype, PARP1 KO, PARP2 KO and PARP1/PARP2 KO U2OS cells. White triangles indicate irradiated regions (left panel). The mean ±SEM of >180 cells from 3 independent experiments is shown (right panel). (D) Western blot analysis of PARP1 and PARP2 expression in cells from Figure 2B and Figure S3C. Tubulin is a loading control. Data shown represent 2 independent experiments. (E) Accumulation of endogenous ZNF384 at 365 nm UV-A tracks in BrdU-sensitized U2OS cells transfected with the indicated siRNAs. Cells were fixed and immunostained 10 minutes after laser micro-irradiation. White triangles indicate irradiated regions. γH2AX was used as a damage marker (left panel). Quantification of endogenous ZNF384 and γH2AX levels in laser tracks is presented as the mean ±SEM of > 200 cells acquired in 3 independent experiments (right panel). (F) Western blot analysis of PARP3 expression in cells from Figure S3E. Tubulin is a loading control. Data shown represent 2 independent experiments. (G) Accumulation of endogenous Ku80 at 365 nm UV-A tracks in BrdU-sensitized U2OS cells transfected with the indicated siRNAs. Cells were fixed and immunostained 10 minutes after laser micro-irradiation. White triangles indicate irradiated regions (left panel). Quantification of endogenous Ku80 levels in laser tracks is presented as the mean ±SEM of >120 cells acquired in 3 independent experiments (right panel). (H) As in G, except for PAR. (I) Pull-downs of GFP-ZNF384 and GFP-NLS under denaturing conditions in untreated and phleomycin (Phleo)-treated U2OS Flp-In/T-Rex cells expressing doxycycline (dox)-inducible GFP-ZNF384 or GFP-NLS. Cells were also treated with PARPi (Olaparib) for 1 hour prior to the phleomycin treatment. Blots were probed for PAR, PARP1 and GFP. Data shown represent 2 independent experiments. Statistical significance was calculated with the two-tailed Student's *t* test, assuming unequal variances. Scale bar 5 μm. Source data are provided as Source Data file.

Singh et al_Supplementary Fig. 4

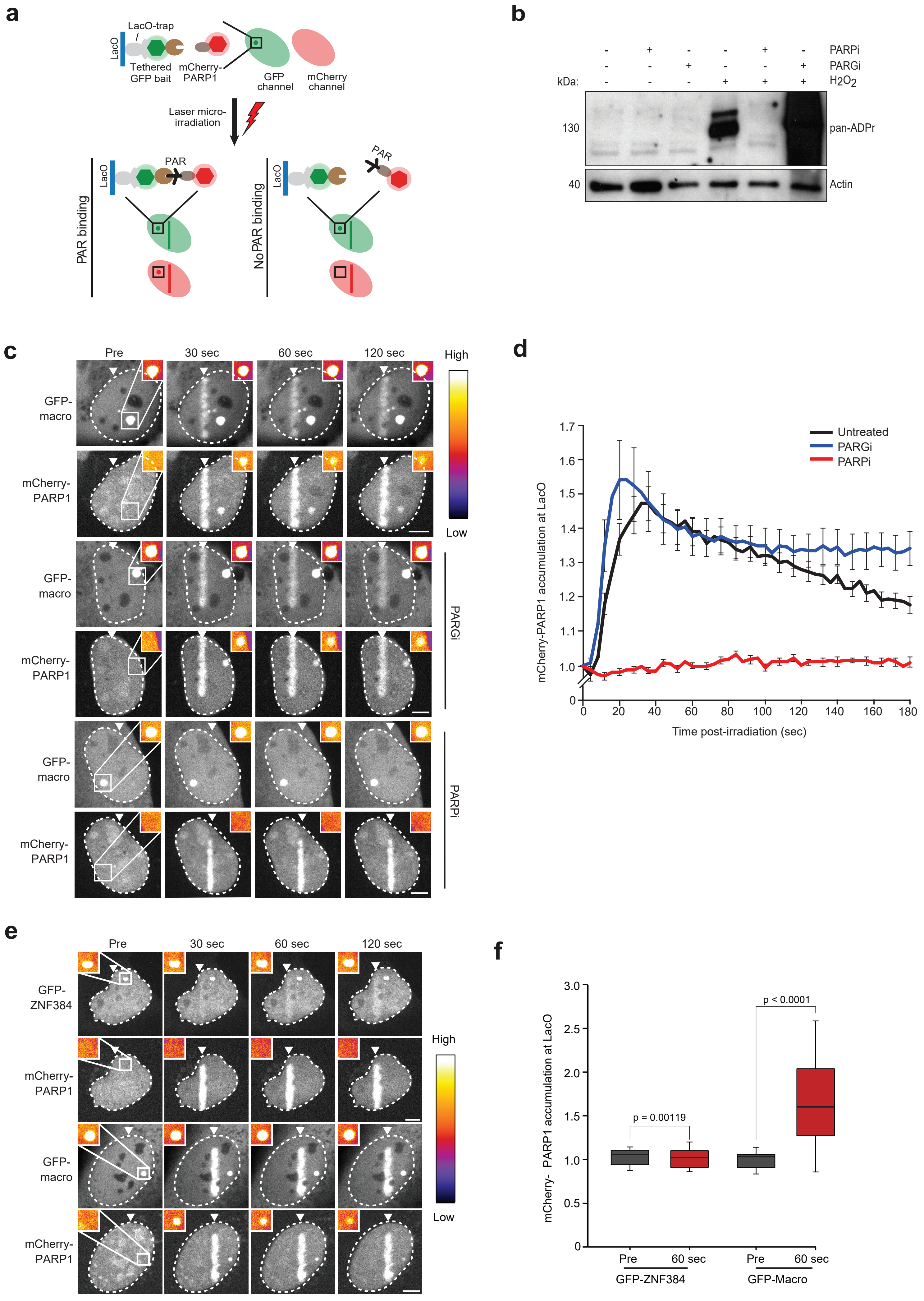

Supplementary Fig. 4 – related to Fig. 2. ZNF384 is recruited to DNA damage sites via PARP1/PAR dependent chromatin unfolding (A) Schematic of the fluorescence three-hybrid assay (F3H). Prior to laser irradiation, a GFP-tagged bait protein which is tethered to a genomically integrated LacO array through a LacI-GFP binding protein (LacO-trap) does not show enrichment of mCherry-PARP1 at the LacO array. After laser micro-irradiation, mCherry-PARP1 diffuses away from the site of damage in a PARylated state. If the tethered GFP-tagged bait protein can interact with PARylated PARP1 (bottom left), mCherry-PARP1 will enrich at the LacO array. If the tethered protein of interest does not interact with the tethered bait protein (bottom right), there is no enrichment of mCherry-PARP1 at the LacO array. (B) Western blot analysis of PAR levels in H_2O_2 and PARPi or PARGi treated cells used in Figure S4D. Cells were treated with 2 mM H_2O_2 for 10 min to induce PARylation. Blot was probed with panADPr. Actin is a loading control. Blots are representative of 3 independent replicates. (C) Fluorescence images of *in situ* PAR binding three-hybrid assays. LacI-GFP-macroH2A1.1 (GFP-macro) was tethered to a LacO array in Hoechst-sensitized cells co-expressing mCherry-PARP1 treated for 1 hour with PARPi or PARGi prior to 405 nm laser micro-irradiation. White triangles indicate irradiated regions. Inset shows the magnified LacO array. Insets are pseudocolored according to the look-up table displayed on the right. Scale bar 5 μm. (D) The mean intensity of mCherry signal at the LacO array from 19-20 cells was quantified pre damage and 60 seconds post damage. Data shows mean intensity of the mCherry signal at the LacO array ±SEM. Cells were treated for 1 hour with PARPi or PARGi prior to micro-irradiation. (E) Fluorescence images of *in situ* PAR binding three-hybrid assays. GFP-ZNF384 or GFP-macro was tethered to a LacO array in Hoechst-sensitized cells co-expressing mCherry-PARP1 prior to 405 nm laser micro-irradiation. White triangles indicate irradiated regions. Inset shows the magnified LacO array. Insets are pseudocolored according to the look-up table displayed on the right. (F) The mean intensity of mCherry signal at the LacO array from 12-15 cells was quantified pre damage and 60 seconds post damage. The boxplot limits correspond to the 25th and 75th percentiles and the center line in the box indicates the median value. The whiskers extend 1.5 times the interquartile range. P-values were calculated using the two-tailed Student's *t* test, assuming unequal variances. Scale bar 5 μm. Source data are provided as Source Data file.

Singh et al_Supplementary Fig. 5

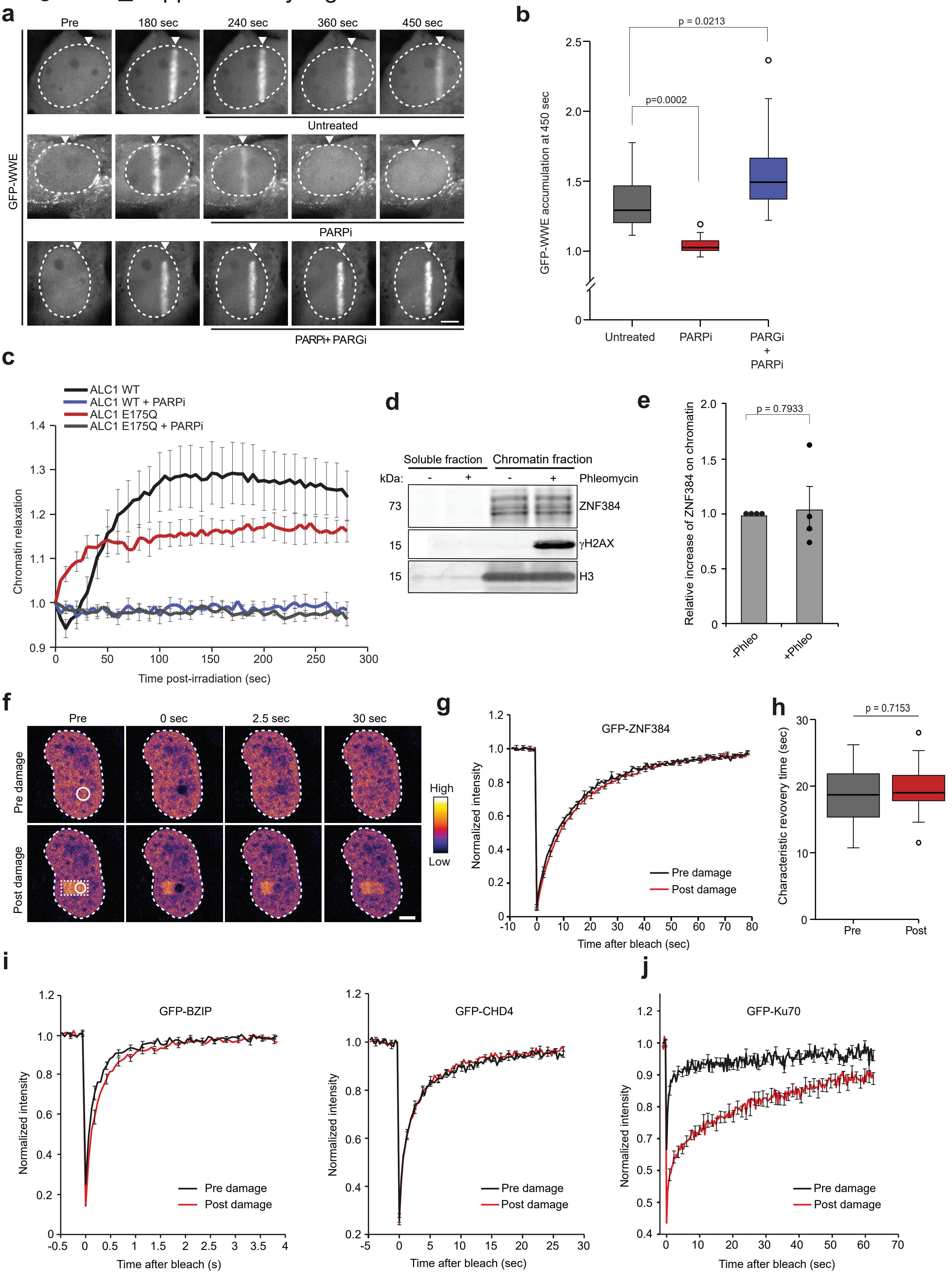

Supplementary Fig. 5 – related to Fig. 2. ZNF384 is recruited to DNA damage sites via PARP1/PAR dependent chromatin unfolding. (A) Confocal images showing accumulation of GFP-WWE at sites of 405 nm laser micro-irradiation in Hoechst-sensitized U2OS cells. Cells were left untreated or treated with PARPi or PARPi and PARGi 240 seconds after DNA damage induction. White triangles indicate irradiated regions. Scale bar 5 µm. (B) Boxplot showing the accumulation of GFP-WWE at 450 seconds post irradiation from cells in A. Boxplots show the first, median and third quartiles from a representative of 3 independent replicates. The whiskers extend 1.5 times the interquartile range. P-values were calculated using the two-tailed unpaired Student's *t* test, assuming unequal variances. (C) Chromatin relaxation was measured for each of the overexpression conditions from Figure 2G. The mean ±SEM from 13-16 cells from a representative of 3 independent experiments is shown. (D) Western blot analysis of the indicated proteins in soluble and chromatin fractions from untreated and phleomycin treated U2OS cells. H3 is the loading control. γH2AX is a DNA damage marker. A representative experiment is shown. (E) Quantification of ZNF384 levels on chromatin in cells from D. H3 is a loading control, which was used for normalization of ZNF384 levels on chromatin. The mean ±SEM from 4 independent experiments is shown. P-values were calculated using the Student's *t* test, assuming unequal variances. (F) Representative images of Hoechst-sensitized U2OS Flp-In/T-Rex *ZNF384* KO cells in which FRAP measurements were performed to assess the local turnover of GFP-ZNF384 at DNA damage sites. DNA damage was induced in the region indicated with a dashed line using 405 laser micro-irradiation. Subsequent FRAP was induced in a subarea within the damaged region, as indicated with an unbroken line. Images are pseudocolored according to the look-up table displayed on the right. Scale bar 4 µm. (G) Normalized FRAP curves from F. Data show mean ±SEM from 14 cells per condition from a representative of 3 independent replicates (H) Boxplot showing the characteristic recovery times estimated from the FRAP curves shown in F. The boxplot limits correspond to the 25th and 75th percentiles and the center line in the box indicates the median value. The whiskers extend 1.5 times the interquartile range. P-values were calculated using the two-tailed paired Student's *t* test. (I) Normalized FRAP curves of the GFP-tagged DNA binding domain BZIP from the transcription factor C-EBPa and DNA damage induction in U2OS cells (left panel). Normalized FRAP curves of GFP-tagged CHD4 and DNA damage induction in U2OS cells (right panel). (J) Normalized FRAP curves of GFP-Ku70 and DNA damage induction in RPE1-hTERT cells. The mean ±SD is shown from 14 cells per condition from a representative of 2 independent replicates. Source data are provided as Source Data file.

Singh et al_Supplementary Fig. 6

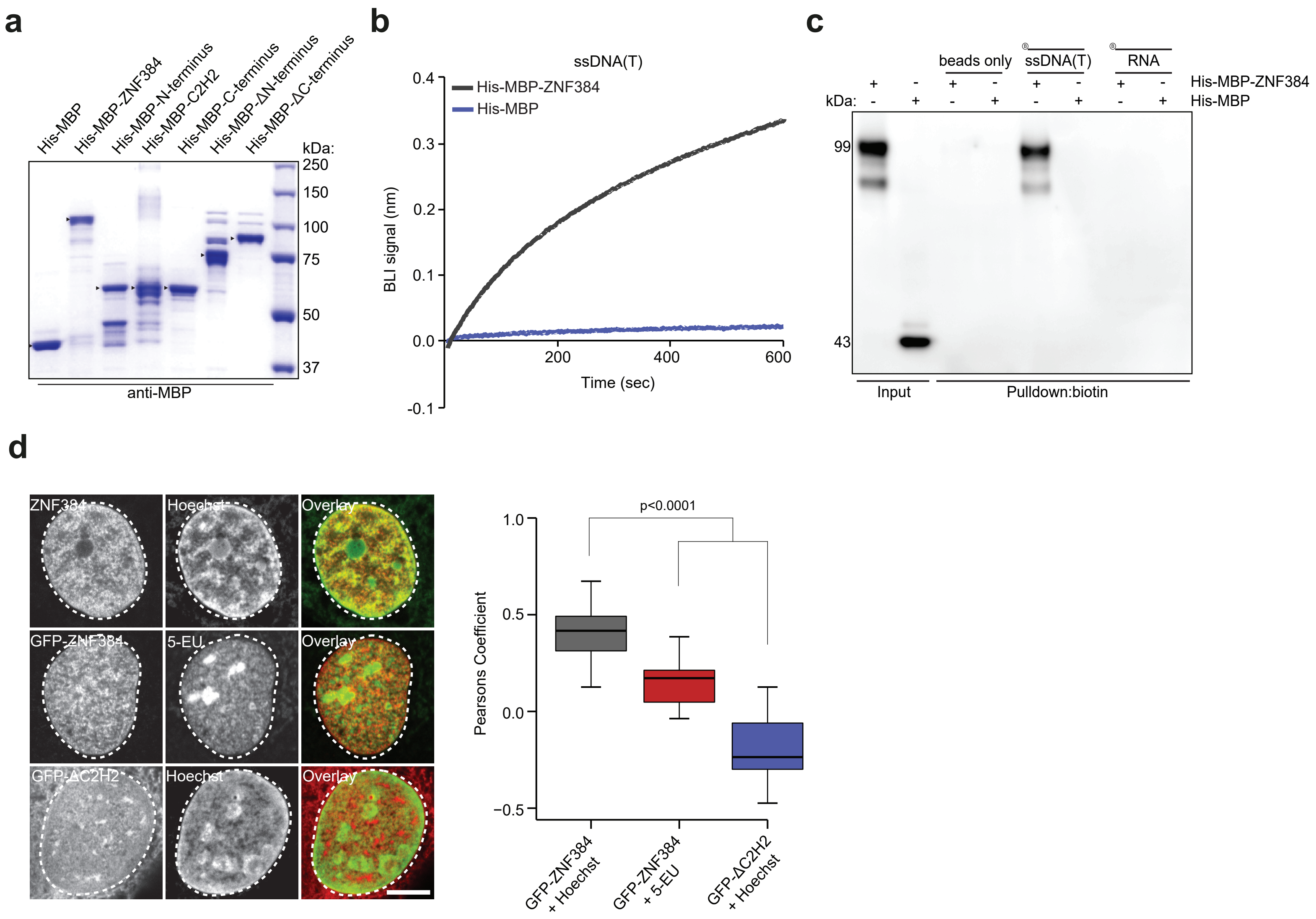

Supplementary Fig. 6 – related to Fig. 3. ZNF384 is recruited to sites of DNA damage in manner dependent on its ability to bind DNA (A) SDS-PAGE analysis of the indicated purified His-MBP-tagged proteins. Data shown represent 2 independent experiments. (B) DNA binding by His-MBP-ZNF384 and His-MBP to biotinylated T-rich ssDNA (ssDNA(T)) by BLI. (C) Pulldowns of the indicated biotinylated DNA or RNA substrates in the presence of His-MBP-ZNF384 or His-MBP. Blots were probed for MBP. Data shown represent 2 independent experiments. (D) Representative images of U2OS Flp-In/T-Rex *ZNF384* KO cells expressing either GFP-tagged full-length ZNF384 or ∆C2H2. Cells were co-stained with Hoechst or 5-Ethynyl Uridine (5-EU) to assess their colocalization with DNA or RNA (left panel). The levels of colocalization between ZNF384 or ∆C2H2 and DNA or RNA were assessed using the Pearson coefficient (right panel). The mean ±SD is shown from >23 cells per condition. The boxplot limits correspond to the 25th and 75th percentiles and the center line in the box indicates the median value. The whiskers extend 1.5 times the interquartile range. For GFP-ZNF384 + Hoechst vs GFP-ZNF384 + 5-EU, p= 7.1156×10^{-7}. For GFP-ZNF384 + Hoechst vs GFP-∆C2H2 + Hoechst, p= 4.027×10^{-12}. P-values were calculated using the two-tailed unpaired Student's *t* test, assuming unequal variances. Source data are provided as Source Data file.

Singh et al_Supplementary Fig. 7

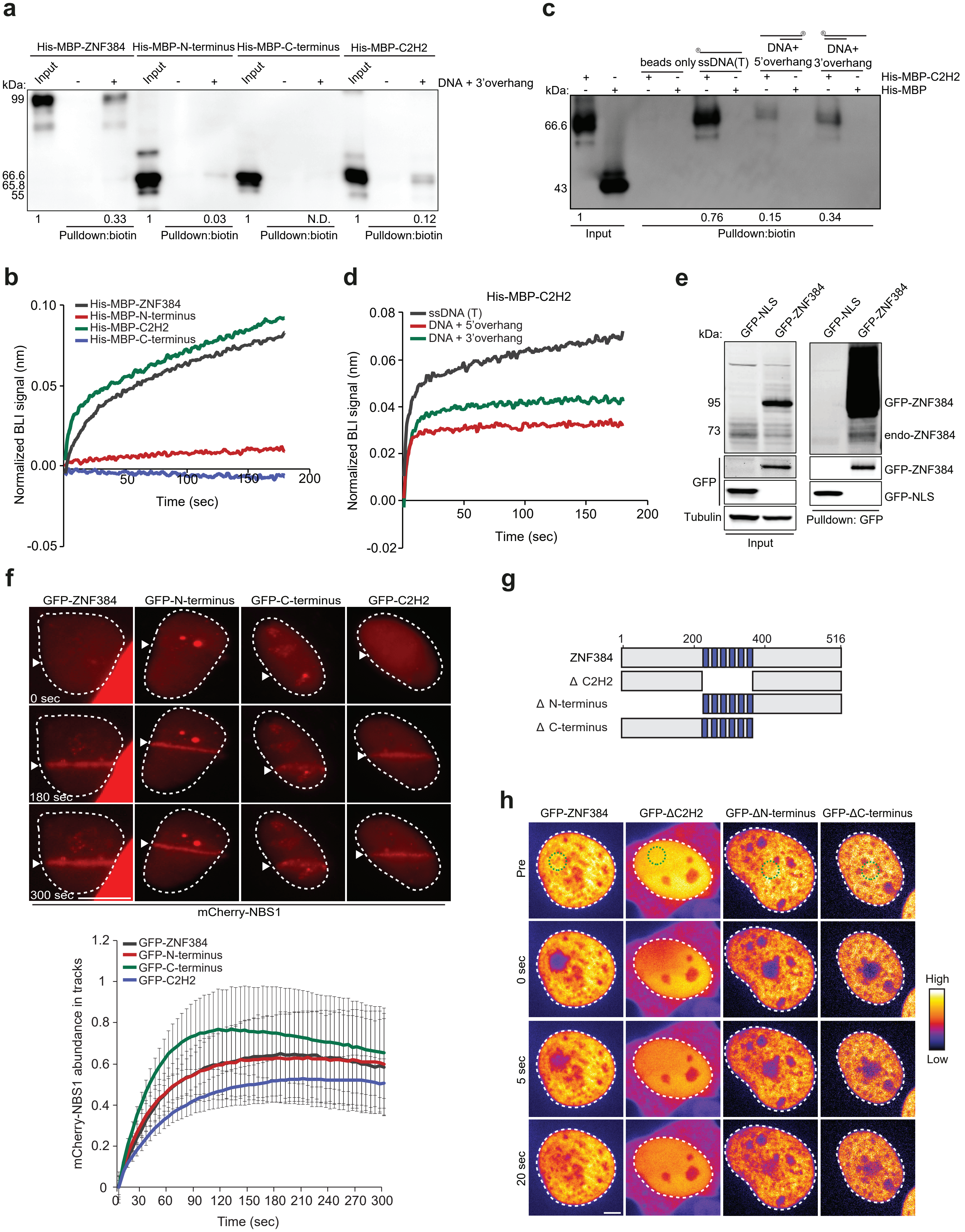

Supplementary Fig. 7 – related to Fig. 3. ZNF384 is recruited to sites of DNA damage via its C2H2 domain (A) DNA pulldowns of biotinylated DNA with a 3'overhang in the presence of the indicated ZNF384 proteins. Blots were probed for MBP. For each protein, input signals were used for normalization of the pulldown signals. The mean of 2 independent experiments is indicated below the blot. A representative experiment is shown. N.D. = not detectable. (B) DNA binding by the indicated ZNF384 proteins to DNA with a 3'overhang as measured by BLI. Quantification of the data is plotted on a time scale and normalized to His-MBP alone. (C) DNA pulldowns of biotinylated T-rich ssDNA (ssDNA(T)), biotinylated DNA with a 5'overhang or biotinylated DNA with a 3'overhang in the presence of His-MBP-C2H2 or His-MBP (control). Blots were probed for MBP. His-MBP-C2H2 pulldown signals were normalized to that in the input, which was set to 1. The mean from 3 independent experiments is indicated below the blot. His-MBP was not detectable in pulldowns. A representative experiment is shown. (D) DNA binding of His-MBP-C2H2 to biotinylated T-rich ssDNA (ssDNA(T)), biotinylated DNA with a 5'overhang or biotinylated DNA with a 3'overhang as measured by BLI. Quantification of the data is plotted on a time scale and normalized to His-MBP (control). (E) Pulldowns of the indicated GFP-fusion proteins in U2OS Flp-In/T-Rex cells. Blots were probed for GFP, ZNF384 and Tubulin. Data shown represent 2 independent experiments. (F) Live cell imaging of mCherry-NBS1 to 365 nm UV-A tracks in BrdU-sensitized *ZNF384* KO U2OS Flp-In/T-Rex cells. mCherry-NBS1, which was co-expressed with GFP-ZNF384 proteins, served as a DNA damage marker for Figure 3D. Representative images are shown. White triangles indicate irradiated regions. Scale bars 10 µm (upper panel). Quantification of the data is shown as mean ±SEM from 30-40 cells (lower panel). (G) Schematic representation of full-length ZNF384 and three deletion mutants (∆C2H2, ∆N-terminus and ∆C-terminus). (H) Representative images of cells from Figure 3E in which the FRAP measurements are performed to assess the local dynamics of the indicated GFP-ZNF384 constructs. FRAP area is indicated with a dashed line. Images are pseudocolored according to the look-up table displayed on the bottom. Scale bar: 4 µm. Source data are provided as Source Data file.

Singh et al_Supplementary Fig. 8

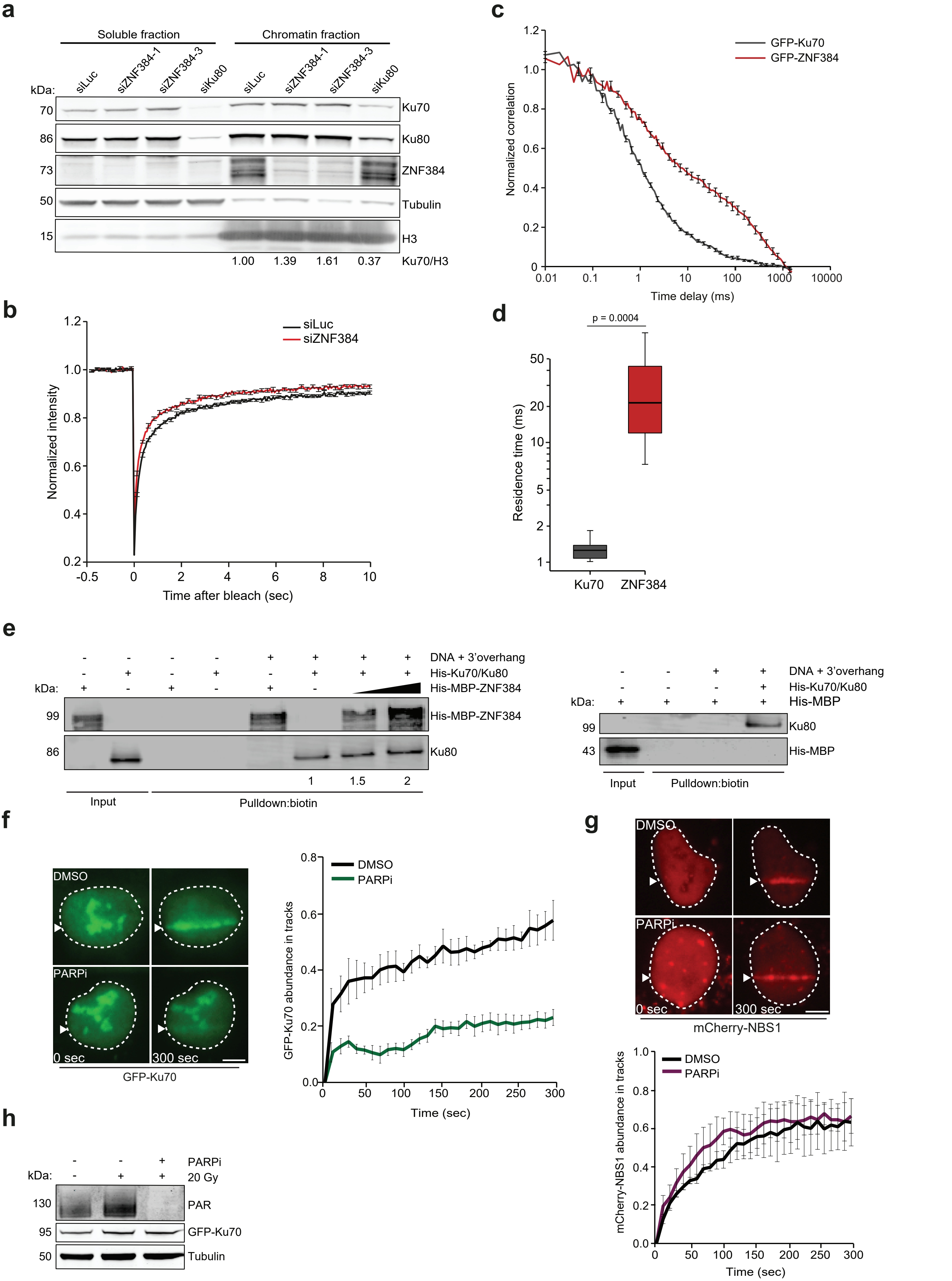

Supplementary Fig. 8 – related to Fig. 4. ZNF384 modulates the loading of Ku70/Ku80 at DNA damage sites (A) Western blot analysis of the indicated proteins in soluble and chromatin fractions from U2OS cells. Blots were probed for the indicated proteins. H3 and Tubulin are loading controls. A representative experiment is shown. Ku70 levels in chromatin were first normalized to H3 levels. Ku70/H3 ratios were then normalized to that for siLuc, which was set to 1. Quantifications are shown as the mean from 2 independent experiments. (B) Normalized FRAP curves of GFP-Ku70 in RPE-hTERT cells transfected with the indicated siRNAs in the absence of DNA damage. The mean ±SD is shown from 20-21 cells per condition. (C) Normalized FCS autocorrelation curves of GFP-Ku70 in RPE1-hTERT cells and GFP-ZNF384 in U2OS cells in the absence of DNA damage. The mean ±SEM is shown from 10-16 cells analyzed per condition. (D) Boxplot showing the residence times of GFP-Ku70 and GFP-ZNF384 in RPE1-hTERT and U2OS cells, respectively, as measured by FCS. 10-15 cells were analyzed per condition. The boxplot limits correspond to the 25th and 75th percentiles and the center line in the box indicates the median value. The whiskers extend 1.5 times the interquartile range. P-values were calculated using the two-tailed unpaired Student's *t* test, assuming unequal variances. (E) DNA pulldowns of biotinylated DNA with a 3'overhang in the presence of His-Ku70/Ku80, His-MBP-ZNF384 or His-MBP (control) alone or His-Ku70/Ku80 in combination with His-MBP-ZNF384 or His-MBP (control). Blots were probed for MBP and Ku80. Ku80 pulldown signals were normalized to that in the pulldown lacking His-MBP-ZNF384, which was set to 1. The mean from 4 independent experiments is indicated below the blot. His-MBP was not detectable in pulldowns. (F) GFP-Ku70 recruitment to 365 nm UV-A tracks in cells treated with PARPi for 1 hour before laser micro-irradiation. White triangles indicate irradiated regions (left panel). Quantification of the data is presented as the mean ±SEM of >100 cells acquired in 3 independent experiments (right panel). (G) As in F, except for mCherry-NBS1. (H) Western blot analysis of PAR levels, which likely reflect PARP1 autoPARylation, in phleomycin- and PARPi-treated cells from F and G. Cells were treated with PARPi for 1 hour before IR treatment. Blots were probed for PAR, GFP and Tubulin. Tubulin is a loading control. Data shown represent 2 independent experiments. Statistical significance was calculated using the two-tailed unpaired Student's *t* test, assuming unequal variances. Scale bar 5 µm. Source data are provided as Source Data file.

Singh et al_Supplementary Fig. 9

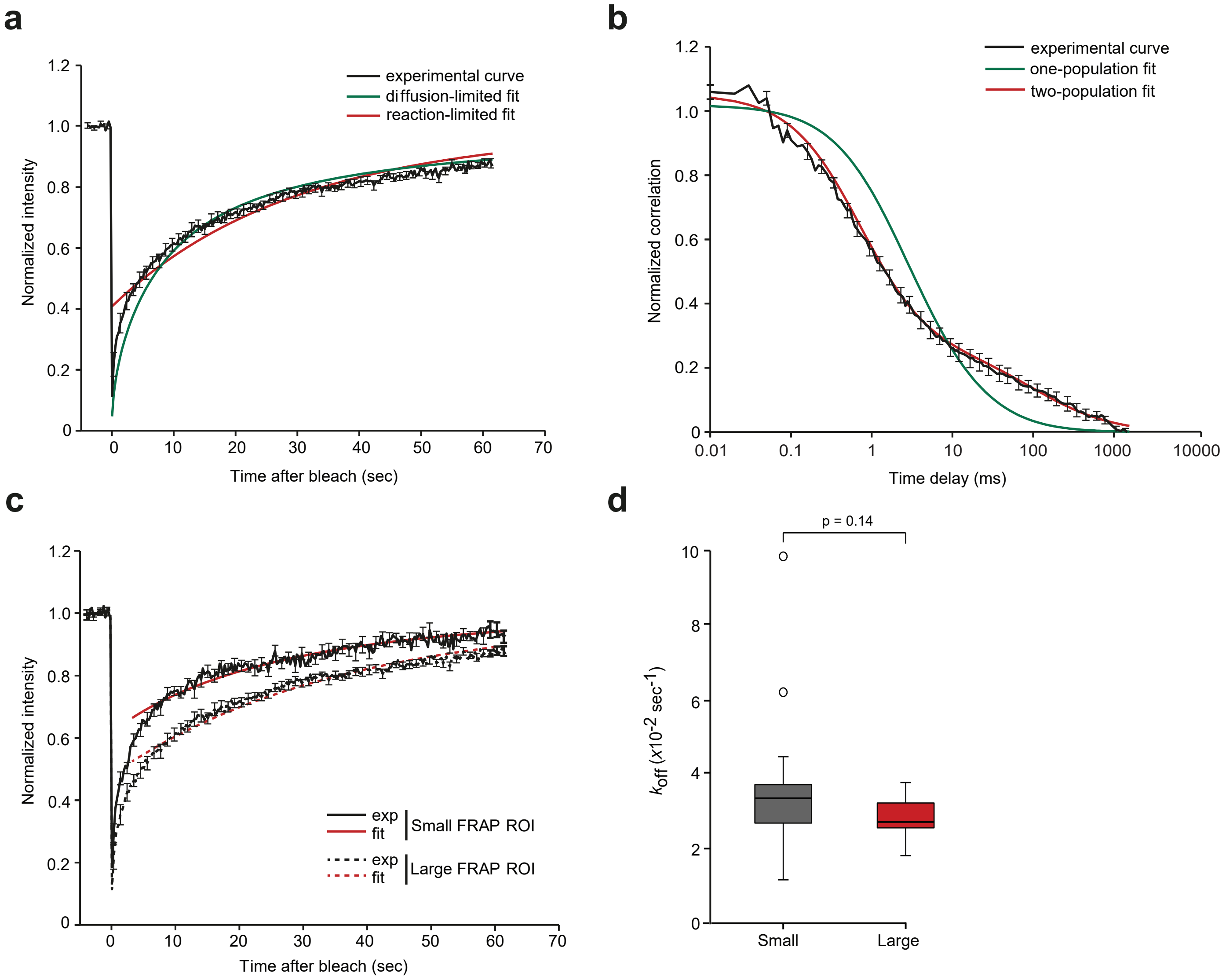

Supplementary Fig. 9 – related to Fig. 4. ZNF384 facilitates the recruitment rather than the retention of Ku70/Ku80 at sites of DNA damage (A) FRAP curves of GFP-Ku70 at the DNA lesions fitted with two alternative models: i) a diffusion-limited model which assumes that Ku interacts only very transiently with DNA at the sites of damage, and ii) a reaction-limited model which is valid if the fluorescence recovery is limited by the long lifetime of the interaction between Ku and the DNA lesions [1]. The mean ±SEM is shown from 15 cells from a representative of 3 independent experiments is shown. (B) FCS curves of GFP-Ku70 at the DNA lesions fitted with models assuming either one or two population dynamics. Based on the 2-population fit, the fast population displays a characteristic diffusion time of 590±70 µseconds within the confocal volume. Given that the molecular weight of the GFP-tagged Ku70/Ku80 complex (180 kDa) is ~6 times larger than GFP only, and that GFP shows a diffusion time of approximately 300 µseconds when diffusing through the nucleus [2], the Stockes-Einstein equation predicts that the diffusion time on an unbound Ku70/Ku80 complex should be equal to $300*6^{1/3}=550$ µseconds. Therefore, it seems reasonable to assume that the fast population of Ku seen by FCS corresponds to freely diffusing Ku complexes. The mean ±SEM is shown from 10 cells from a representative of 3 independent experiments. (C) FRAP curves of GFP-Ku70 at the DNA lesions obtained for two different bleaching area and fitted with a reaction-limited model for the timepoints at more than 3 seconds post photobleaching. The mean ±SEM is shown from 15 cells from a representative of 3 independent experiments is shown. (D) k_{off} parameters estimated from the reaction-limited model for the two different sizes of bleached area from C. The mean ±SEM is shown from 15 cells from a representative of 3 independent experiments is shown. The boxplot limits correspond to the 25th and 75th percentiles and the center line in the box indicates the median value. The whiskers extend 1.5 times the interquartile range. P-values were calculated using the two-tailed unpaired Student's *t* test, assuming unequal variances. Source data are provided as Source Data file.

Singh et al_Supplementary Fig. 10

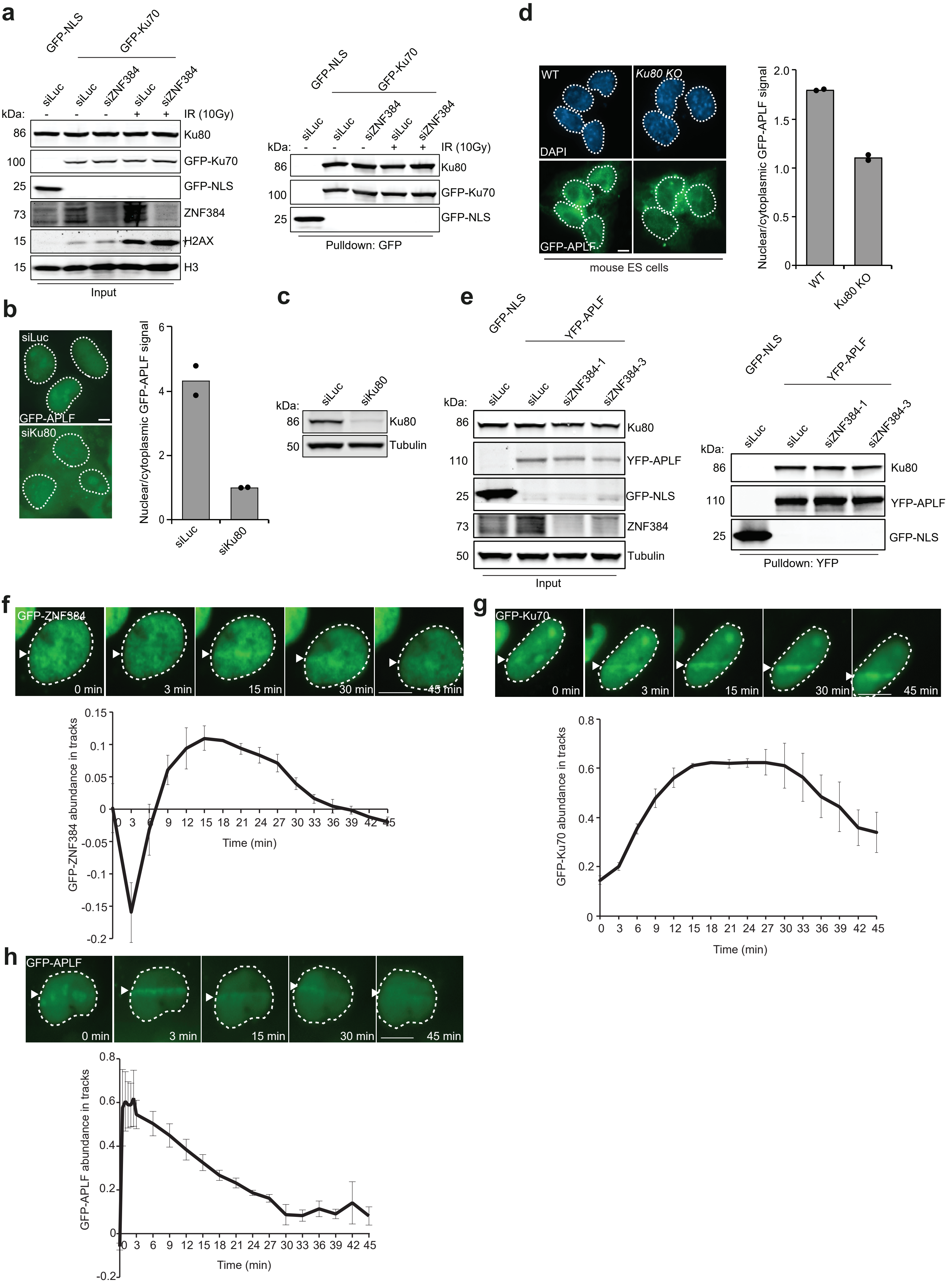

Supplementary Fig. 10 – related to Fig. 5 and 6. ZNF384 is dispensable for cNHEJ complex stability (A) Pull-downs of the indicated GFP fusion proteins in IR treated RPE-hTERT cells transfected with indicated siRNAs. Blots were probed for GFP, Ku80, ZNF384, γH2AX and H3. Data shown represent 2 independent experiments. (B) Nuclear localization of GFP-APLF in U2OS Flp-In/T-Rex cells expressing doxycycline (dox)-inducible GFP-APLF transfected with the indicated siRNAs (left panel). Quantification of the data is presented as the mean of >200 cells acquired in 2 independent experiments (right panel). (C) Western blot analysis of Ku80 in cells from B. Tubulin is a loading control. Data shown represent 2 independent experiments. (D) Nuclear localization of GFP-APLF in wildtype and *Ku80*$^{-/-}$ mouse embryonic stem (ES) cells (left panel). Quantification of the data is presented as the mean of >60 cells acquired in 2 independent experiments (right panel). (E) Pull-downs of the indicated GFP fusion proteins transfected with indicated siRNAs in U2OS cells. Blots were probed for GFP, Ku80, ZNF384 and tubulin. Data shown represent 2 independent experiments. (F) Kinetics of GFP-ZNF384 recruitment to 365 nm UV-A tracks in BrdU-sensitized U2OS Flp-In/T-Rex *ZNF384* KO cells. White triangles indicate irradiated regions (left panel). Quantification of GFP-ZNF384 is presented as the mean ±SD from >120 cells acquired in 2 independent experiments (right panel). (G) Kinetics of GFP-Ku70 recruitment to 365 nm UV-A tracks in BrdU-sensitized RPE1-hTERT cells. White triangles indicate irradiated regions. (left panel). Quantification of GFP-Ku70 is presented as the mean ±SD from >120 cells acquired in 2 independent experiments (right panel). (H) Kinetics of GFP-APLF recruitment to 365 nm UV-A tracks in BrdU-sensitized U2OS Flp-In/T-Rex GFP-APLF cells. White triangles indicate irradiated regions (left panel). Quantification of GFP-APLF is presented as the mean ±SD from <120 cells acquired in 2 independent experiments (right panel). Statistical significance was calculated with the two-tailed unpaired Student's *t* test, assuming unequal variances. Scale bar 5 µm. Source data are provided as Source Data file.

Singh et al_Supplementary Fig. 11

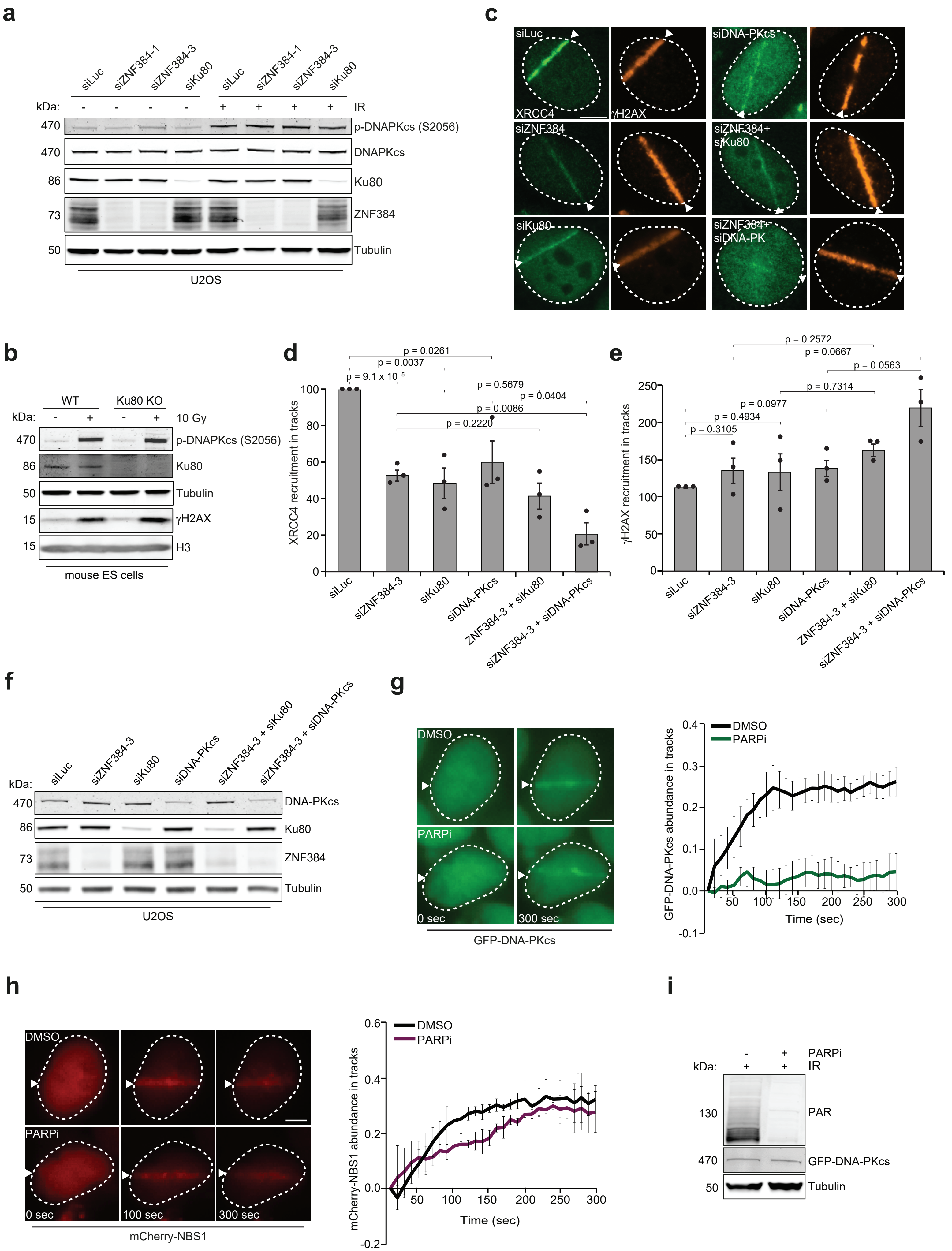

Supplementary Fig. 11 – related to Fig. 6. ZNF384 promotes recruitment of cNHEJ proteins independently of DNA-PKcs. (A) Western blot analysis of DNA-PK activation 1 hour after 10 Gy of irradiation radiation (IR) in U2OS cells transfected with the indicated siRNAs. Blots were probed for p-DNA-PKcs (S2056), DNA-PKcs, Ku80 and ZNF384. Tubulin is a loading control. Data shown represent 3 independent experiments. (B) Western blot analysis of DNA-PK activation 1 hour after 10 Gy of irradiation radiation (IR) in wildtype and *Ku80*$^{-/-}$ mouse embryonic stem cells. Blots were probed for p-DNA-PKcs (S2056), Ku80 and γH2AX. H3 and tubulin are loading controls. Data shown represent 2 independent experiments. (C) Endogenous XRCC4 recruitment to 365 nm UV-A tracks 10 minutes after damage induction in U2OS cells transfected with the indicated siRNAs. γH2AX is a damage marker. White triangles indicate irradiated regions. (D) XRCC4 quantification of C. Data shows the mean ±SEM of >150 cells acquired in 3 independent experiments (E) γH2AX quantification of C. The mean ±SEM from 3 independent experiments is shown. Data were normalized to siLuc, which was set to 100%. (F) Western blot analysis of DNA-PKcs, Ku80, ZNF384, γH2AX and Tubulin in cells from C. Tubulin is a loading control. Data shown represent 2 independent experiments. (G) GFP-DNA-PKcs recruitment to 365 nm UV-A tracks in BrdU-sensitized HCT116 cells treated with PARPi for 1 hour before laser micro-irradiation. White triangles indicate irradiated regions (left panel). Quantification of the data is presented as the mean ±SEM of >100 cells acquired in 3 independent experiments (right panel). (H) As in G, except for mCherry-NBS1. (I) Western blot analysis of PAR levels in IR- and PARPi -treated cells from G. Cells were treated with PARPi for 1 hour before IR treatment. Blots were probed for PAR, GFP and Tubulin. Tubulin is a loading control. Data shown represent 2 independent experiments. Statistical significance was calculated with the two-tailed unpaired Student's *t* test, assuming unequal variances. Scale bar 5 μm. Source data are provided as Source Data file.

Singh et al_Supplementary Fig. 12

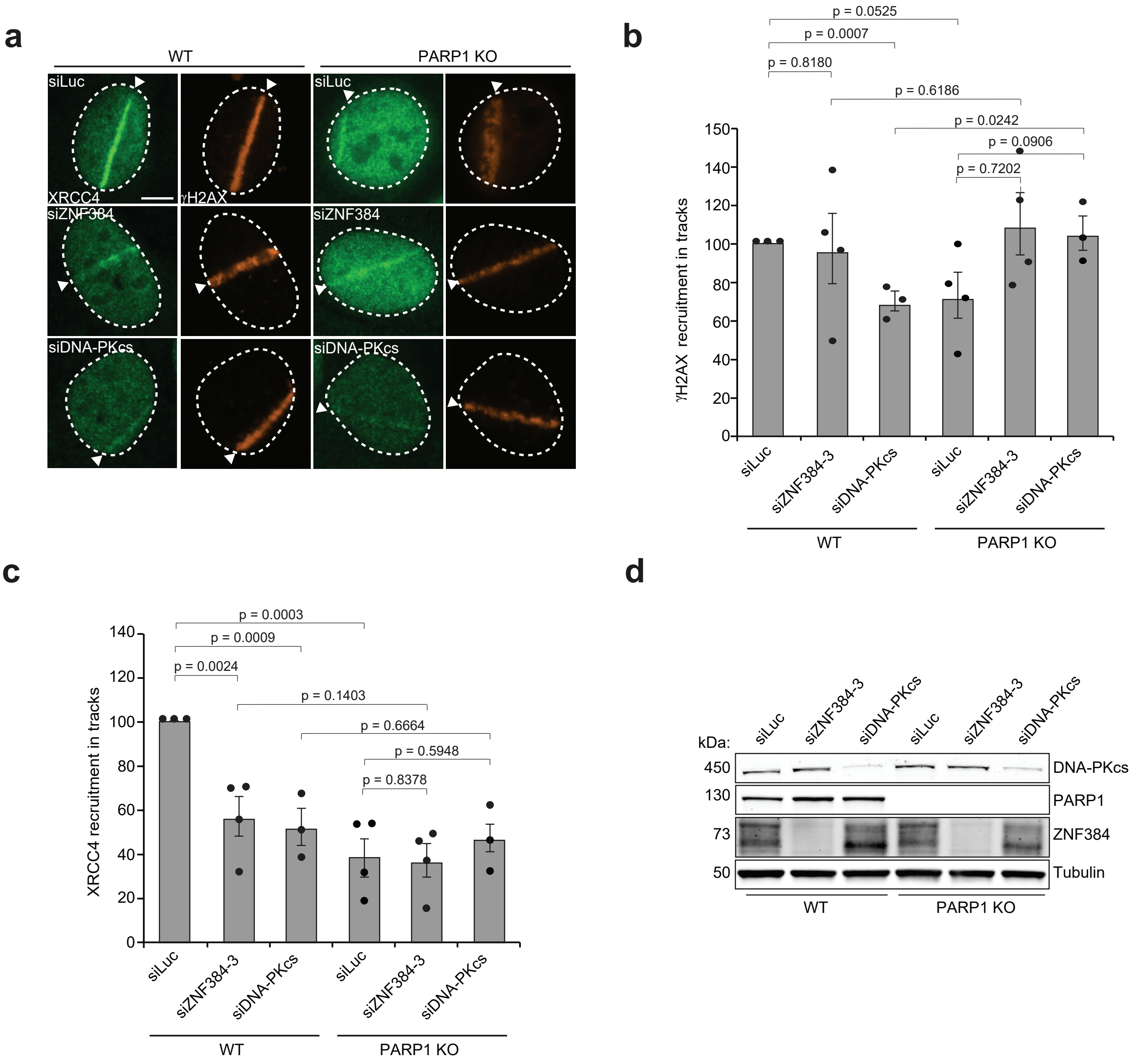

Supplementary Fig. 12 – related to Fig. 6. ZNF384 and PARP1, as well as DNA-Pkcs and PARP1, function epistatically in cNHEJ (A) Endogenous XRCC4 recruitment to 365 nm UV-A tracks in BrdU-sensitized U2OS cells transfected with the indicated siRNAs. Cells were fixed and immunostained 10 minutes after laser micro-irradiation. White triangles indicate irradiated regions. γH2AX is a damage marker. (B) γH2AX quantification of A. Data shows the mean ±SEM of >240 cells acquired in 4 independent experiments (C) XRCC4 quantification of A. The mean ±SEM from 3 independent experiments is shown. Data were normalized to siLuc, which was set to 100%. (D) Western blot analysis of ZNF384, DNA-PKcs and PARP1 in cells from A. Tubulin is a loading control. Data shown represent 2 independent experiments. Statistical significance was calculated with the two-tailed unpaired Student's *t* test, assuming unequal variances. Scale bar 5 µm. Source data are provided as Source Data file.

Singh et al_Supplementary Fig. 13

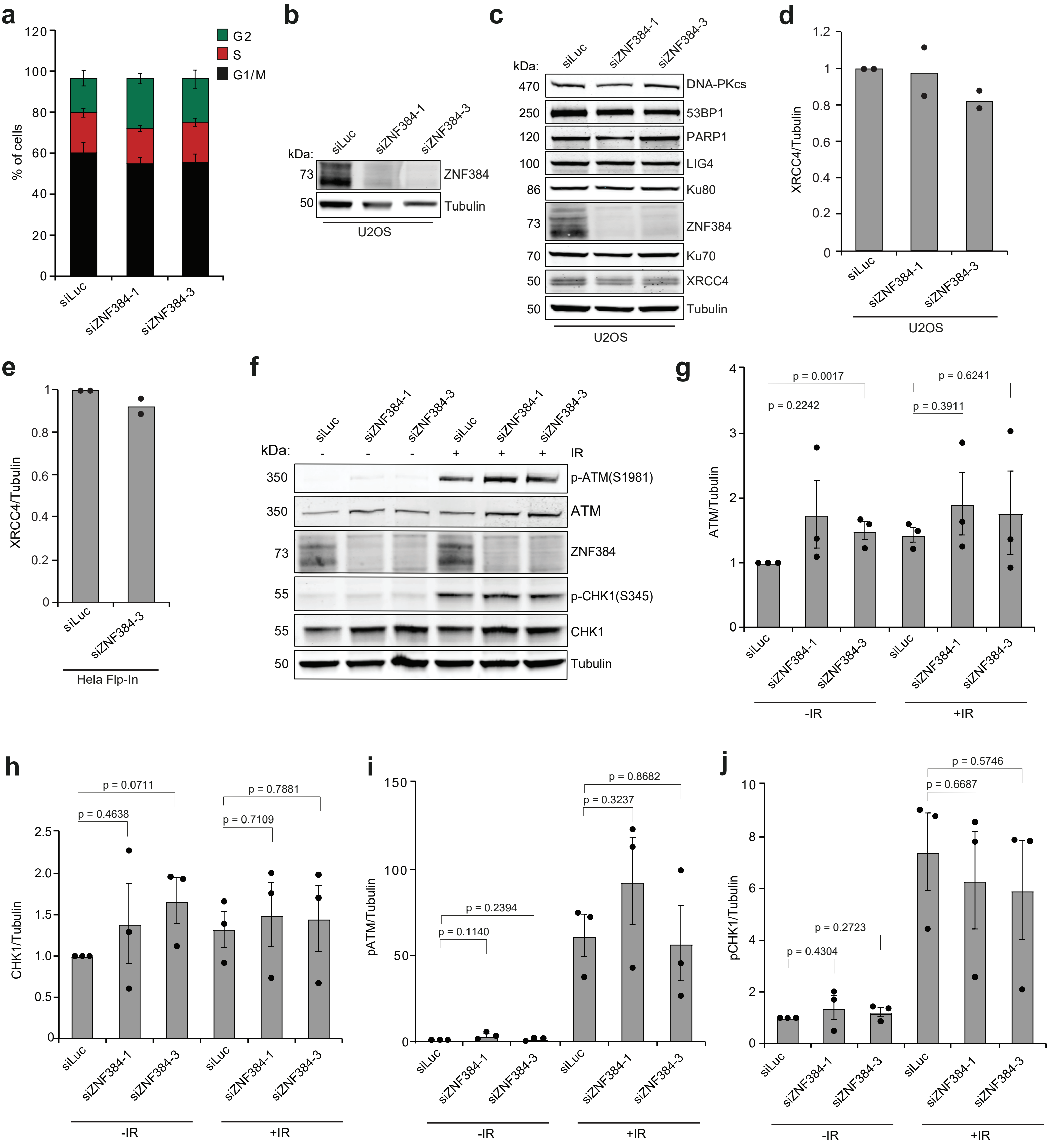

Supplementary Fig. 13 – related to Fig. 7. ZNF384 promotes efficient NHEJ independently of transcription (A) Cell cycle profile of the indicated siRNA-transfected U2OS cells. The fraction of G1-, S- and G2-phase cells was determined by propidium iodide staining and FACS analysis. >30000 cells per condition were analyzed. Data show mean ±SEM from 3-4 independent experiments. (B) Western blot analysis of ZNF384 expression in U2OS cells from A. Tubulin is a loading control. Data shown represent 2 independent experiments. (C) Western blot analysis of the expression of NHEJ factors in siRNA transfected ZNF384 knockdown U2OS cells. Blots were probed for ZNF384, 53BP1, DNA-PKcs, Ku80, Ku70, PARP1, XRCC4 and LIG4. Tubulin is a loading control. (D) Quantification of XRCC4 expression in cells from C. Tubulin is a loading control, which was used for normalization of XRCC4 expression. The mean ±SD from 2 independent experiments is shown. Data were normalized to WT + siLuc, which was set to 1. (E) Quantification of XRCC4 expression in cells from Figure 7E. Tubulin is a loading control, which was used for normalization of XRCC4 expression. The mean ±SD from 2 independent experiments is shown. Data were normalized to WT + siLuc, which was set to 1. (F) Western blot analysis of IR-induced ATM phosphorylation (p-ATM) at Ser1981 and CHK1 phosphorylation (p-CHK1) at Ser345. Cells were collected 1 hour after exposure to 10 Gy of IR. Blots were also probed for ATM, ZNF384, CHK1 and Tubulin. Tubulin is a loading control. A representative blot from 3 independent experiments is shown. (G) Quantification of ATM expression in cells from F. Tubulin was used for normalization of ATM expression. The mean ±SEM from 3 independent experiments is shown. Data were normalized to siLuc - IR, which was set to 1. (H) As in G, except for CHK1. (I) As in G, except for pATM. (J) As in G, except for pCHK1. Statistical significance was calculated with the two-tailed unpaired Student's *t* test, assuming unequal variances. Source data are provided as Source Data file.

Singh et al_Supplementary Fig. 14

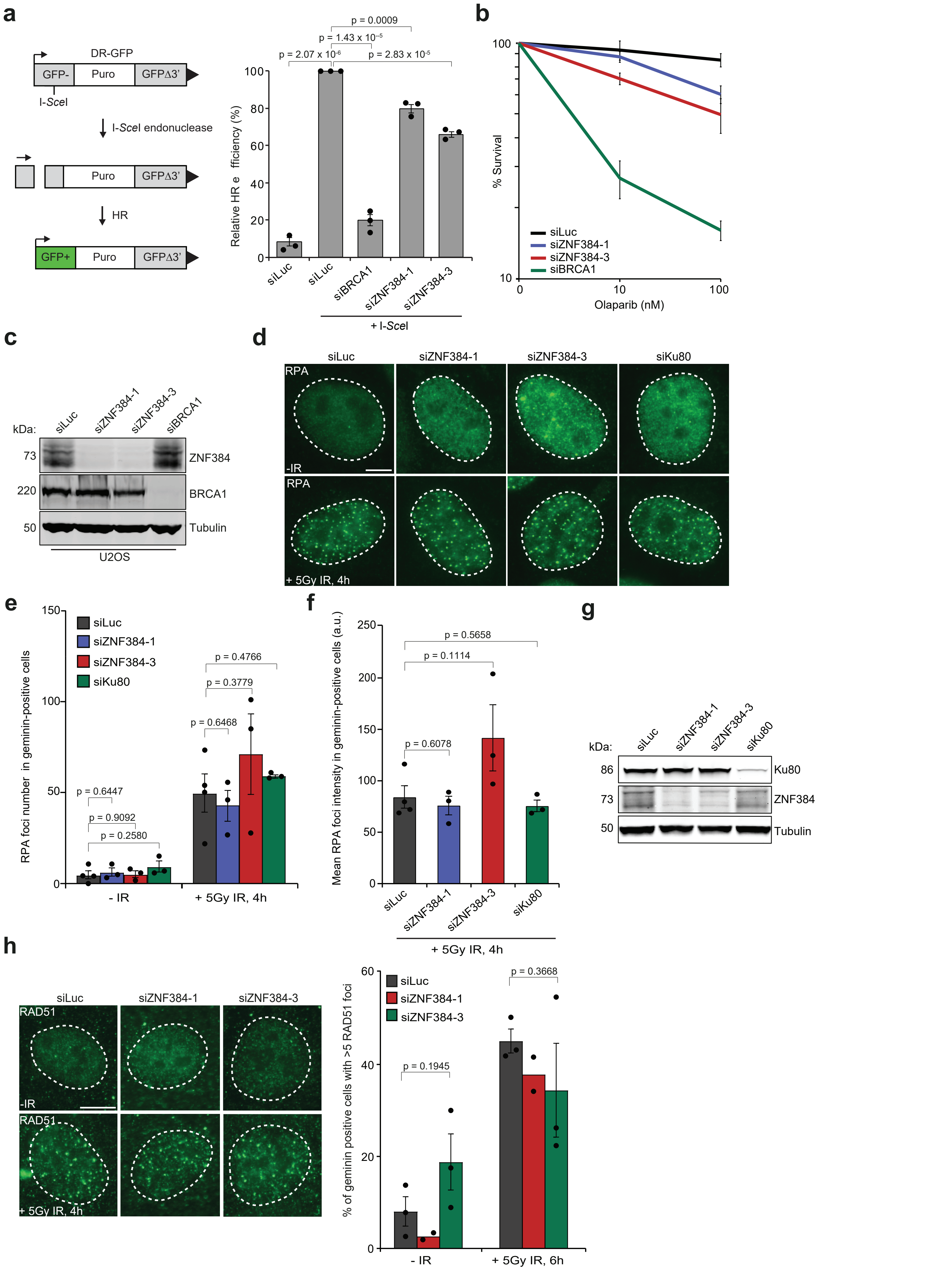

Supplementary Fig. 14 – related to Fig. 7. ZNF384 is dispensable for HR (A) Schematic of the DR-GFP reporter for HR (left panel). Quantification of HR efficiencies in 3 independent DR-GFP- U2OS transfected cells with the indicated siRNAs. I-*Sce*I transfection was corrected by co-transfection with mCherry expression vector. The mean ±SEM from 3 independent experiments is shown (right panel). (B) Clonogenic survival of U2OS cells transfected with the indicated siRNAs following treatment with the PARPi. The mean ±SEM from 3 independent experiments is shown. (C) Western blot analysis of ZNF384 and BRCA1 expression in U2OS cells. Tubulin is a loading control. Data shown represent 2 independent experiments. (D) RPA foci formation in U2OS cells transfected with the indicated siRNAs. Cells were exposed to 5 Gy of IR and foci were scored after 4 hours. (E) Quantification of RPA foci formation in cells from D. Foci were quantified in immuno-stained, geminin-positive S/G2 phase cells. The mean ±SEM from >200 cells from 3-4 independent experiments is shown. (F) As in E, except that RPA foci intensity was measured. (G) Western blot analysis of ZNF384 and Ku80 expression in cells from D. Tubulin is a loading control. (H) RAD51 foci formation in U2OS cells transfected with the indicated siRNAs. Cells were exposed to 5 Gy of IR and foci were quantified after 6 hours (left panel) The mean ±SEM from >300 cells from 2-3 independent experiments is shown. Foci were quantified in immuno-stained, geminin-positive S/G2 phase cells (right panel). Statistical significance was calculated with the two-tailed unapired Student's *t* test, assuming unequal variances. Scale bar 5 μm. Source data are provided as Source Data file.

Singh et al_Supplementary Fig. 15

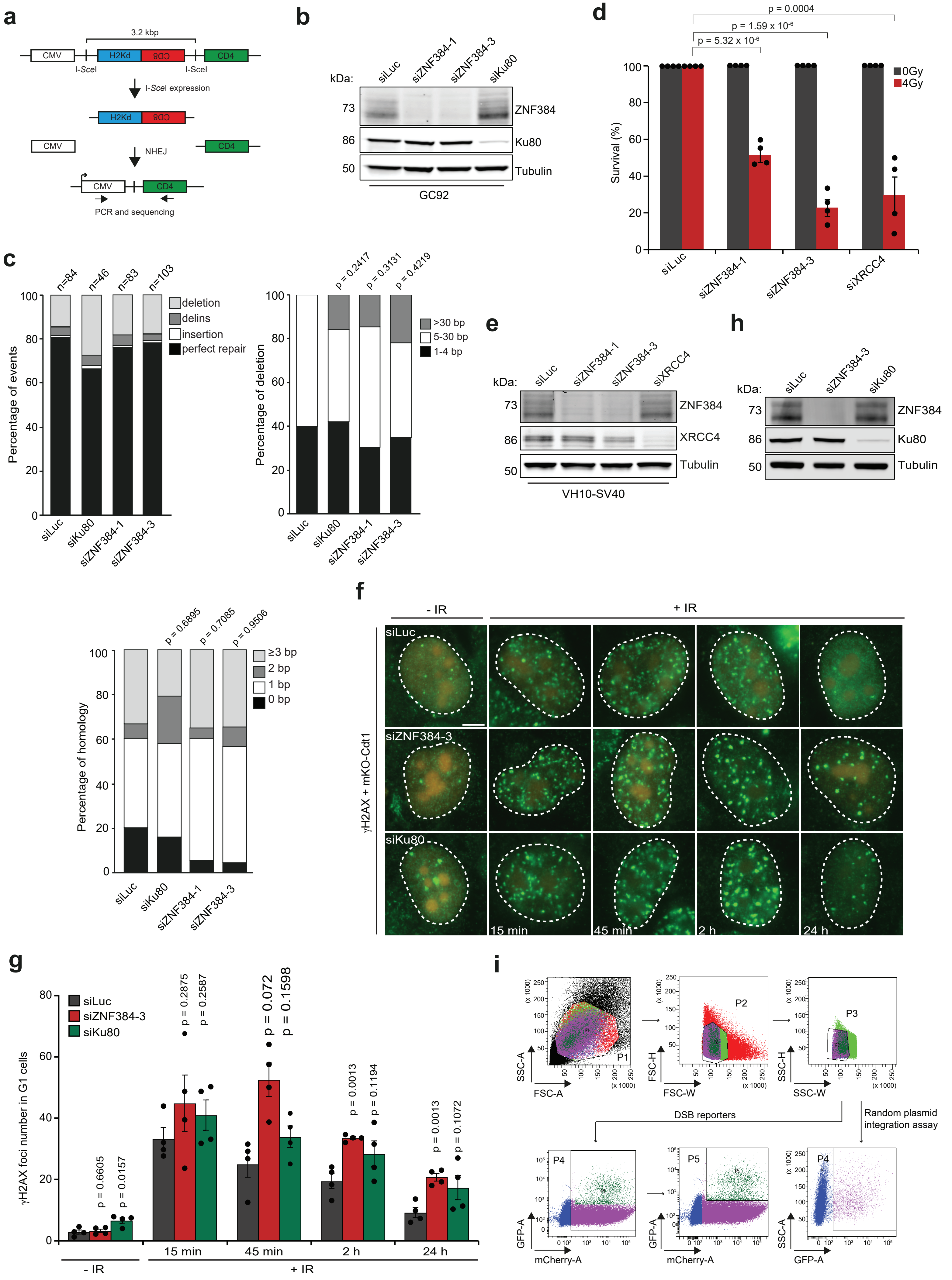

Supplementary Fig. 15 – related to Fig. 7. ZNF384 promotes DSB repair via cNHEJ (A) Schematic of the GC92 reporter for NHEJ. (B) Western blot analysis of ZNF384 and Ku80 expression in GC92 fibroblasts. Tubulin is a loading control. Data shown represent 2 independent experiments. (C) Mutational signatures (top left panel, deletion sized (top right panel) and microhomology usage (in case of deletion formation) (bottom panel) at repair junctions in GC92 reporter for NHEJ. GC92 cells were transfected with the indicated siRNAs and I-*Sce*I expression vector. Repair junctions were amplified by PCR and Sanger-sequenced. (D) Clonogenic survival of VH10-SV40 cells transfected with the indicated siRNAs and exposed to 4 Gy of IR. The mean ±SEM from 4 independent experiments is shown. P-values were calculated using the two-tailed unpaired Student's *t* test, assuming unequal variances. (E) Western blot analysis of ZNF384 and XRCC4 expression in cells used in D. Tubulin is a loading control. Data shown represent 2 independent experiments. (F) γH2AX foci formation (green) in stable Monomeric Kusabira-Orange (mKO)-Cdt1 expressing U2OS cells transfected with the indicated siRNAs. Cells were exposed to 2 Gy of IR and foci were scored after 15 minutes, 45 minutes, 2 hours and 24 hours. (G) Quantification of γH2AX foci formation in Cdt1-positive G1 phase cells from F. The mean ±SEM from 2-4 independent experiments. Statistical significance was calculated with the two-tailed unpaired Student's t-test, assuming unequal variances. (H) Western blot analysis of ZNF384 and Ku80 expression in cells from F. Tubulin is a loading control. Data shown represent 2 independent experiments. (I) Gating strategy for flow cytometric analysis of cells for DR-GFP and EJ5- GFP reporter assays, as well as random plasmid integration assays. Scale bar 10 µm. Source data are provided as a Source Data file.

Supplementary Table 1. Statistical analysis comparing LFQ intensities of proteins identified by LC-MS/MS in GFP-pulldowns from U2OS Flp-In/T-Rex cells expressing either GFP-ZNF384 or GFP-NLS. -Log p-value and average fold-enrichment (log2) are indicated. Additional information from the analysis of identified proteins is also included.

GENE			GFP-ZNF384 vs GFP			Miscellaneous													Values					
Fasta headers	Protein names	Gene names	t Significant	T-test p-valu	st Difference	Peptides	Razor +	Unique p	Sequence	Unique +	Unique s	Mol. weigh	Q-value	Score	Intensity	MS/MS	Protein I	Majority	GFP-1	GFP-2	GFP-3	ZNF384-	ZNF384-	ZNF384-3
sp|Q8TF68-2|ZN	Zinc finger protein	ZNF384	+	5,13	13,01	22	22	19	46,9	46,9	39	56,105	0	323,3	3E+11	442	Q8TF68-	Q8TF68-	23,73	24,28	22,87	36,54	36,46	36,91
sp|P13010|XRC	X-ray repair cross-	XRCC5	+	3,46	7,90	28	28	28	54,8	54,8	54,8	82,704	0	323,3	1,3E+10	116	P13010;	P13010	23,98	23,36	25,68	32,40	32,19	32,12
sp|P12956|XRC	X-ray repair cross-	XRCC6	+	5,33	7,85	32	32	32	51,4	51,4	51,4	69,842	0	320,9	1,5E+10	179	P12956;	P12956;	24,32	24,49	24,99	32,36	32,31	32,69
sp|Q9NUA8|ZBT	Zinc finger and BT	ZBTB40	+	1,85	6,21	34	34	34	34,7	34,7	34,7	138,12	0	323,3	7,5E+09	158	Q9NUA8	Q9NUA8	27,83	24,02	23,31	32,29	30,63	30,89
sp|P34932|HSP	Heat shock 70 kDa	HSPA4	+	2,93	5,41	23	23	21	30,7	30,7	27,9	94,33	0	216,5	3,7E+09	92	P34932;	P34932;	25,10	23,90	26,12	30,70	30,33	30,33
sp|P11021|BIP_	78 kDa glucose-re	HSPA5	+	5,49	4,23	42	42	41	56,6	56,6	56,6	72,332	0	323,3	5,5E+10	331	P11021	P11021	30,11	29,91	30,07	34,31	34,07	34,40
sp|P11142|HSP	Heat shock cognat	HSPA8	+	5,01	3,94	39	38	21	61,3	61,3	35,6	70,897	0	323,3	1,7E+11	418	P11142;	P11142;	31,97	31,91	31,94	35,88	35,64	36,12
sp|Q08209-2|PP	Serine/threonine-p	PPP3CA	+	2,84	3,74	13	13	7	26,4	26,4	14,7	57,658	0	37,58	2E+09	50	Q08209-	Q08209-	25,02	25,99	26,08	30,10	28,97	29,25
tr|A0A0G2JIW1|	Heat shock 70 kDa	HSPA1B;H	+	5,25	3,73	28	24	11	51,1	46,6	22,4	70,108	0	323,3	2,5E+10	172	A0A0G2	A0A0G2	29,42	29,29	29,50	33,16	32,95	33,29
sp|P09874|PARI	Poly [ADP-ribose]	PARP1	+	4,54	3,68	47	47	47	48,6	48,6	48,6	113,08	0	323,3	2,8E+10	281	P09874;	P09874	29,80	29,72	29,27	33,37	33,25	33,19
sp|P38646|GRP	Stress-70 protein,	HSPA9	+	4,85	3,63	40	40	40	55,8	55,8	55,8	73,68	0	323,3	5E+10	345	P38646;	P38646	30,50	30,41	30,48	34,07	33,86	34,35
sp|Q92598-2|HS	Heat shock protein	HSPH1	+	4,81	3,50	22	21	20	31,8	30,8	29,1	92,115	0	260	7,2E+09	99	Q92598-	Q92598-	28,00	27,68	27,77	31,44	31,12	31,40
sp|O15294|OGT	UDP-N-acetylgluc	OGT	+	3,64	3,38	4	4	4	6,3	6,3	6,3	116,92	0	17,23	3E+08	12	O15294;	O15294;	23,04	23,75	23,58	26,73	26,62	27,16
sp|P14618|KPYI	Pyruvate kinase PI	PKM	+	4,89	3,26	35	35	35	56,9	56,9	56,9	57,936	0	323,3	7E+10	312	P14618;	P14618;	31,18	31,29	31,35	34,43	34,42	34,77
sp|Q93009|UBP	Ubiquitin carboxyl-	USP7	+	1,82	3,20	6	6	6	6,7	6,7	6,7	128,3	0	18,33	2,7E+08	18	Q93009;	Q93009;	24,17	21,87	23,99	26,05	26,65	26,93
sp|P16298|PP2E	Serine/threonine-p	PPP3CB	+	2,92	2,96	9	3	3	19,7	8,2	8,2	59,024	0	10,24	2,2E+08	4	P16298;	P16298;	23,36	23,92	22,97	26,05	26,26	26,82
sp|P35251-2|RF	Replication factor	RFC1	+	3,64	2,92	12	12	12	11,8	11,8	11,8	128,18	0	37,24	1E+09	35	P35251-	P35251-	25,61	25,38	25,54	28,24	28,17	28,87
tr|E7EWP2|E7E	Triple functional d	TRIO	+	3,16	2,89	4	4	4	2,2	2,2	2,2	262,68	0	4,157	2,3E+08	5	E7EWP	E7EWP2	23,82	22,89	23,66	26,31	26,19	26,54
sp|O43143|DHX	Pre-mRNA-splicin	DHX15		1,01	2,89	6	6	6	9,1	9,1	9,1	90,932	0	9,827	4,6E+08	8	O43143	O43143	23,15	22,53	26,67	26,28	27,51	27,23
sp|Q9Y3F4|STR	Serine-threonine k	STRAP	+	1,86	2,50	4	4	4	17,7	17,7	17,7	38,438	0	16,15	2,3E+08	5	Q9Y3F4	Q9Y3F4	22,67	24,12	24,67	26,32	26,28	26,35
sp|P28482|MK0	Mitogen-activated	MAPK1	+	3,73	2,38	4	4	4	15,6	15,6	15,6	41,389	0	20,78	3,4E+08	12	P28482;	P28482;	24,46	24,21	24,63	26,82	26,58	27,04
sp|Q08J23-3|NS	tRNA (cytosine(34	NSUN2		1,22	2,34	3	3	3	8,3	8,3	8,3	59,383	0	9,354	1,2E+08	4	Q08J23-	Q08J23-	23,02	24,52	21,42	25,31	25,20	25,48
tr|H0Y4R1|H0Y4	Inosine-5-monoph	IMPDH2	+	3,21	2,27	10	10	10	23,2	23,2	23,2	50,957	0	112,5	3,6E+09	74	H0Y4R1	H0Y4R1	27,99	27,68	28,09	30,06	29,93	30,57
sp|Q8NHY2-2|C	E3 ubiquitin-protei	RFWD2	+	2,17	2,19	2	2	2	4,7	4,7	4,7	77,69	0	10,22	9,6E+07	5	Q8NHY2	Q8NHY2	23,45	22,25	23,41	25,24	24,95	25,48
sp|O14744-2|AN	Protein arginine N-	PRMT5	+	1,72	2,05	4	4	4	9,4	9,4	9,4	71,319	0	13,39	2E+08	6	O14744-	O14744-	23,19	24,46	24,13	26,50	25,23	26,22
tr|A0A2C9F2P4|	Palmitoyl-protein t	PPT1		0,97	1,99	2	2	2	10,8	10,8	10,8	34,136	0	8,25	2,8E+08	5	A0A2C9	A0A2C9	23,28	25,61	22,42	25,66	25,70	25,91
sp|P35249|RFC	Replication factor	RFC4	+	1,77	1,94	6	6	6	26,4	26,4	26,4	39,681	0	121,2	5E+08	15	P35249;	P35249;	25,62	25,73	24,41	27,01	26,87	27,70
tr|B4DXZ6|B4D	Fragile X mental r	FXR1	+	3,19	1,90	8	8	8	19,9	19,9	19,9	68,326	0	73,33	1,2E+09	31	B4DXZ6	B4DXZ6	26,59	26,80	26,52	28,37	28,35	28,90
sp|P60903|S10A	Protein S100-A10	S100A10	+	1,73	1,90	2	2	2	35,1	35,1	35,1	11,203	0	55,54	4,4E+09	33	P60903	P60903	28,96	28,61	27,60	29,74	30,44	30,69
sp|P35250-2|RF	Replication factor	RFC2	+	1,59	1,88	3	3	3	11,9	11,9	11,9	35,243	0	8,589	2,7E+08	4	P35250-	P35250-	24,16	25,29	23,84	26,09	25,91	26,93
sp|O95816|BAG	BAG family molec	BAG2	+	3,98	1,72	6	6	6	29,9	29,9	29,9	23,772	0	47,15	9,6E+08	31	O95816;	O95816;	26,28	26,47	26,62	28,24	28,07	28,21
sp|Q9Y3I0|RTCI	tRNA-splicing liga	RTCB	+	2,72	1,57	10	10	10	26,1	26,1	26,1	55,21	0	32,71	1,3E+09	30	Q9Y3I0	Q9Y3I0	27,14	26,92	26,79	28,38	28,28	28,89
sp|Q9HB71|CYE	Calcyclin-binding	CACYBP	+	2,12	1,56	2	2	2	16,2	16,2	16,2	26,21	0	14,21	1,6E+08	4	Q9HB71	Q9HB71	23,80	23,36	24,26	25,56	25,02	25,51
sp|Q9Y224|RTR	UPF0568 protein	C14orf166	+	2,52	1,49	5	5	5	25,8	25,8	25,8	28,068	0	30,36	7,9E+08	31	Q9Y224;	Q9Y224;	26,61	26,45	26,01	27,82	27,60	28,11
sp|O43684-2|BU	Mitotic checkpoint	BUB3		0,82	1,48	3	3	3	9,5	9,5	9,5	36,954	0	4,72	1,5E+08	6	O43684-	O43684-	25,09	22,23	23,78	25,19	24,93	25,41
sp|P62258|1433	14-3-3 protein eps	YWHAE		0,73	1,47	5	2	2	18,8	9	9	29,174	0	5,386	2,5E+08	15	P62258;	P62258;	23,23	24,09	26,15	26,50	25,42	25,97
tr|A0A0A0MQS1	Pyrroline-5-carbox	PYCRL	+	2,16	1,46	2	2	2	7,7	7,7	7,7	29,892	0	4,908	9E+07	5	A0A0A0	A0A0A0	23,68	23,61	22,97	25,22	24,65	24,77
tr|F1T0B3|F1T0	ATP-dependent R	DDX1	+	2,41	1,43	7	7	7	14,7	14,7	14,7	73,915	0	54,44	8,5E+08	39	F1T0B3;	F1T0B3;	26,53	26,24	26,67	27,72	27,70	28,32
tr|J3QLD9|J3QL	Flotillin-2	FLOT2		0,45	1,43	6	6	6	15,7	15,7	15,7	47,142	0	30,52	1,5E+08	9	J3QLD9;	J3QLD9;	23,34	22,78	23,33	23,37	23,07	27,30
sp|P46063|REC	ATP-dependent D	RECQL		0,73	1,42	4	4	4	7,9	7,9	7,9	73,457	0	13,97	3,6E+08	5	P46063	P46063	26,02	25,81	23,27	26,54	26,37	26,44
sp|P40937-2|RF	Replication factor	RFC5	+	1,66	1,36	4	4	4	17,9	17,9	17,9	36,104	0	70,34	3,5E+08	13	P40937-	P40937-	25,51	25,10	25,00	26,08	26,41	27,22
tr|C9K025|C9K0	60S ribosomal pro	RPL35A		1,17	1,26	2	2	2	20,2	20,2	20,2	10,645	0	4,883	6,1E+07	5	C9K025;	C9K025;	23,54	23,29	23,70	23,86	24,90	25,55
sp|O75955|FLO	Flotillin-1	FLOT1		0,41	1,26	4	4	4	12,4	12,4	12,4	47,355	0	70,13	1,9E+08	5	O75955;	O75955;	23,68	23,99	23,71	23,71	23,80	27,64
tr|F8VPD4|F8VF	CAD protein;Gluta	CAD	+	2,69	1,24	14	14	14	8,3	8,3	8,3	236,02	0	51,4	8,9E+08	41	F8VPD4	F8VPD4	26,83	26,50	26,76	28,07	27,65	28,08
sp|P53396-3|AC	ATP-citrate syntha	ACLY		0,66	1,17	2	2	2	2,8	2,8	2,8	91,098	0	3,386	9E+07	3	P53396-	P53396-	23,95	21,76	24,19	24,91	24,28	24,22
tr|A0A1B0GUA1	Alpha-aminoadipic	ALDH7A1	+	2,57	1,07	5	5	5	16,9	16,9	16,9	56,465	0	26,72	4,3E+08	13	A0A1B0	A0A1B0	25,73	25,95	25,58	27,06	26,69	26,72
sp|P07195|LDHE	L-lactate dehydrog	LDHB		0,44	1,06	9	9	9	32,9	32,9	32,9	36,638	0	65,2	2E+09	37	P07195;	P07195;	27,20	27,14	28,85	30,23	28,87	27,26
sp|P11166|GTR	Solute carrier fami	SLC2A1		0,64	1,06	2	2	2	3,7	3,7	3,7	54,083	0	4,252	1,5E+08	10	P11166	P11166	24,80	24,80	22,58	25,18	24,96	25,20
tr|A0A0A0MR02	Voltage-dependen	VDAC2	+	1,66	1,00	7	7	7	33,7	33,7	33,7	30,348	0	82,09	1,5E+09	41	A0A0A0	A0A0A0	27,53	27,52	27,96	28,72	28,24	29,03
sp|P48681|NES	Nestin	NES		0,27	0,98	11	11	11	8,3	8,3	8,3	177,44	0	62,37	3,8E+08	18	P48681	P48681	24,81	24,18	24,48	23,17	28,09	25,15
tr|A0A3B3ISG8|	NADH dehydroger	NDUFA9	+	2,09	0,97	4	4	4	7,8	7,8	7,8	64,217	0	7,892	2,1E+08	10	A0A3B3	A0A3B3	24,97	24,66	24,75	26,08	25,48	25,74
sp|Q9UJS0|CMC	Calcium-binding m	SLC25A13	+	1,70	0,97	6	6	6	11,4	11,4	11,4	74,175	0	23,64	3E+08	12	Q9UJS0	Q9UJS0	25,49	25,19	25,35	26,11	26,03	26,80
sp|P06748-2|NP	Nucleophosmin	NPM1	+	1,56	0,96	5	5	5	27,2	27,2	27,2	29,464	0	113,6	1,8E+09	39	P06748-	P06748-	28,27	27,83	27,37	28,58	28,97	28,81
sp|O14818-2|PS	Proteasome subur	PSMA7;PSMA8		0,52	0,96	3	3	3	22,5	22,5	22,5	20,193	0	6,969	2,1E+08	11	O14818-	O14818-	23,16	25,59	25,56	25,69	25,79	25,70
sp|P40938|RFC	Replication factor	RFC3		0,79	0,94	3	3	3	9	9	9	40,556	0	49,26	1,2E+08	10	P40938;	P40938;	23,93	23,67	24,18	24,44	24,25	25,94
sp|P67775-2|PP	Serine/threonine-p	PPP2CA;P	+	2,06	0,93	2	2	2	7,5	7,5	7,5	29,734	0	6,06	1,7E+08	15	P67775-	P67775-	24,87	24,48	24,24	25,54	25,34	25,51
sp|Q96CV9-3|OI	Optineurin	OPTN	+	1,32	0,91	2	2	2	3,7	3,7	3,7	59,559	0	6,114	2,9E+07	7	Q96CV9	Q96CV9	22,73	22,12	22,94	23,40	23,21	23,91
tr|Q5QPM0|Q5C	RNA-binding prote	RALY		1,28	0,88	5	5	5	28,7	28,7	28,7	18,66	0	13,49	4,8E+08	27	Q5QPM	Q5QPM	26,08	25,92	26,31	27,50	26,45	27,00
tr|E9PLK3|E9PL	Puromycin-sensitiv	NPEPPS;NPEPPSL1		0,53	0,87	4	4	4	5,8	5,8	5,8	102,99	0	4,855	1,9E+08	5	E9PLK3	E9PLK3	25,17	23,10	25,33	25,35	25,45	25,40
sp|P11413|G6PI	Glucose-6-phosph	G6PD	+	1,86	0,86	5	5	5	10,7	10,7	10,7	59,256	0	8,795	3,5E+08	11	P11413;	P11413;	25,84	25,50	25,54	26,65	26,14	26,66
sp|P30153|2AA	Serine/threonine-p	PPP2R1A	+	2,58	0,84	5	5	5	12,2	12,2	12,2	65,308	0	26,47	6,5E+08	25	P30153;	P30153;	26,43	26,61	26,52	27,58	27,20	27,31
tr|E9PMD7|E9PI	Serine/threonine-p	PPP1CA;PPP1CC;PPP		0,62	0,84	2	2	2	9,9	9,9	9,9	28,898	0	5,82	1,7E+08	8	E9PMD7	E9PMD7	25,08	23,23	24,83	24,98	25,59	25,08
tr|H9KV75|H9KV	Alpha-actinin-1	ACTN1		1,27	0,82	8	8	5	14,2	14,2	9,4	94,825	0	66,66	6,5E+08	25	H9KV75	H9KV75	26,54	26,06	26,92	27,67	27,19	27,13
sp|P31948-3|ST	Stress-induced-ph	STIP1		0,60	0,81	3	3	3	7,1	7,1	7,1	59,777	0	7,066	2E+08	12	P31948-	P31948-	24,72	24,47	24,80	26,67	24,97	24,80
tr|E9PEB5|E9PE	Far upstream elem	FUBP1	+	2,06	0,81	8	8	6	12,4	12,4	10,1	68,903	0	13,48	5,1E+08	20	E9PEB5	E9PEB5	26,18	26,10	26,49	27,31	26,95	26,95
sp|P78346|RPP	Ribonuclease P pr	RPP30		1,27	0,81	2	2	2	14,2	14,2	14,2	29,321	0	15,71	9,1E+07	4	P78346;	P78346;	24,24	23,29	23,74	24,37	24,55	24,78
sp|O00429-4|DN	Dynamin-1-like pro	DNM1L	+	2,38	0,80	5	5	5	8,2	8,2	8,2	78,099	0	12,76	2,5E+08	9	O00429-	O00429-	25,09	25,17	25,36	26,23	25,91	25,89
sp|Q86UT8|CCD	Coiled-coil domain	CCDC84		1,13	0,77	2	2	2	6	6	6	37,974	0	3,936	1,5E+08	7	Q86UT8	Q86UT8	24,36	24,52	24,63	25,63	24,65	25,54
sp|Q86V81|THO	THO complex sub	ALYREF		0,16	0,77	5	5	5	26,5	26,5	26,5	26,888	0	55,82	6,3E+08	15	Q86V81;	Q86V81;	28,29	26,22	22,57	26,11	27,48	25,79
sp|Q15233-2|NC	Non-POU domain-	NONO		1,01	0,75	7	7	6	18,6	18,6	16,5	43,866	0	20,84	9,3E+08	37	Q15233-	Q15233-	26,98	27,06	27,33	28,50	27,38	27,73
tr|F8WCJ1|F8W	Eukaryotic translat	EIF5A2;EIF	+	1,54	0,75	2	2	2	19	19	19	11,687	0	5,912	4E+08	17	F8WCJ1	F8WCJ1	26,22	25,93	25,66	26,94	26,40	26,72
tr|F8VXC8|F8V	SWI/SNF complex	SMARCC2;	+	1,44	0,74	3	3	3	3,3	3,3	3,3	136,18	0	5,404	1,3E+08	8	F8VXC8	F8VXC8	24,40	24,26	24,08	25,42	24,78	24,75
sp|P22234|PUR	Multifunctional pro	PAICS	+	2,40	0,74	9	9	9	24,5	24,5	24,5	47,079	0	88,41	2,1E+09	55	P22234;	P22234;	28,35	28,10	28,37	29,12	28,84	29,07
sp|Q9H0D6-2|XI	5-3 exoribonucleas	XRN2		1,16	0,73	2	2	2	3,4	3,4	3,4	99,96	0	5,324	1,1E+08	8	Q9H0D6	Q9H0D6	24,24	23,50	24,30	24,91	24,44	24,87
sp|O75531|BAF_	Barrier-to-autointe	BANF1		0,23	0,73	2	2	2	42,7	42,7	42,7	10,058	0	10,16	4,5E+08	10	O75531	O75531	26,36	23,38	27,12	26,07	27,17	25,80
sp|P19784|CSK	Casein kinase II su	CSNK2A2	+	1,45	0,73	3	3	3	11,4	11,4	11,4	41,213	0	7,586	2,7E+08	14	P19784;	P19784;	25,68	25,26	25,03	25,94	25,89	26,32
sp|Q9Y265|RUV	RuvB-like 1	RUVBL1	+	1,61	0,72	3	3	3	10,3	10,3	10,3	50,227	0	35,52	6,2E+08	14	Q9Y265;	Q9Y265;	26,81	26,51	26,30	27,34	26,99	27,46
sp|P78527-2|PR	DNA-dependent pr	PRKDC	+	2,15	0,70	33	33	33	9,6	9,6	9,6	465,5	0	185,9	3,7E+09	170	P78527-	P78527-	29,28	29,06	29,06	30,00	29,61	29,90
tr|F6VDH7|F6VD	Putative protein F	ST13;ST13P4;ST13P5		1,25	0,70	4	4	4	30,9	30,9	30,9	18,085	0	20,84	4,2E+08	19	F6VDH7	F6VDH7	26,09	25,74	25,99	27,10	26,29	26,54
sp|Q9Y230|RUV	RuvB-like 2	RUVBL2	+	1,44	0,69	5	5	5	14,3	14,3	14,3	51,156	0	9,23	4,9E+08	13	Q9Y230;	Q9Y230;	26,37	26,33	25,94	27,01	26,56	27,13
sp|P21796|VDA	Voltage-dependen	VDAC1		1,04	0,67	12	12	12	56,9	56,9	56,9	30,772	0	172,3	2,8E+09	49	P21796;	P21796;	28,77	28,39	28,99	29,68	28,89	29,59
tr|H7BY10|H7BY	60S ribosomal pro	RPL23A		0,20	0,66	3	3	3	20,3	20,3	20,3	17,692	0	4,89	3,7E+08	7	H7BY10	H7BY10	26,84	25,37	23,24	24,68	27,09	25,65
sp|Q09028-3|RB	Histone-binding pr	RBBP4;RB	+	1,65	0,66	4	4	4	9,3	9,3	9,3	46,158	0	11,59	5,3E+08	15	Q09028-	Q09028-	26,30	26,27	26,44	27,34	26,89	26,77
sp|P31153-2|ME	S-adenosylmethio	MAT2A		0,21	0,66	2	2	2	8	8	8	32,964	0	9,62	1,3E+08	11	P31153-	P31153-	22,67	26,20	22,57	24,37	24,23	24,80
tr|E7ENZ3|E7EN	T-complex protein	CCT5		0,97	0,65	8	8	8	18,9	18,9	18,9	53,848	0	13,36	7,5E+08	15	E7ENZ3	E7ENZ3	26,95	26,69	27,08	28,13	27,22	27,30
tr|B4DJ81|B4DJ	NADH-ubiquinone	NDUFS1		0,68	0,64	2	2	2	4,3	4,3	4,3	66,921	0	7,546	9,7E+07	8	B4DJ81;	B4DJ81;	24,05	23,70	23,94	25,36	24,07	24,17
sp|Q08211|DHX	ATP-dependent R	DHX9		1,22	0,61	5	5	5	4,8	4,8	4,8	140,96	0	15,5	3,6E+08	9	Q08211;	Q08211	25,83	25,62	26,01	26,62	26,65	26,01
sp|P31930|QCR	Cytochrome b-c1	UQCRC1		0,66	0,60	4	4	4	13,1	13,1	13,1	52,645	0	17,89	3,2E+08	12	P31930	P31930	25,24	25,65	25,88	26,59	25,46	26,52
sp|P33993-2|MC	DNA replication lic	MCM7		1,25	0,60	3	3	3	12,6	12,6	12,6	44,648	0	25,72	3E+08	13	P33993-	P33993-	25,83	25,28	25,34	26,21	26,23	25,79
tr|J3KR24|J3KR	Isoleucine--tRNA l	IARS	+	3,08	0,59	3	3	3	2,4	2,4	2,4	131,76	0	5,873	2,2E+08	12	J3KR24;	J3KR24;	25,25	25,18	25,11	25,82	25,67	25,83
sp|P30041|PRD	Peroxiredoxin-6	PRDX6		1,04	0,58	6	6	6	34,4	34,4	34,4	25,035	0	49,95	5,9E+08	18	P30041	P30041	26,62	26,44	26,44	27,55	27,02	26,67
sp|P61604|CH1	10 kDa heat shock	HSPE1		0,42	0,58	3	3	3	29,4	29,4	29,4	10,932	0	5,7	1,7E+08	11	P61604;	P61604;	24,45	24,49	24,96	26,31	24,80	24,52
sp|P22087|FBRI	rRNA 2-O-methylt	FBL		0,60	0,57	5	5	5	29,9	29,9	29,9	33,784	0	43,75	4,9E+08	12	P22087;	P22087;	26,52	26,22	25,76	26,03	27,23	26,95
tr|E7EQR4|E7E	Ezrin;Radixin	EZR;RDX		0,44	0,55	5	5	2	7,7	7,7	2,9	69,371	0	8,658	3,5E+08	13	E7EQR4	E7EQR4	25,78	25,81	25,63	27,29	26,11	25,48
tr|G3V4W0|G3V	Heterogeneous nu	HNRNPC;H	+	1,63	0,54	8	8	8	32,4	32,4	32,4	28,916	0	127,8	4,1E+09	83	G3V4W	G3V4W	29,50	29,45	29,15	30,05	29,70	29,98
sp|P62195-2|PR	26S protease regu	PSMC5		0,99	0,54	3	3	3	9,5	9,5	9,5	44,784	0	24,58	2,6E+08	21	P62195-	P62195-	25,53	25,12	25,59	26,25	25,55	26,07
sp|P49368|TCP	T-complex protein	CCT3		1,08	0,52	10	10	10	25	25	25	60,533	0	45,24	1,3E+09	48	P49368;	P49368;	27,99	27,53	27,86	28,64	28,02	28,30
sp|P62993-2|GR	Growth factor rece	GRB2		0,61	0,52	2	2	2	10,8	10,8	10,8	20,557	0	3,69	1,5E+08	6	P62993-	P62993-	25,13	23,88	24,43	24,77	25,01	25,21
tr|A0A2R8YF43|	Casein kinase II su	CSNK2A1;	+	1,40	0,51	6	6	6	29,2	29,2	29,2	41,79	0	48,71	1,2E+09	28	A0A2R8	A0A2R8	27,69	27,70	27,41	28,06	27,90	28,38
tr|Q32Q12|Q32C	Nucleoside diphos	NME1-NME2;NME2		0,80	0,49	7	7	3	36	36	9,2	32,642	0	21,18	1,6E+09	59	Q32Q12	Q32Q12	27,93	27,95	28,33	29,05	28,40	28,23
tr|A0A087X0X3|	Heterogeneous nu	HNRNPM	+	1,79	0,49	21	21	21	38,8	38,8	38,8	77,569	0	123,3	5,4E+09	154	A0A087	A0A087	29,96	29,79	29,58	30,27	30,17	30,36
sp|O75367-2|H2	Core histone macr	H2AFY		0,45	0,48	4	4	4	18,7	18,7	18,7	39,183	0	101,3	1,1E+09	20	O75367-	O75367-	27,66	27,16	27,20	27,08	28,59	27,79
sp|O43242|PSM	26S proteasome n	PSMD3		0,67	0,48	3	3	3	7,1	7,1	7,1	60,977	0	30,02	1,4E+08	17	O43242;	O43242;	24,82	23,98	24,90	25,16	24,79	25,18
sp|Q99959-2|PK	Plakophilin-2	PKP2		0,52	0,47	3	3	3	5,3	5,3	5,3	92,755	0	8,672	8,9E+07	8	Q99959-	Q99959-	24,28	24,07	23,00	24,23	24,26	24,28
sp|P12004|PCN	Proliferating cell n	PCNA		0,80	0,47	2	2	2	5,7	5,7	5,7	28,768	0	2,896	2,1E+08	10	P12004	P12004	25,26	25,14	25,16	26,08	25,15	25,72
sp|P61981|1433	14-3-3 protein gan	YWHAG		0,48	0,46	6	2	2	23,1	9,3	9,3	28,302	0	20,5	2,5E+08	6	P61981	P61981	25,38	25,29	25,50	26,68	25,36	25,51
sp|Q92769-3|HD	Histone deacetylas	HDAC2		0,66	0,45	3	3	3	9,2	9,2	9,2	51,998	0	12,82	2,5E+08	14	Q92769-	Q92769-	25,62	24,82	25,70	26,06	25,63	25,78
tr|A0A0B4J269|	Tubulin beta-3 cha	TUBB3		0,87	0,44	16	3	3	22,5	6,5	6,5	88,381	0	10,98	3,9E+08	18	A0A0B4	A0A0B4	26,38	25,74	25,86	26,67	26,41	26,23
sp|P27348|1433	14-3-3 protein thet	YWHAQ		0,98	0,44	7	4	4	29	18,8	18,8	27,764	0	52,33	8,6E+08	20	P27348;	P27348;	27,41	27,06	27,19	27,95	27,32	27,71
sp|P08670|VIME	Vimentin	VIM		0,17	0,43	37	36	34	75,8	74,2	72,3	53,651	0	323,3	2,8E+10	278	P08670;	P08670;	31,88	31,22	32,19	30,68	33,74	32,16
tr|G5E972|G5E9	Lamina-associatec	TMPO	+	1,31	0,43	3	3	3	10,4	10,4	10,4	46,306	0	20,53	2,3E+08	10	G5E972;	G5E972;	25,32	25,06	25,46	25,86	25,75	25,53
sp|P23284|PPIB	Peptidyl-prolyl cis-	PPIB		0,37	0,42	2	2	2	10,2	10,2	10,2	23,742	0	3,341	1,3E+08	7	P23284	P23284	23,89	24,31	24,89	25,55	24,50	24,31
sp|P46060|RAG	Ran GTPase-activ	RANGAP1		0,33	0,42	3	3	3	5,6	5,6	5,6	63,541	0	6,837	2,3E+08	4	P46060;	P46060	25,57	25,62	24,03	25,50	25,52	25,46
tr|F5GWF6|F5G	T-complex protein	CCT2		0,65	0,40	12	12	12	29,1	29,1	29,1	56,806	0	44,76	1,4E+09	25	F5GWF	F5GWF6	27,87	27,60	28,36	28,66	28,17	28,19
sp|P07900|HS9	Heat shock protein	HSP90AA1		0,57	0,40	24	12	12	32,2	15,3	15,3	84,659	0	157,8	2,4E+09	72	P07900;	P07900;	28,60	28,55	29,01	29,66	28,78	28,92
sp|P00558-2|PG	Phosphoglycerate	PGK1		0,21	0,39	5	5	5	18	18	18	41,428	0	20,66	2,7E+08	6	P00558-	P00558-	25,01	25,36	25,92	27,16	25,31	24,99
sp|P05783|K1C1	Keratin, type I cyto	KRT18		0,53	0,39	16	15	10	44	42,3	32,1	48,057	0	137,9	2,7E+09	65	P05783;	P05783;	28,93	28,35	29,11	28,82	29,60	29,13
sp|P06753-4|TPI	Tropomyosin alpha	TPM3		0,64	0,39	11	11	7	38,9	38,9	24,7	28,793	0	105,3	1,7E+09	50	P06753-	P06753-	28,02	28,22	28,29	29,07	28,23	28,39
sp|O43390-3|HN	Heterogeneous nu	HNRNPR		0,72	0,39	7	7	7	14,5	14,5	14,5	66,979	0	39,74	9,3E+08	32	O43390-	O43390-	27,61	27,26	27,04	28,03	27,43	27,60
sp|P00338|LDH	L-lactate dehydrog	LDHA		0,19	0,38	12	12	12	33,7	33,7	33,7	36,688	0	41,52	1,7E+09	37	P00338;	P00338;	27,80	27,39	28,97	29,53	28,38	27,40
sp|P13489|RINI_	Ribonuclease inhit	RNH1		1,23	0,38	2	2	2	6,5	6,5	6,5	49,973	0	25,29	2,4E+08	12	P13489;	P13489;	25,28	25,22	25,68	25,77	25,76	25,80
sp|P55072|TER	Transitional endop	VCP		0,44	0,38	11	11	11	18,2	18,2	18,2	89,321	0	55,57	1,1E+09	50	P55072;	P55072	27,40	27,29	28,09	28,51	27,69	27,72
sp|P14868-2|SY	Aspartate--tRNA li	DARS	+	2,10	0,38	4	4	4	12,2	12,2	12,2	45,771	0	14,8	2,7E+08	20	P14868-	P14868-	25,56	25,64	25,58	25,96	25,85	26,10
sp|Q15293-2|RC	Reticulocalbin-1	RCN1		0,47	0,37	2	2	2	10,4	10,4	10,4	33,026	0	17,42	1,3E+08	9	Q15293-	Q15293-	24,85	24,70	23,81	24,95	24,92	24,62
sp|P52292|IMA1	Importin subunit a	KPNA2		1,05	0,35	9	9	9	26,3	26,3	26,3	57,861	0	122,2	1,5E+09	46	P52292;	P52292;	28,21	27,89	28,07	28,50	28,15	28,56
sp|P20962|PTM	Parathymosin	PTMS		0,23	0,35	1	1	1	11,8	11,8	11,8	11,53	0	25,46	8,3E+07	14	P20962	P20962	24,87	24,12	23,54	25,17	23,69	24,71
sp|Q13155|AIMF	Aminoacyl tRNA s	AIMP2		0,33	0,35	2	2	2	10,6	10,6	10,6	35,348	0	5,52	1,7E+08	8	Q13155;	Q13155;	25,09	25,33	23,94	25,19	25,01	25,20
tr|A0A087WTT1	Polyadenylate-bin	PABPC1;PABPC3		0,46	0,35	3	3	3	7,1	7,1	7,1	58,535	0	13,82	1,6E+08	11	A0A087	A0A087	24,73	24,51	25,30	25,63	25,04	24,91
sp|Q14315-2|FLI	Filamin-C	FLNC		0,86	0,34	13	9	9	6,2	4,3	4,3	287,28	0	21,51	6E+08	18	Q14315-	Q14315-	26,65	26,73	27,19	27,33	27,18	27,08
sp|Q9BVA1|TBE	Tubulin beta-2B c	TUBB2B;TUBB2A		0,43	0,34	17	3	2	45,8	10,8	8,1	49,953	0	27,86	1E+09	21	Q9BVA1	Q9BVA1	27,55	27,68	27,16	27,27	27,85	28,29
tr|C9JJT5|C9JJ	ATP synthase sub	ATP5J2-PTCD1;ATP5J2		0,70	0,34	2	2	2	44,4	44,4	44,4	5,9149	0	11,27	5,3E+08	15	C9JJT5;	C9JJT5;	26,56	26,48	26,54	27,25	26,50	26,84
sp|P24534|EF1E	Elongation factor 1	EEF1B2		0,49	0,34	3	3	3	17,8	17,8	17,8	24,763	0	36,11	6,1E+08	20	P24534;	P24534	26,78	26,50	26,99	27,58	26,66	27,04

sp|Q9UMS4|PRI	Pre-mRNA-proces	PRPF19	+	1,55	0,33	3	3	3	6	6	6	55,18	0	7,133	2,8E+08	8	Q9UMS	Q9UMS4	25,51	25,74	25,63	26,04	25,81	26,02
sp|P60468|SC61	Protein transport p	SEC61B		0,40	0,33	3	3	3	37,5	37,5	37,5	9,9743	0	12,38	3,9E+08	17	P60468;	P60468	26,60	25,81	26,09	26,89	25,99	26,62
sp|O00299|CLIC	Chloride intracellul	CLIC1		0,10	0,33	3	3	3	16,2	16,2	16,2	26,922	0	5,435	1,9E+08	5	O00299	O00299	24,30	24,44	24,74	26,62	25,38	22,47
sp|P02545|LMN	Prelamin-A/C;Lam	LMNA		0,67	0,33	16	14	14	25,9	24,5	24,5	74,139	0	95,93	1,5E+09	79	P02545;	P02545;	27,95	27,96	28,49	28,43	28,70	28,25
sp|P50990|TCP(	T-complex protein	CCT8		0,86	0,32	19	19	19	39,8	39,8	39,8	59,62	0	70,58	2,9E+09	92	P50990;	P50990;	29,09	28,91	29,20	29,66	29,13	29,37
sp|Q9NVI7-3|AT	ATPase family AA	ATAD3A		0,97	0,32	5	5	2	11,6	11,6	6,3	57,947	0	11,48	2,4E+08	12	Q9NVI7-	Q9NVI7-	25,71	25,25	25,47	25,87	25,64	25,88
sp|Q99832-4|TC	T-complex protein	CCT7		0,45	0,32	5	5	5	11,6	11,6	11,6	50,352	0	12,31	2,3E+08	11	Q99832-	Q99832-	25,34	25,20	25,55	26,26	25,39	25,39
sp|P12236|ADT	ADP/ATP transloc	SLC25A6		0,51	0,31	11	5	4	34,6	16,4	12,8	32,866	0	33,25	1,5E+09	25	P12236;	P12236	28,29	28,13	27,74	28,56	27,94	28,60
sp|P31689|DNJ	DnaJ homolog sub	DNAJA1	+	1,49	0,31	3	3	3	10,3	10,3	10,3	44,868	0	6,626	2,6E+08	11	P31689;	P31689;	25,58	25,66	25,46	26,02	25,76	25,87
tr|Q5JP53|Q5JP	Tubulin beta chain	TUBB		1,24	0,30	19	19	4	54,2	54,2	16,7	47,766	0	323,3	8,1E+10	354	Q5JP53;	Q5JP53;	33,85	33,67	33,68	34,03	33,87	34,22
tr|Q5TEC6|Q5TE	Histone H3;Histon	HIST2H3PS2;H3F3C		0,29	0,30	5	1	1	32,4	8,1	8,1	15,43	0,0013	2,289	1,7E+09	16	Q5TEC6	Q5TEC6	28,29	27,93	28,24	27,80	29,22	28,34
tr|A0A1B0GVU9	Glutamine--tRNA l	QARS		0,47	0,30	4	4	4	6,5	6,5	6,5	83,782	0	9,649	4,1E+08	15	A0A1B0	A0A1B0	26,45	25,83	26,27	26,67	26,72	26,07
tr|H0Y5H9|H0Y5	Serpin B4	SERPINB4		0,20	0,30	8	2	2	20,8	6,2	6,2	42,388	0	21,53	1,7E+08	13	H0Y5H9	H0Y5H9	24,77	23,86	25,83	25,09	25,36	24,90
sp|P07737|PRO	Profilin-1	PFN1		0,15	0,30	6	6	6	52,9	52,9	52,9	15,054	0	19,16	9,1E+08	24	P07737;	P07737;	26,84	26,70	28,17	28,51	27,55	26,54
sp|P30101|PDIA	Protein disulfide-is	PDIA3		0,27	0,30	5	5	5	13,3	13,3	13,3	56,782	0	12,85	5E+08	17	P30101;	P30101	26,25	26,24	26,76	27,50	26,45	26,19
sp|Q99714|HCD	3-hydroxyacyl-Co	HSD17B10		0,67	0,29	5	5	5	30,7	30,7	30,7	26,923	0	51,73	4,7E+08	28	Q99714;	Q99714;	26,71	26,06	26,41	26,65	26,59	26,81
tr|B4DR61|B4DF	Protein transport p	SEC61A1;SEC61A2		0,67	0,29	2	2	2	4,1	4,1	4,1	52,949	0	3,799	3,1E+08	9	B4DR61	B4DR61	25,99	25,49	25,91	26,30	25,88	26,09
sp|P63261|ACT(	Actin, cytoplasmic	ACTG1;ACTB		0,84	0,29	17	17	6	49,9	49,9	24,8	41,792	0	323,3	3,9E+10	297	P63261;	P63261;	32,69	32,58	32,73	33,25	32,83	32,77
sp|P14866|HNR	Heterogeneous nu	HNRNPL		0,90	0,29	9	9	9	25,5	25,5	25,5	64,132	0	160,3	2,3E+09	49	P14866;	P14866;	28,79	28,50	28,70	28,87	28,80	29,18
tr|G3V2K7|G3V2	Transmembrane e	TMED10		0,47	0,28	2	2	2	15	15	15	16,904	0	3,301	1,9E+08	2	G3V2K7	G3V2K7	25,18	24,97	25,09	25,83	24,99	25,26
tr|A0A2U3TZH3|	Elongation factor 1	EEF1A2		0,36	0,27	10	2	2	26,8	7,3	7,3	54,34	0	17,45	4,9E+08	18	A0A2U3	A0A2U3	26,55	26,31	26,70	27,37	26,37	26,64
tr|H0YI98|H0YI9	Dynactin subunit 2	DCTN2		1,02	0,26	2	2	2	10,4	10,4	10,4	29,326	0	4,24	1,6E+08	8	H0YI98;	H0YI98;	25,04	24,72	24,73	25,01	25,21	25,07
tr|X6RJP6|X6RJ	Transgelin-2	TAGLN2		0,24	0,25	5	5	5	29,9	29,9	29,9	21,086	0	15,38	6,2E+08	22	X6RJP6	X6RJP6	26,63	26,71	27,04	27,78	26,91	26,45
sp|P30044-2|PR	Peroxiredoxin-5, m	PRDX5		0,40	0,25	3	3	3	20,4	20,4	20,4	17,031	0	5,005	2,2E+08	5	P30044-	P30044-	25,42	25,25	25,46	26,15	25,38	25,35
sp|Q14697|GAN	Neutral alpha-gluc	GANAB		0,42	0,25	6	6	6	10,1	10,1	10,1	106,87	0	51,65	5,1E+08	17	Q14697;	Q14697;	26,65	26,49	26,55	27,08	26,32	27,04
tr|A0A1C7CYX9	Dihydropyrimidina	DPYSL2		0,51	0,25	3	3	3	5,5	5,5	5,5	73,502	0	12,16	2,3E+08	15	A0A1C7	A0A1C7	25,50	25,48	25,27	26,01	25,31	25,66
sp|Q14204|DYH	Cytoplasmic dynei	DYNC1H1		0,90	0,25	13	13	13	3,3	3,3	3,3	532,4	0	55,08	6,2E+08	32	Q14204;	Q14204	26,99	26,70	26,75	27,02	26,92	27,24
sp|Q13268|DHR	Dehydrogenase/re	DHRS2		0,53	0,24	6	6	6	27,9	27,9	27,9	29,926	0	116,2	4,8E+09	34	Q13268;	Q13268;	30,09	29,58	29,51	29,85	30,14	29,91
sp|P63104|1433	14-3-3 protein zeta	YWHAZ		0,33	0,24	9	6	6	40	29,8	29,8	27,745	0	136,5	1,8E+09	39	P63104;	P63104;	28,37	28,17	28,56	29,17	28,22	28,43
tr|A0A494C1L1|	Lamin-B receptor	LBR		0,49	0,24	4	4	4	13,6	13,6	13,6	46,925	0	24,73	4E+08	21	A0A494	A0A494	26,15	26,20	26,36	26,86	26,16	26,41
sp|Q00325-2|MF	Phosphate carrier	SLC25A3		0,62	0,24	8	8	8	21,3	21,3	21,3	39,958	0	19,38	2,1E+09	53	Q00325-	Q00325-	28,72	28,65	28,53	29,17	28,60	28,85
sp|P42704|LPPI	Leucine-rich PPR	LRPPRC		0,37	0,24	4	4	4	2,7	2,7	2,7	157,9	0	5,152	1,6E+08	7	P42704;	P42704	25,14	24,62	24,79	25,52	24,76	24,98
sp|P16989-2|YB	Y-box-binding prot	YBX3		0,50	0,24	4	2	2	25,4	14,9	14,9	31,947	0	47,77	3,8E+08	8	P16989-	P16989-	26,37	26,18	25,69	26,43	26,21	26,32
sp|Q13347|EIF3	Eukaryotic translat	EIF3I		0,22	0,24	2	2	2	8,6	8,6	8,6	36,501	0	8,185	1,3E+08	3	Q13347	Q13347	24,96	24,68	23,61	24,47	24,75	24,74
sp|P22695|QCR	Cytochrome b-c1	UQCRC2		0,49	0,23	6	6	6	22,1	22,1	22,1	48,442	0	96,89	1E+09	26	P22695;	P22695;	27,51	27,52	27,69	28,17	27,48	27,77
tr|A0A286YFA2|	D-3-phosphoglyce	PHGDH		0,57	0,23	5	5	5	14,6	14,6	14,6	47,603	0	25,45	1,1E+09	31	A0A286	A0A286	27,50	27,73	27,77	27,97	27,59	28,14
sp|P05141|ADT2	ADP/ATP transloc	SLC25A5		0,68	0,23	11	11	4	38,3	38,3	14,4	32,852	0	71,77	2,2E+10	124	P05141	P05141	32,12	31,88	31,93	32,24	31,96	32,42
sp|Q06830|PRD	Peroxiredoxin-1	PRDX1		0,86	0,23	16	16	12	55,8	55,8	40,7	22,11	0	74,1	5E+10	168	Q06830;	Q06830;	33,24	33,13	33,12	33,30	33,25	33,62
sp|Q15149-4|PL	Plectin	PLEC		0,19	0,23	119	119	119	29,6	29,6	29,6	516,19	0	323,3	1,4E+10	426	Q15149-	Q15149-	31,16	30,88	31,46	30,76	32,18	31,24
sp|Q6Y1H2|HAC	Very-long-chain (3	HACD2		0,29	0,22	2	2	2	7,9	7,9	7,9	28,368	0	2,972	7E+07	4	Q6Y1H2	Q6Y1H2	23,89	24,27	23,97	24,82	23,92	24,05
sp|P00167-2|CY	Cytochrome b5	CYB5A		0,31	0,22	2	2	2	22,4	22,4	22,4	11,268	0	3,491	1,2E+08	5	P00167-	P00167-	24,19	24,75	24,98	25,09	24,53	24,95
sp|P14625|ENPI	Endoplasmin	HSP90B1		0,19	0,22	7	5	5	8,6	6,8	6,8	92,468	0	10,15	2,5E+08	9	P14625;	P14625	25,45	25,50	25,46	26,55	25,21	25,30
sp|P61019|RAB	Ras-related protei	RAB2A;RAB2B		0,57	0,21	6	6	6	36,3	36,3	36,3	23,545	0	22,79	8,5E+08	18	P61019;	P61019;	27,40	27,14	27,36	27,74	27,25	27,55
tr|A0A0U1RQF0	Fatty acid synthas	FASN		0,62	0,21	37	37	37	22	22	22	273,2	0	249,4	6,4E+09	142	A0A0U1	A0A0U1	30,23	30,04	30,49	30,57	30,30	30,52
sp|P68363|TBA	Tubulin alpha-1B	TUBA1B;TUBA1C;TUB		0,88	0,21	19	19	1	51,9	51,9	3,1	50,151	0	323,3	8E+10	290	P68363;	P68363;	33,89	33,79	33,91	34,16	33,87	34,20
sp|O75821|EIF3	Eukaryotic translat	EIF3G		0,48	0,21	3	3	3	11,9	11,9	11,9	35,611	0	7,405	3E+08	12	O75821;	O75821;	25,75	25,95	25,49	26,12	25,67	26,03
sp|P84077|ARF	ADP-ribosylation f	ARF1;ARF3;ARF5		0,64	0,20	7	7	4	54,7	54,7	26	20,697	0	38,78	2,2E+09	43	P84077;	P84077;	28,73	28,52	28,91	29,10	28,80	28,88
sp|P31946-2|14	14-3-3 protein beta	YWHAB		0,19	0,20	8	5	4	36,1	25,8	22,1	27,85	0	51,74	5,7E+08	16	P31946-	P31946-	26,63	26,49	27,15	27,67	26,54	26,65
sp|P51149|RAB	Ras-related protei	RAB7A		0,48	0,20	6	6	6	29,5	29,5	29,5	23,489	0	63,89	1,3E+09	33	P51149;	P51149;	27,93	27,81	28,16	28,40	27,89	28,20
tr|J3KPX7|J3KP	Prohibitin-2	PHB2		0,26	0,20	14	14	14	53	53	53	33,239	0	146,2	7,1E+09	111	J3KPX7	J3KPX7	30,29	30,19	30,71	31,03	30,13	30,63
sp|P26641|EF1(	Elongation factor 1	EEF1G		0,26	0,20	11	11	11	26,3	26,3	26,3	50,118	0	51,46	1,6E+09	65	P26641;	P26641;	28,14	28,04	28,65	28,93	28,37	28,13
sp|Q13148|TADI	TAR DNA-binding	TARDBP;TDP43		0,46	0,20	5	5	5	17,1	17,1	17,1	44,739	0	48,95	9,2E+08	31	Q13148;	Q13148;	27,54	27,53	27,22	27,60	27,38	27,89
sp|P04406|G3P_	Glyceraldehyde-3-	GAPDH		0,12	0,19	17	17	17	65,1	65,1	65,1	36,053	0	323,3	1,4E+10	140	P04406;	P04406;	30,85	31,33	31,46	32,44	31,32	30,46
sp|P31942-2|HN	Heterogeneous nu	HNRNPH3		0,37	0,19	3	3	3	10,6	10,6	10,6	35,238	0	8,458	4,3E+08	13	P31942-	P31942-	26,40	26,39	26,07	26,81	26,16	26,47
sp|P62424|RL7A	60S ribosomal pro	RPL7A		0,08	0,19	4	4	4	15	15	15	29,995	0	44,99	4,9E+08	11	P62424;	P62424;	26,23	25,99	25,99	25,03	27,94	25,82
sp|P35232|PHB_	Prohibitin	PHB		0,22	0,19	13	13	13	66,9	66,9	66,9	29,804	0	273,9	7,6E+09	110	P35232;	P35232;	30,45	30,40	30,67	31,26	30,14	30,69
tr|H0YHC3|H0YI	Nucleosome asser	NAP1L1		0,61	0,19	6	6	6	36,9	36,9	36,9	23,417	0	65,03	1,9E+09	35	H0YHC3	H0YHC3	28,62	28,30	28,58	28,83	28,51	28,72
sp|P49411|EFTU	Elongation factor T	TUFM		0,42	0,18	14	14	14	36,3	36,3	36,3	49,541	0	96,8	2,6E+09	83	P49411;	P49411	29,05	28,80	28,94	29,07	28,83	29,43
tr|A0A087WVQ6	Clathrin heavy cha	CLTC		0,57	0,18	23	23	23	16,8	16,8	16,8	192,06	0	172,9	3,3E+09	128	A0A087	A0A087	29,31	29,23	29,41	29,72	29,27	29,49
sp|P17655-2|CA	Calpain-2 catalytic	CAPN2		0,36	0,17	4	4	4	6,4	6,4	6,4	71,475	0	5,021	1,9E+08	6	P17655-	P17655-	25,09	25,23	25,33	25,62	25,51	25,03
tr|F5H6U7|F5H6	Vesicle transport p	GOLT1B		0,24	0,17	2	2	2	18,8	18,8	18,8	14,307	0	7,299	7,4E+07	2	F5H6U7	F5H6U7	23,78	23,79	23,77	24,50	23,65	23,71
tr|Q60FE5|Q60F	Filamin-A	FLNA		0,25	0,17	24	24	20	12,2	12,2	10,3	278,22	0	95,29	2,9E+09	84	Q60FE5	Q60FE5	28,95	29,05	29,46	29,75	29,07	29,14
sp|P43358|MAG	Melanoma-associa	MAGEA4		0,36	0,17	7	7	7	19,9	19,9	19,9	34,899	0	76,7	1,7E+09	60	P43358;	P43358	28,46	28,25	28,26	28,85	28,31	28,31
sp|Q13151|ROA	Heterogeneous nu	HNRNPA0		0,53	0,16	4	4	4	21	21	21	30,84	0	31,33	6,5E+08	21	Q13151	Q13151	27,17	26,71	26,90	27,05	27,16	27,06
sp|P52597|HNR	Heterogeneous nu	HNRNPF		0,33	0,16	8	8	7	25,1	25,1	22,7	45,671	0	74,69	2,7E+09	66	P52597;	P52597	29,06	28,81	29,14	29,32	28,81	29,37
tr|H0Y400|H0Y4	ATP-dependent R	DDX39B;DDX39A		0,24	0,16	3	3	3	19,3	19,3	19,3	21,679	0	21,46	3,5E+08	15	H0Y400;	H0Y400;	25,77	26,31	26,04	26,63	26,02	25,96
sp|P31947-2|14	14-3-3 protein sigr	SFN		0,29	0,16	10	10	7	45,8	45,8	34,3	24,336	0	80,42	4,3E+09	79	P31947-	P31947-	29,60	29,44	29,87	30,15	29,58	29,65
sp|Q9P0L0|VAP	Vesicle-associated	VAPA		0,25	0,15	1	1	1	6,4	6,4	6,4	27,893	0	10,04	2,2E+08	13	Q9P0L0;	Q9P0L0;	25,43	25,17	25,70	25,94	25,33	25,49
sp|P18124|RL7_	60S ribosomal pro	RPL7		0,06	0,15	4	4	4	20,2	20,2	20,2	29,225	0	19,56	7,7E+08	17	P18124;	P18124;	26,91	26,59	26,82	25,86	28,55	26,37
sp|O95373|IPO7	Importin-7	IPO7		0,17	0,15	4	4	4	5,1	5,1	5,1	119,52	0	18,13	4,3E+08	18	O95373;	O95373	26,27	25,90	26,73	26,90	26,11	26,33
sp|P05556-2|ITE	Integrin beta-1	ITGB1		0,34	0,15	3	3	3	5,6	5,6	5,6	87,445	0	6,538	2,2E+08	10	P05556-	P05556-	25,22	25,36	25,61	25,83	25,39	25,42
tr|D6REM6|D6R	Matrin-3	MATR3		0,33	0,14	14	14	14	26,7	26,7	26,7	88,358	0	195,6	2,8E+09	69	D6REM6	D6REM6	29,30	28,96	29,03	29,36	28,96	29,39
sp|P09496-5|CL	Clathrin light chair	CLTA		0,40	0,14	3	3	3	21,7	21,7	21,7	17,687	0	16,58	1,7E+08	7	P09496-	P09496-	24,92	25,00	25,01	25,29	24,83	25,23
sp|O75643|U52(	U5 small nuclear r	SNRNP200		0,20	0,14	4	4	4	3,1	3,1	3,1	244,5	0	12,6	2,7E+08	6	O75643;	O75643;	26,03	25,53	25,13	25,60	25,80	25,70
sp|Q06323|PSM	Proteasome activa	PSME1		0,35	0,13	6	6	6	32,1	32,1	32,1	28,723	0	16,17	4,8E+08	14	Q06323;	Q06323;	26,65	26,26	26,44	26,76	26,38	26,61
tr|A0A087WX59	Proteasomal ubiqu	ADRM1		0,45	0,13	1	1	1	3	3	3	37,504	0	3,083	4,6E+07	5	A0A087	A0A087	23,25	22,96	23,36	23,28	23,36	23,32
tr|H7C5F2|H7C5	28S ribosomal pro	MRPS22		0,18	0,12	2	2	2	17	17	17	18,573	0	4,563	1,3E+08	3	H7C5F2	H7C5F2	24,81	24,54	24,57	24,43	24,61	25,25
sp|Q14974|IMB1	Importin subunit b	KPNB1		0,23	0,12	15	15	15	23,3	23,3	23,3	97,169	0	113,8	3E+09	67	Q14974;	Q14974;	29,32	29,06	29,25	29,71	29,15	29,13
tr|E9PM69|E9PI	26S protease regu	PSMC3		0,30	0,12	3	3	3	11,6	11,6	11,6	44,323	0	11,02	3,2E+08	14	E9PM69	E9PM69	26,13	25,86	25,77	25,94	25,90	26,29
sp|Q15021|CND	Condensin comple	NCAPD2		0,22	0,12	3	3	3	3,4	3,4	3,4	157,18	0	6,655	1,7E+08	7	Q15021;	Q15021	25,07	25,13	24,87	25,47	24,80	25,15
sp|P18859|ATP	ATP synthase-cou	ATP5J		0,17	0,12	3	3	3	44,4	44,4	44,4	12,587	0	12,58	3,5E+08	12	P18859;	P18859;	26,10	25,97	26,39	26,72	26,00	26,08
sp|O75477|ERLI	Erlin-1;Erlin-2	ERLIN1;ERLIN2		0,14	0,11	3	3	3	10,9	10,9	10,9	39,171	0	20,71	4,4E+08	20	O75477;	O75477;	26,38	26,09	26,99	26,78	26,38	26,63
sp|P40227|TCP2	T-complex protein	CCT6A		0,22	0,11	6	6	6	16,6	16,6	16,6	58,024	0	121,9	1,4E+09	48	P40227;	P40227;	28,06	28,08	28,27	28,56	27,96	28,21
tr|A0A087WXM6	60S ribosomal pro	RPL17;RPL17-C18orf32		0,06	0,10	3	3	3	23,1	23,1	23,1	19,586	0	10,73	4E+08	10	A0A087	A0A087	26,29	25,92	25,98	25,39	27,29	25,82
sp|P50991-2|TC	T-complex protein	CCT4		0,24	0,10	8	8	8	18,7	18,7	18,7	54,719	0	21,26	1,1E+09	39	P50991-	P50991-	27,80	27,54	27,80	28,10	27,63	27,71
sp|P08238|HS9(	Heat shock protein	HSP90AB1		0,18	0,10	27	27	12	39,4	39,4	18,9	83,263	0	323,3	1,8E+10	242	P08238;	P08238	31,75	31,70	31,97	32,27	31,62	31,82
sp|O00264|PGR	Membrane-associa	PGRMC1		0,12	0,09	3	3	3	11,8	11,8	11,8	21,671	0	20,61	7,5E+08	24	O00264;	O00264	27,21	27,01	27,39	27,81	27,01	27,07
sp|P13639|EF2_	Elongation factor 2	EEF2		0,19	0,09	19	19	18	24,5	24,5	23,3	95,337	0	128	3,7E+09	91	P13639;	P13639	29,45	29,53	29,69	29,97	29,40	29,57
sp|P61978-3|HN	Heterogeneous nu	HNRNPK		0,16	0,09	15	15	15	40,9	40,9	40,9	48,562	0	235,7	6,6E+09	102	P61978-	P61978-	30,50	30,14	30,34	30,74	30,12	30,39
sp|P36542-2|AT	ATP synthase sub	ATP5C1		0,10	0,09	6	6	6	19,9	19,9	19,9	32,881	0	27,32	1,1E+09	39	P36542-	P36542-	27,60	27,38	28,26	28,23	27,57	27,70
sp|Q9BUF5|TBE	Tubulin beta-6 cha	TUBB6		0,22	0,09	17	10	7	52,7	39	30,3	49,857	0	251,8	6E+09	81	Q9BUF5	Q9BUF5	30,34	30,22	30,07	30,28	30,07	30,53
tr|B4DY09|B4DY	Interleukin enhanc	ILF2		0,27	0,09	4	4	4	15,3	15,3	15,3	38,91	0	17	2,9E+08	17	B4DY09	B4DY09	26,03	25,80	25,70	26,08	25,88	25,82
tr|A0A0A0MRM9	Nucleolar and coile	NOLC1		0,18	0,08	10	10	10	14,4	14,4	14,4	74,543	0	35,26	2,6E+09	73	A0A0A0	A0A0A0	29,03	28,91	28,98	29,33	28,72	29,13
tr|F5GXX5|F5G)	Dolichyl-diphosph	DAD1		0,09	0,08	3	3	3	37,6	37,6	37,6	9,5541	0	12,07	3,9E+08	13	F5GXX5	F5GXX5	25,98	26,18	26,67	26,68	25,90	26,50
tr|A0A0C4DGH5	Cullin-associated	CAND1		0,21	0,08	4	4	4	6,8	6,8	6,8	85,408	0	13,84	3E+08	17	A0A0C4	A0A0C4	26,02	25,79	25,68	26,12	25,78	25,83
sp|O14929-2|HA	Histone acetyltrans	HAT1		0,19	0,08	3	3	3	16,8	16,8	16,8	39,786	0	24,8	4,6E+08	11	O14929-	O14929-	26,67	26,40	26,36	26,70	26,31	26,67
sp|P22102-2|PU	Trifunctional purin	GART		0,18	0,07	5	5	5	13,4	13,4	13,4	46,033	0	9,216	5,1E+08	20	P22102-	P22102-	26,87	26,55	26,55	26,80	26,50	26,89
tr|F6VRR5|F6VF	Polymerase delta-i	POLDIP3		0,28	0,07	3	3	3	10	10	10	48,103	0	6,007	2,5E+08	4	F6VRR5	F6VRR5	25,70	25,48	25,63	25,52	25,74	25,76
sp|P62805|H4_H	Histone H4	HIST1H4A		0,05	0,07	11	11	11	61,2	61,2	61,2	11,367	0	110,7	9,5E+10	160	P62805	P62805	34,36	33,92	33,79	33,37	34,85	34,06
sp|Q15366-6|PC	Poly(rC)-binding p	PCBP2		0,13	0,07	8	8	5	31,6	31,6	21,6	38,15	0	85,83	2,1E+09	53	Q15366-	Q15366-	28,83	28,54	28,66	28,81	28,42	28,99
sp|P22314-2|UB	Ubiquitin-like modi	UBA1		0,07	0,06	4	4	4	6,8	6,8	6,8	113,8	0	68,63	3,6E+08	20	P22314-	P22314-	26,06	26,04	26,28	26,79	26,00	25,78
sp|Q15907|RB1	Ras-related protei	RAB11B;RAB11A		0,06	0,06	9	9	9	43,1	43,1	43,1	24,488	0	21,71	1,6E+09	33	Q15907;	Q15907;	28,39	28,07	28,60	29,01	27,82	28,41
tr|M0R3D6|M0R	60S ribosomal pro	RPL18A		0,02	0,06	3	3	3	17	17	17	16,714	0	4,313	2,6E+08	6	M0R3D6	M0R3D6	25,45	25,18	25,13	23,79	27,04	25,10
sp|P27824|CAL	Calnexin	CANX		0,08	0,06	9	9	9	16,9	16,9	16,9	67,567	0	29,74	9,2E+08	39	P27824;	P27824;	27,47	27,38	27,71	27,97	27,24	27,51
sp|P37108|SRP	Signal recognition	SRP14		0,10	0,06	3	3	3	27,2	27,2	27,2	14,57	0	29,04	7,6E+08	18	P37108;	P37108;	27,51	27,15	26,98	27,26	27,06	27,50
sp|P16403|H12_	Histone H1.2;Hist	HIST1H1C;HIST1H1D		0,03	0,05	9	9	2	31,9	31,9	11,7	21,364	0	29,08	5,4E+09	71	P16403;	P16403;	30,45	29,43	29,58	29,05	30,88	29,69
tr|E5RJR5|E5RJ	S-phase kinase-as	SKP1		0,16	0,05	3	3	3	16	16	16	18,72	0	20,59	8,2E+08	14	E5RJR5	E5RJR5	27,51	27,30	27,15	27,49	27,32	27,30
sp|O14980|XPO	Exportin-1	XPO1		0,11	0,05	6	6	6	5,8	5,8	5,8	123,38	0	13,67	3,2E+08	17	O14980;	O14980	26,06	26,02	25,68	26,13	25,87	25,90
sp|P07814|SYEI	Bifunctional glutan	EPRS		0,08	0,04	5	5	5	5,4	5,4	5,4	170,59	0	13,65	4,2E+08	15	P07814;	P07814	26,51	26,47	26,12	26,67	26,33	26,23
sp|P68371|TBB	Tubulin beta-4B c	TUBB4B		0,10	0,04	19	5	1	51,9	19,3	2,7	49,83	0	166,5	1E+10	66	P68371;	P68371	31,19	30,82	30,74	30,87	30,95	31,04
sp|P68431|H31_	Histone H3.1;Hist	HIST1H3A;HIST2H3A;H		0,04	0,03	6	6	2	52,9	52,9	28,7	15,404	0	14,14	1,6E+10	43	P68431;	P68431;	31,74	31,45	31,48	31,20	32,13	31,44
tr|D6RAA6|D6R	Transmembrane p	TMEM33		0,04	0,03	2	2	2	9,9	9,9	9,9	25,223	0	20,35	4,2E+08	20	D6RAA6	D6RAA6	26,51	26,22	26,49	26,80	25,99	26,53
sp|P10809|CH6(	60 kDa heat shock	HSPD1		0,05	0,03	24	24	24	48,3	48,3	48,3	61,054	0	323,3	1,3E+10	190	P10809;	P10809	31,36	31,18	31,26	31,68	31,02	31,19
tr|Q5QNZ2|Q5Q	ATP synthase F(0	ATP5F1		0,02	0,03	4	4	4	22,1	22,1	22,1	22,275	0	45,9	7,1E+08	25	Q5QNZ2	Q5QNZ2	27,10	26,83	27,63	27,90	26,69	27,06
sp|P23528|COF	Cofilin-1	CFL1		0,06	0,03	10	10	10	54,2	54,2	54,2	18,502	0	168,9	6,6E+09	62	P23528;	P23528;	30,38	30,34	30,40	30,68	30,13	30,39
sp|P20700|LMNI	Lamin-B1	LMNB1		0,05	0,03	22	22	19	40,8	40,8	37,9	66,408	0	180,8	4,6E+09	92	P20700;	P20700;	29,87	29,61	30,09	30,05	29,90	29,72
tr|V9GYY3|V9G	C-1-tetrahydrofolat	MTHFD1		0,04	0,02	6	6	6	7,2	7,2	7,2	99,719	0	18,67	6,7E+08	26	V9GYY3	V9GYY3	26,96	27,00	27,39	27,45	26,88	27,10
tr|D6RAF8|D6R	Heterogeneous nu	HNRNPD		0,03	0,02	5	5	4	28,1	28,1	24,4	23,076	0	49,58	8,1E+08	33	D6RAF8	D6RAF8	27,40	27,43	27,17	27,81	26,98	27,27
sp|P60842|IF4A	Eukaryotic initiatio	EIF4A1;EIF4A2		0,04	0,02	6	6	6	17,2	17,2	17,2	46,153	0	29,53	8,2E+08	37	P60842;	P60842;	27,41	27,34	27,42	27,72	27,16	27,36
tr|E9PHS0|E9PI	LanC-like protein 1	LANCL1		0,03	0,02	2	2	2	12,8	12,8	12,8	22,042	0	4,986	2,1E+08	5	E9PHS0	E9PHS0	25,35	25,24	25,83	25,43	25,39	25,66
tr|E7EP32|E7EF	Guanine nucleotid	GNB2;GNB1;GNB4		0,04	0,02	2	2	2	7,1	7,1	7,1	32,408	0	4,389	2,5E+08	12	E7EP32	E7EP32	25,57	25,48	25,81	25,58	25,82	25,51
sp|P23526|SAHI	Adenosylhomocys	AHCY		0,01	0,01	3	3	3	7,6	7,6	7,6	47,716	0	7,269	2,1E+08	11	P23526;	P23526;	25,53	25,46	25,12	25,53	25,07	25,54
sp|P06733|ENO	Alpha-enolase	ENO1		0,00	0,00	15	15	15	45,9	45,9	45,9	47,168	0	200,9	1,8E+09	49	P06733;	P06733;	27,93	28,18	28,78	29,83	27,89	27,18
sp|P55084-2|EC	Trifunctional enzyr	HADHB		0,00	0,00	8	8	8	29	29	29	48,879	0	101,6	1,3E+09	25	P55084-	P55084-	28,21	28,13	27,91	28,31	27,73	28,20
sp|P45974-2|UB	Ubiquitin carboxyl-	USP5		0,00	0,00	5	5	5	7,5	7,5	7,5	93,307	0	11,2	3,6E+08	15	P45974-	P45974-	26,28	26,24	26,16	26,54	26,06	26,07
sp|P68133|ACT	Actin, alpha skelet	ACTA1;ACTC1;ACTG2;		0,00	0,00	12	1	1	29,2	4,2	4,2	42,051	0	45,77	5,4E+09	30	P68133;	P68133;	29,84	29,70	29,96	30,17	29,85	29,48
sp|P00387-2|NB	NADH-cytochrome	CYB5R3		0,02	-0,02	4	4	4	18,7	18,7	18,7	31,628	0	22,05	5,6E+08	22	P00387-	P00387-	26,99	26,60	26,91	27,29	26,56	26,60
sp|P62316|SMD	Small nuclear ribo	SNRPD2		0,03	-0,02	3	3	3	22	22	22	13,527	0	4,14	2,5E+08	12	P62316;	P62316;	25,72	25,56	25,92	25,95	25,36	25,85
sp|P62829|RL23	60S ribosomal pro	RPL23		0,03	-0,02	2	2	2	17,9	17,9	17,9	14,865	0	32,12	5,4E+08	9	P62829;	P62829;	26,83	26,71	26,56	26,38	26,95	26,72
tr|H3BNX8|H3BI	Cytochrome c oxic	COX5A		0,01	-0,02	2	2	2	15,7	15,7	15,7	17,235	0	3,643	1,6E+08	3	H3BNX8	H3BNX8	24,64	24,56	24,72	26,19	22,83	24,84
sp|P04843|RPN	Dolichyl-diphosph	RPN1		0,03	-0,02	13	13	13	31,1	31,1	31,1	68,569	0	96,95	2,2E+09	49	P04843;	P04843;	28,84	28,53	29,25	29,14	28,69	28,72
tr|H7BZ94|H7BZ	Protein disulfide-is	P4HB		0,04	-0,02	4	4	4	9,1	9,1	9,1	52,502	0	41,22	2,8E+08	12	H7BZ94	H7BZ94	25,70	25,80	25,90	26,18	25,58	25,57
tr|C9JU14|C9JU	Ras-related protei	RAB6B;RAB6A		0,03	-0,02	3	3	3	36,1	36,1	36,1	9,6668	0	12,33	1,1E+09	14	C9JU14;	C9JU14;	27,82	27,60	27,93	28,30	27,28	27,70
sp|P56385|ATP	ATP synthase sub	ATP5I		0,02	-0,02	2	2	2	30,4	30,4	30,4	7,9331	0	3,788	2,4E+08	8	P56385	P56385	25,49	25,18	26,21	26,18	25,39	25,24
sp|P48047|ATP(	ATP synthase sub	ATP5O		0,03	-0,03	5	5	5	27,2	27,2	27,2	23,277	0	21,71	1,2E+09	27	P48047;	P48047;	27,94	27,62	28,38	28,46	27,68	27,72
sp|P68104|EF1A	Elongation factor 1	EEF1A1;EEF1A1P5		0,06	-0,03	15	15	7	48,3	48,3	27,3	50,14	0	216	6,4E+10	201	P68104;	P68104;	33,73	33,53	33,76	33,70	33,41	33,83
sp|P36578|RL4_	60S ribosomal pro	RPL4		0,01	-0,03	9	9	9	25,3	25,3	25,3	47,697	0	62,36	1,5E+09	26	P36578;	P36578;	28,24	27,51	27,83	26,43	29,32	27,75
sp|P26599|PTBI	Polypyrimidine tra	PTBP1		0,06	-0,03	12	12	12	37,5	37,5	37,5	57,221	0	93,58	3,3E+09	68	P26599;	P26599;	29,53	29,38	29,32	29,54	29,06	29,54

sp|P0DP25|CALM3_HUMAN Calmo		CALM2;CALM3;CALM1		0,04	-0,03	5	5	5	41,6	41,6	41,6	16,837	0	46,35	1,6E+09	34	P0DP25	P0DP25;	28,21	28,52	28,15	28,52	27,80	28,47
sp|Q00839|HNR	Heterogeneous nu	HNRNPU		0,05	-0,03	18	18	1	27	27	2,4	90,583	0	313,9	7,5E+09	142	Q00839	Q00839	30,90	30,55	30,19	30,69	30,40	30,45
tr|F8W1R7|F8W	Myosin light polype	MYL6		0,06	-0,04	6	6	6	46,9	46,9	46,9	16,29	0	14,62	5,5E+08	28	F8W1R7	F8W1R7	26,88	26,69	27,16	27,14	26,57	26,91
sp|P06576|ATPE	ATP synthase sub	ATP5B		0,04	-0,04	15	15	15	46,9	46,9	46,9	56,559	0	187,1	7E+09	107	P06576;	P06576;	30,36	30,28	30,86	30,96	30,05	30,38
sp|P51571|SSRI	Translocon-associ	SSR4		0,10	-0,04	2	2	2	18,5	18,5	18,5	18,998	0	14,82	4,9E+08	16	P51571;	P51571;	26,89	26,55	26,48	26,76	26,56	26,47
sp|Q00059-2|TF	Transcription facto	TFAM		0,06	-0,04	2	2	2	12,1	12,1	12,1	25,465	0	6,077	2,3E+08	6	Q00059-	Q00059-	25,92	25,42	25,17	25,26	25,39	25,73
sp|Q07065|CKAI	Cytoskeleton-asso	CKAP4		0,04	-0,04	10	10	10	22,1	22,1	22,1	66,022	0	47,19	8,3E+08	32	Q07065	Q07065	27,36	26,99	27,93	27,97	27,11	27,08
sp|O60664-4|PL	Perilipin-3	PLIN3		0,11	-0,05	3	3	3	11,4	11,4	11,4	45,803	0	28,65	3,4E+08	14	O60664-	O60664-	26,09	26,10	26,21	26,39	25,82	26,04
sp|P07384|CAN	Calpain-1 catalytic	CAPN1		0,12	-0,05	3	3	3	4,8	4,8	4,8	81,889	0	16,3	2,5E+08	12	P07384;	P07384	25,76	25,51	25,93	25,75	25,81	25,49
sp|P04844-2|RP	Dolichyl-diphospho	RPN2		0,08	-0,05	8	8	8	21,1	21,1	21,1	67,723	0	152,2	2,3E+09	47	P04844-	P04844-	28,74	28,76	29,13	29,20	28,72	28,55
sp|Q9NS86|LAN	LanC-like protein 2	LANCL2		0,07	-0,06	3	3	3	10	10	10	50,854	0	28,09	9E+08	23	Q9NS86	Q9NS86	27,92	27,27	27,31	27,34	27,22	27,78
tr|E9PF10|E9PF	Nuclear pore comp	NUP155		0,12	-0,06	5	5	5	4,1	4,1	4,1	148,09	0	11,85	3,2E+08	10	E9PF10;	E9PF10;	26,33	25,98	25,76	25,94	26,08	25,88
sp|P17987|TCP/	T-complex protein	TCP1		0,15	-0,06	8	8	8	18,7	18,7	18,7	60,343	0	27,57	9,7E+08	22	P17987;	P17987;	27,78	27,61	27,57	27,78	27,30	27,68
sp|Q9UDW1|QC	Cytochrome b-c1 c	UQCR10		0,10	-0,07	2	2	2	38,1	38,1	38,1	7,3084	0	11,76	3,7E+08	18	Q9UDW	Q9UDW	26,51	25,88	26,51	26,43	26,08	26,19
sp|Q15758|AAA	Neutral amino acic	SLC1A5		0,21	-0,07	2	2	2	5,7	5,7	5,7	56,598	0	7,576	1,5E+08	6	Q15758;	Q15758;	25,08	24,83	24,87	25,04	24,86	24,68
sp|Q03252|LMNI	Lamin-B2	LMNB2		0,12	-0,07	9	6	6	12,7	10	10	69,948	0	16,56	3E+08	22	Q03252	Q03252	26,01	25,64	26,29	26,10	25,88	25,74
sp|Q13185|CBX:	Chromobox proteir	CBX3		0,08	-0,08	3	3	3	23,5	23,5	23,5	20,811	0	30,51	3,9E+08	16	Q13185;	Q13185	26,64	26,02	26,23	25,73	26,67	26,27
sp|Q9P258|RCC	Protein RCC2	RCC2		0,14	-0,08	12	12	12	32,6	32,6	32,6	56,084	0	123,9	3,3E+09	70	Q9P258	Q9P258	29,69	29,32	29,24	29,19	29,17	29,65
sp|P05388|RLA(	60S acidic riboson	RPLP0;RPLP0P6		0,04	-0,09	4	4	4	21,1	21,1	21,1	34,273	0	38,39	7E+08	15	P05388;	P05388;	27,42	26,68	26,47	25,99	28,03	26,29
sp|P25705|ATP/	ATP synthase sub	ATP5A1		0,09	-0,09	23	23	23	45	45	45	59,75	0	283,4	1E+10	158	P25705;	P25705;	30,86	30,76	31,50	31,43	30,67	30,75
tr|U3KQE2|U3K(	Calpain small subu	CAPNS1;CAPNS2		0,14	-0,09	2	2	2	13,7	13,7	13,7	18,445	0	6,553	2,3E+08	12	U3KQE2	U3KQE2	25,53	25,50	25,97	25,76	25,22	25,75
sp|Q96FQ6|S10	Protein S100-A16	S100A16		0,61	-0,10	3	3	3	33	33	33	11,801	0	13,04	3,1E+08	18	Q96FQ6	Q96FQ6	26,04	25,86	26,05	25,93	25,91	25,82
sp|Q09666|AHN	Neuroblast differer	AHNAK		0,44	-0,10	50	50	50	20,5	20,5	20,5	629,09	0	213,1	9E+09	204	Q09666;	Q09666	30,87	30,75	31,03	30,85	30,69	30,82
tr|Q5W0J0|Q5W	Ras-related proteir	RAB18		0,21	-0,10	2	2	2	16,1	16,1	16,1	18,023	0	3,501	1,8E+08	9	Q5W0J(	Q5W0J0	25,17	25,23	25,39	25,15	24,89	25,45
tr|C9JXB8|C9JX	60S ribosomal pro	RPL24		0,07	-0,11	4	4	4	32,2	32,2	32,2	14,369	0	9,152	4,9E+08	20	C9JXB8	C9JXB8;	26,79	26,31	26,60	25,57	27,47	26,33
tr|A0A087X2I1|A	26S protease regu	PSMC6		0,21	-0,11	2	2	2	6,7	6,7	6,7	45,796	0	15,44	2,4E+08	14	A0A087	A0A087X	25,43	25,71	25,88	25,66	25,25	25,78
sp|O75396|SC22	Vesicle-trafficking	SEC22B		0,18	-0,11	8	8	8	43,7	43,7	43,7	24,593	0	61,99	8,5E+08	35	O75396;	O75396	27,51	27,36	27,66	27,67	26,97	27,57
tr|A0A1B0GWA2	Alkyldihydroxyacet	AGPS		0,24	-0,11	3	3	3	8,5	8,5	8,5	60,807	0	9,853	2,2E+08	8	A0A1B0	A0A1B0	25,46	25,68	25,39	25,73	25,23	25,22
sp|P62913|RL11	60S ribosomal pro	RPL11		0,20	-0,12	4	4	4	23,6	23,6	23,6	20,252	0	28,97	1,3E+09	37	P62913;	P62913;	28,28	27,81	28,04	27,61	28,21	27,97
sp|P05023-3|AT	Sodium/potassium	ATP1A1;ATP1A3;ATP1/		0,13	-0,12	9	9	9	11,5	11,5	11,5	109,55	0	46,13	1,2E+09	25	P05023-	P05023-	27,78	27,93	28,08	28,40	27,30	27,73
sp|P17844|DDX!	Probable ATP-dep	DDX5		0,25	-0,13	24	24	19	34,4	34,4	25,9	69,147	0	140,5	1,2E+10	159	P17844;	P17844;	31,61	31,21	31,06	30,95	31,19	31,36
sp|Q07666-2|KH	KH domain-contair	KHDRBS1;KHDRBS2		0,23	-0,13	4	4	4	10,3	10,3	10,3	45,86	0	19,11	5,5E+08	20	Q07666-	Q07666-	27,13	26,75	26,75	27,08	26,48	26,69
sp|Q9Y490|TLN	Talin-1	TLN1		0,07	-0,13	3	3	3	1,6	1,6	1,6	269,76	0	9,762	1,5E+08	9	Q9Y490	Q9Y490	24,96	24,80	24,77	25,65	23,45	25,04
sp|Q15181|IPYF	Inorganic pyrophos	PPA1		0,17	-0,13	4	4	4	24,9	24,9	24,9	32,66	0	74,72	3,9E+08	11	Q15181;	Q15181;	26,54	26,39	26,03	26,70	25,89	25,96
sp|P62266|RS23	40S ribosomal pro	RPS23		0,13	-0,14	3	3	3	23,8	23,8	23,8	15,807	0	8,945	6,2E+08	13	P62266;	P62266;	27,39	26,77	26,62	26,29	27,28	26,79
tr|A0A3B3IRI2|A	CTP synthase 1;C	CTPS1		0,37	-0,14	10	10	10	28	28	28	63,723	0	70,16	1,8E+09	50	A0A3B3	A0A3B3	28,79	28,54	28,46	28,52	28,21	28,64
sp|P25205|MCM	DNA replication lic	MCM3		0,63	-0,15	10	10	10	17,1	17,1	17,1	90,98	0	48,34	1,3E+09	47	P25205;	P25205;	28,12	28,07	28,22	27,97	27,84	28,17
sp|P39023|RL3_	60S ribosomal pro	RPL3		0,05	-0,15	7	7	7	20,8	20,8	20,8	46,108	0	29,06	9,7E+08	17	P39023;	P39023;	28,18	26,58	26,61	25,70	28,60	26,65
sp|P46940|IQG/	Ras GTPase-activ	IQGAP1		0,22	-0,15	5	5	4	4,2	4,2	3,6	189,25	0	18,56	1,7E+08	9	P46940;	P46940;	25,28	24,94	25,20	25,45	24,90	24,63
sp|P35221|CTN/	Catenin alpha-1;C	CTNNA1;CTNNA3		0,51	-0,15	2	2	2	2,9	2,9	2,9	100,07	0,0013	2,292	5,7E+09	6	P35221;	P35221;	30,36	29,92	30,22	30,03	29,97	30,05
sp|Q16777|H2A2	Histone H2A type	HIST2H2AC;HIST2H2A/		0,15	-0,16	7	7	1	63,6	63,6	22,5	13,988	0	50,65	3,3E+10	63	Q16777;	Q16777;	32,90	32,34	32,68	31,94	33,16	32,34
sp|P51572|BAP:	B-cell receptor-ass	BCAP31		0,11	-0,16	4	4	4	16,7	16,7	16,7	27,991	0	13,4	3E+08	17	P51572;	P51572;	25,67	25,59	26,78	26,54	25,34	25,70
sp|P55265-5|DS	Double-stranded R	ADAR		0,48	-0,16	4	4	4	5,3	5,3	5,3	103,64	0	9,528	1,8E+08	5	P55265-	P55265-	25,47	25,05	25,17	25,15	24,94	25,13
tr|H0YFD6|H0YF	Trifunctional enzyr	HADHA		0,49	-0,16	9	9	9	13,6	13,6	13,6	86,371	0	18,59	1E+09	29	H0YFD6	H0YFD6	27,70	27,84	27,85	27,84	27,37	27,69
sp|P67809|YBO	Nuclease-sensitive	YBX1		0,38	-0,17	7	7	5	41	41	31,2	35,924	0	98,44	8,8E+08	37	P67809;	P67809;	27,81	27,65	27,21	27,34	27,46	27,38
sp|P55884|EIF3I	Eukaryotic translat	EIF3B		0,22	-0,17	2	2	2	2,8	2,8	2,8	92,48	0	9,189	1,9E+08	18	P55884;	P55884;	25,31	25,52	25,30	25,80	24,87	24,96
sp|Q86X51|EZH	Uncharacterized p	CXorf67		0,31	-0,18	3	3	3	6,2	6,2	6,2	51,893	0	15,52	1,3E+08	9	Q86X51	Q86X51	24,85	24,66	24,73	24,82	24,12	24,77
tr|F8VZJ2|F8VZ	Nascent polypeptic	NACA		0,46	-0,18	4	4	4	40,4	40,4	40,4	15,016	0	45,07	7,2E+08	27	F8VZJ2;	F8VZJ2;	27,45	27,44	26,95	27,13	27,00	27,15
sp|P62937|PPIA	Peptidyl-prolyl cis-	PPIA		0,34	-0,19	8	8	8	35,8	35,8	35,8	18,012	0	75,69	5,9E+09	80	P62937;	P62937;	30,29	30,20	30,50	30,55	29,84	30,03
sp|P22626|ROA	Heterogeneous nu	HNRNPA2B1		0,24	-0,19	16	16	15	42,2	42,2	42,2	37,429	0	147,1	4,3E+09	110	P22626;	P22626;	29,99	29,68	29,86	30,16	29,67	29,13
tr|A0A0C4DGS1	Dolichyl-diphospho	DDOST		0,30	-0,20	5	5	5	16,2	16,2	16,2	48,799	0	94,24	1E+09	17	A0A0C4	A0A0C4	27,82	27,47	28,31	27,89	27,44	27,66
sp|P51991|ROA	Heterogeneous nu	HNRNPA3		0,39	-0,20	7	7	7	31,2	31,2	31,2	39,594	0	103,4	1,2E+09	40	P51991;	P51991;	28,45	27,91	27,75	27,96	27,71	27,83
sp|P61026|RAB	Ras-related proteir	RAB10		0,23	-0,20	4	3	3	20,5	15	15	22,541	0	13,53	4,8E+08	4	P61026	P61026	26,54	26,37	27,17	26,97	26,18	26,33
sp|P55060-4|XP	Exportin-2	CSE1L		0,44	-0,21	14	14	14	18,6	18,6	18,6	103,88	0	199,4	1,3E+10	62	P55060-	P55060-	31,38	31,34	31,24	31,49	31,02	30,83
tr|A0A2R8Y811|	40S ribosomal pro	RPS14		0,31	-0,21	4	4	4	36,7	36,7	36,7	16,159	0	18,52	7,9E+08	15	A0A2R8	A0A2R8	27,53	27,18	27,34	26,71	27,58	27,14
tr|U3KQK0|U3K(	Histone H2B;Histo	HIST1H2BN;HIST1H2BI		0,16	-0,21	9	9	2	44	44	6	18,804	0	164,2	5,5E+10	79	U3KQK(	U3KQK0	33,66	33,16	32,96	32,33	33,88	32,94
sp|Q86UP2-2|K	Kinectin	KTN1		0,24	-0,21	10	10	10	9,2	9,2	9,2	149,61	0	36,93	6E+08	22	Q86UP2	Q86UP2	26,83	27,02	27,43	27,48	26,58	26,59
tr|C9J0J7|C9J0J	Profilin;Profilin-2	PFN2		0,70	-0,21	6	3	3	62,6	30,8	30,8	9,8402	0	5,891	4,5E+08	15	C9J0J7;	C9J0J7;	26,61	26,60	26,66	26,13	26,52	26,58
tr|E9PK01|E9PK	Elongation factor 1	EEF1D		0,34	-0,22	7	7	7	34,5	34,5	34,5	28,821	0	112,5	1,6E+09	42	E9PK01	E9PK01	28,56	28,26	28,64	28,66	27,82	28,32
sp|P33992|MCM	DNA replication lic	MCM5	+	1,31	-0,22	7	7	7	12,8	12,8	12,8	82,285	0	14,8	5,4E+08	23	P33992;	P33992;	26,80	26,92	27,03	26,64	26,67	26,77
tr|A0A087WW6	26S proteasome n	PSMD1		0,46	-0,22	2	2	2	3,7	3,7	3,7	105,85	0	2,873	1,5E+08	8	A0A087	A0A087	25,02	25,16	24,68	24,63	25,04	24,53
sp|Q96PK6|RBN	RNA-binding prote	RBM14		0,17	-0,22	8	8	8	16	16	16	69,491	0	45,26	1,2E+09	28	Q96PK6	Q96PK6	28,56	27,71	27,45	26,94	28,09	28,01
tr|A0A087WUK2	Heterogeneous nu	HNRNPDL		0,71	-0,24	5	4	4	14,6	12,4	12,4	40,04	0	10,29	2,5E+08	18	A0A087	A0A087	25,87	25,73	25,61	25,76	25,40	25,32
tr|A0A4W8ZXM2	Ig heavy chain V-I	IGHV3-72;IGHV3OR16-!		0,53	-0,24	2	2	2	17,8	17,8	17,8	11,167	0	8,5	1,7E+10	22	A0A4W8	A0A4W8	31,97	31,71	31,89	31,43	31,43	31,99
tr|B5MDF5|B5MI	GTP-binding nucle	RAN		0,64	-0,25	6	6	6	27,9	27,9	27,9	26,224	0	43,25	1,4E+09	37	B5MDF5	B5MDF5	28,34	28,36	28,14	28,35	27,85	27,90
sp|Q9BS26|ERF	Endoplasmic retic	ERP44		0,30	-0,25	6	6	6	16	16	16	46,971	0	24,57	9E+08	24	Q9BS26	Q9BS26	27,71	27,51	27,56	26,88	27,17	28,00
tr|A0A2R8Y5G6	ATP-dependent R	DDX3X;DDX3Y		0,83	-0,25	11	10	10	23,6	21,9	21,9	69,047	0	75,78	1,3E+09	47	A0A2R8	A0A2R8	28,37	28,06	27,95	27,77	27,97	27,90
tr|A0A1X7SBZ2|	Probable ATP-dep	DDX17		0,45	-0,25	11	6	6	18	10,8	10,8	80,253	0	28,54	1,4E+09	18	A0A1X7	A0A1X7	28,68	28,03	28,09	28,15	27,75	28,12
sp|Q16658|FSCI	Fascin	FSCN1		0,13	-0,26	2	2	2	5,3	5,3	5,3	54,529	0	16,41	1,5E+08	3	Q16658;	Q16658;	24,87	24,96	25,13	25,93	23,39	24,86
tr|C9J712|C9J71	Profilin-2;Profilin	PFN2		0,65	-0,26	6	6	3	52,7	52,7	20,9	9,7982	0	36,07	2,3E+09	37	C9J712;	C9J712;	29,12	28,91	28,90	28,51	28,60	29,05
tr|H0YNU5|H0YI	Bloom syndrome p	BLM		0,31	-0,26	1	1	1	1,8	1,8	1,8	144,47	0	2,441	1,5E+10	14	H0YNU5	H0YNU5	31,91	31,74	31,37	31,05	31,16	32,02
sp|P04792|HSPI	Heat shock proteir	HSPB1		0,84	-0,27	11	11	11	61,5	61,5	61,5	22,782	0	189,6	9,5E+09	93	P04792;	P04792	31,05	30,89	31,15	30,99	30,74	30,56
tr|F8W6I7|F8W6	Heterogeneous nu	HNRNPA1;HNRNPA1L2		0,32	-0,27	12	11	11	47,2	44,3	44,3	33,155	0	146	2,6E+09	69	F8W6I7;	F8W6I7;	29,18	28,90	29,29	29,45	28,73	28,37
tr|K7ELC7|K7EL	60S ribosomal pro	RPL27		0,14	-0,27	4	4	4	35,4	35,4	35,4	16,359	0	40,71	5,4E+08	12	K7ELC7	K7ELC7	26,99	26,33	26,48	25,30	27,66	26,03
sp|Q15365|PCBI	Poly(rC)-binding p	PCBP1		0,64	-0,27	7	4	4	25,6	17,7	17,7	37,497	0	25,44	1,5E+09	24	Q15365;	Q15365	28,59	28,07	28,43	28,25	27,85	28,17
sp|Q99497|PARI	Protein deglycase	PARK7		0,44	-0,28	2	2	2	21,7	21,7	21,7	19,891	0	19,01	5,4E+08	10	Q99497;	Q99497;	27,35	26,69	26,53	26,57	26,41	26,77
sp|P07355|ANX/	Annexin A2;Annex	ANXA2;AN	+	1,32	-0,30	24	24	24	57,8	57,8	57,8	38,604	0	323,3	4,4E+10	281	P07355;	P07355;	33,32	33,26	33,20	33,00	32,78	33,12
sp|Q96AG4|LRC	Leucine-rich repea	LRRC59		0,55	-0,30	5	5	5	18,9	18,9	18,9	34,93	0	30,35	1,4E+09	46	Q96AG4	Q96AG4	28,45	28,18	28,16	28,39	27,64	27,86
tr|H0Y8C6|H0Y8	Importin-5	IPO5		0,90	-0,30	5	5	5	5,3	5,3	5,3	123,75	0	12,33	2,9E+08	15	H0Y8C6	H0Y8C6	25,99	25,97	25,99	25,99	25,53	25,52
tr|E9PCY7|E9P(	Heterogeneous nu	HNRNPH1;HNRNPH2		0,88	-0,30	6	5	5	16,6	14,2	14,2	47,087	0	91,93	2,1E+09	43	E9PCY7	E9PCY7	28,99	28,75	28,96	28,81	28,33	28,65
sp|P05387|RLA2	60S acidic riboson	RPLP2		0,22	-0,31	4	4	4	69,6	69,6	69,6	11,665	0	56,26	1,2E+09	31	P05387;	P05387;	28,32	28,02	27,35	26,96	28,50	27,31
tr|F5GZS6|F5GZ	4F2 cell-surface ar	SLC3A2		1,11	-0,31	5	5	5	13	13	13	64,872	0	28,57	5,1E+08	15	F5GZS6	F5GZS6	26,72	26,87	27,04	26,54	26,42	26,74
sp|Q9H0U4|RAE	Ras-related proteir	RAB1B;RAB1C		0,53	-0,31	10	10	5	56,2	56,2	30,8	22,171	0	64,72	3E+09	60	Q9H0U4	Q9H0U4	29,43	29,21	29,68	29,52	28,76	29,10
tr|C9JPM4|C9JF	ADP-ribosylation fa	ARF4		0,50	-0,32	6	3	3	63	22	22	14,553	0	7,435	4,1E+08	13	C9JPM4	C9JPM4	26,42	26,11	27,01	26,28	26,03	26,28
sp|Q02878|RL6_	60S ribosomal pro	RPL6		0,18	-0,32	6	6	6	24,7	24,7	24,7	32,728	0	53,92	7,4E+08	12	Q02878;	Q02878;	27,40	26,53	27,53	26,01	28,02	26,46
sp|P09661|RU2/	U2 small nuclear r	SNRPA1		0,91	-0,32	18	18	18	61,2	61,2	61,2	28,415	0	323,3	8,5E+10	169	P09661;	P09661;	34,25	34,13	34,37	33,90	33,67	34,19
sp|Q92973-2|TN	Transportin-1	TNPO1		0,43	-0,33	4	4	4	5,2	5,2	5,2	101,31	0	12,18	2,6E+08	9	Q92973-	Q92973-	26,12	25,91	25,54	25,90	24,98	25,70
sp|Q15084-3|PD	Protein disulfide-is	PDIA6		1,04	-0,33	8	8	8	21,7	21,7	21,7	47,837	0	42,75	1E+09	35	Q15084-	Q15084-	28,01	27,60	28,04	27,59	27,47	27,60
sp|Q15417-3|CN	Calponin-3	CNN3		0,87	-0,33	5	5	5	21,9	21,9	21,9	31,38	0	20,45	5,1E+08	12	Q15417-	Q15417-	27,08	26,77	26,80	26,78	26,27	26,60
sp|Q9UNF1|MA(	Melanoma-associa	MAGED2		1,23	-0,33	10	10	10	25,9	25,9	25,9	64,953	0	95,99	9E+08	32	Q9UNF1	Q9UNF1	27,81	27,68	27,53	27,30	27,20	27,53
sp|P30050|RL12	60S ribosomal pro	RPL12		0,13	-0,34	2	2	2	18,8	18,8	18,8	17,818	0	30,6	3,3E+08	11	P30050;	P30050;	26,25	25,55	25,68	23,81	27,02	25,63
sp|P02786|TFR1	Transferrin recepto	TFRC		0,32	-0,34	4	4	4	6,7	6,7	6,7	84,87	0	14,85	3,6E+08	18	P02786;	P02786;	26,68	25,94	26,28	26,68	25,41	25,80
tr|I3L3P7|I3L3P7	40S ribosomal pro	RPS15A		0,23	-0,35	2	2	2	23	23	23	11,477	0	21,44	4E+08	12	I3L3P7;I	I3L3P7;I	26,33	26,14	26,47	24,97	27,03	25,90
sp|Q9Y2S7|PDII	Polymerase delta-i	POLDIP2		0,75	-0,37	6	6	6	19,8	19,8	19,8	42,033	0	26,46	5,1E+08	25	Q9Y2S7	Q9Y2S7	27,04	26,71	26,78	26,51	26,12	26,81
sp|P09382|LEG	Galectin-1	LGALS1		0,98	-0,37	5	5	5	38,5	38,5	38,5	14,716	0	10,3	3,6E+09	25	P09382;	P09382	29,65	29,71	29,74	29,45	28,99	29,55
tr|F6U211|F6U2	40S ribosomal pro	RPS10;RPS10-NUDT3		0,28	-0,37	4	4	4	23,8	23,8	23,8	19,868	0	9,95	1,9E+08	15	F6U211;	F6U211;	25,41	25,06	25,26	24,01	25,83	24,77
tr|J3QS39|J3QS	Ubiquitin-60S ribo	UBB;RPS27A;UBC;UBA		0,90	-0,37	3	3	3	36,6	36,6	36,6	10,469	0	30,79	1,1E+09	17	J3QS39;	J3QS39;	27,67	27,85	28,31	27,54	27,61	27,57
sp|P62854|RS26	40S ribosomal pro	RPS26;RPS26P11		0,41	-0,37	3	3	3	27	27	27	13,015	0	9,954	1,2E+09	27	P62854;	P62854;	28,58	28,14	27,37	27,37	27,89	27,72
sp|P60866|RS2(	40S ribosomal pro	RPS20		0,58	-0,38	3	3	3	22,7	22,7	22,7	13,373	0	16,08	3,8E+08	16	P60866;	P60866;	26,69	26,27	26,11	25,60	26,41	25,90
sp|P62753|RS6_	40S ribosomal pro	RPS6		0,22	-0,39	3	3	3	13,3	13,3	13,3	28,68	0	26,44	6,6E+08	13	P62753;	P62753;	27,38	26,90	26,74	25,68	27,89	26,28
sp|P32119|PRD	Peroxiredoxin-2	PRDX2	+	2,93	-0,40	8	7	7	32,8	27,3	27,3	21,892	0	20,4	2,8E+09	56	P32119;	P32119;	29,33	29,43	29,35	28,92	28,95	29,04
sp|P62701|RS4	40S ribosomal pro	RPS4X;RPS4Y1;RPS4Y		0,51	-0,40	7	7	7	28,1	28,1	28,1	29,597	0	17,89	1,1E+09	25	P62701;	P62701;	28,41	27,79	27,49	27,09	27,82	27,56
tr|A0A0U1RQH7	RNA-binding prote	RBM39		1,00	-0,41	6	6	6	28,2	28,2	28,2	26,698	0	19,83	5,5E+08	21	A0A0U1	A0A0U1	27,33	26,90	26,68	26,59	26,56	26,55
sp|P06702|S10A	Protein S100-A9	S100A9		0,35	-0,41	6	6	6	51,8	51,8	51,8	13,242	0	79,9	3,5E+09	56	P06702	P06702	29,41	28,96	30,52	28,97	29,51	29,18
tr|E9PKZ0|E9PK	60S ribosomal pro	RPL8		0,17	-0,41	3	3	3	17,1	17,1	17,1	22,389	0	9,947	2,5E+08	15	E9PKZ0	E9PKZ0	26,03	25,10	25,44	23,45	26,46	25,40
sp|P31151|S10A	Protein S100-A7;F	S100A7;S100A7A		0,13	-0,42	2	2	2	21,8	21,8	21,8	11,471	0	4,032	2,5E+08	3	P31151;	P31151;	25,03	23,40	27,28	24,44	25,03	25,00
tr|K7ESE8|K7ES	Bleomycin hydrola	BLMH		0,30	-0,42	4	4	4	19,5	19,5	19,5	26,738	0	11,32	4,7E+08	19	K7ESE8	K7ESE8	26,55	25,93	27,62	26,02	26,82	25,99
tr|H0Y9Y4|H0Y9	40S ribosomal pro	RPS3A		0,36	-0,42	4	4	4	15,4	15,4	15,4	23,528	0	18,54	3,7E+08	11	H0Y9Y4	H0Y9Y4	26,55	25,92	26,04	25,28	26,65	25,31
sp|P81605|DCD_	Dermcidin;Surviva	DCD		0,57	-0,43	4	4	4	35,5	35,5	35,5	11,284	0	15,06	1,5E+09	17	P81605;	P81605;	28,36	28,10	29,17	28,10	27,96	28,29
sp|P49458|SRP(	Signal recognition	SRP9;DKFZ	+	1,39	-0,43	2	2	2	22,1	22,1	22,1	10,112	0	10,72	4,2E+08	13	P49458;	P49458;	26,76	26,54	26,61	26,31	25,95	26,36
tr|B8ZZQ6|B8ZZ	Prothymosin alpha	PTMA		0,50	-0,43	3	3	3	35,5	35,5	35,5	11,758	0	32,03	7,1E+08	14	B8ZZQ6	B8ZZQ6	27,52	27,39	26,95	27,48	26,35	26,73
sp|Q00839-2|HN	Heterogeneous nu	HNRNPU		0,41	-0,44	18	1	1	27	1,9	1,9	88,979	0	4,798	5,3E+07	13	Q00839-	Q00839-	24,24	23,29	23,34	23,84	22,74	22,97
sp|Q7L1Q6-2|BZ	Basic leucine zipp	BZW1;BZW2		1,06	-0,45	2	2	2	4,8	4,8	4,8	40,538	0	3,144	1,2E+08	4	Q7L1Q6	Q7L1Q6	25,19	24,64	24,60	24,33	24,29	24,47
tr|E7END7|E7EI	Ras-related proteir	RAB1A		0,70	-0,46	9	4	4	63,6	34,1	34,1	19,018	0	57,26	5,4E+08	21	E7END7	E7END7	27,17	26,85	26,96	26,99	26,02	26,61
sp|P49915-2|GU	GMP synthase [glu	GMPS		0,45	-0,46	2	2	2	5,2	5,2	5,2	65,928	0	10,29	1,5E+08	3	P49915-	P49915-	25,91	24,46	24,74	24,59	24,62	24,51
sp|Q92572|AP3S	AP-3 complex sub	AP3S1	+	1,56	-0,47	2	2	2	13,5	13,5	13,5	21,732	0	3,319	1,1E+08	3	Q92572;	Q92572;	24,68	24,81	24,89	24,25	24,15	24,57
sp|Q99661-2|KIF	Kinesin-like proteir	KIF2C		1,17	-0,47	4	4	4	7,9	7,9	7,9	75,561	0	24,54	3,3E+08	15	Q99661-	Q99661-	26,26	26,08	26,48	26,06	25,54	25,79
tr|H0Y5B4|H0Y5	60S ribosomal pro	RPL36A;RPL36A-HNRN		0,50	-0,49	2	2	2	16,1	16,1	16,1	13,228	0	4,771	1E+08	3	H0Y5B4	H0Y5B4	24,98	24,32	24,19	23,88	24,66	23,47
sp|Q02539|H11_	Histone H1.1	HIST1H1A		0,22	-0,49	7	4	4	34,9	25,1	25,1	21,842	0	42,67	6,8E+08	29	Q02539;	Q02539	27,79	26,78	26,16	25,52	27,91	25,83
sp|P38159|RBM	RNA-binding motif	RBMX;RBMXL1		0,57	-0,50	13	13	13	36,3	36,3	36,3	42,331	0	51,84	2,5E+09	72	P38159;	P38159;	29,54	28,97	28,73	28,36	29,19	28,18
tr|B1AK87|B1AK	F-actin-capping pr	CAPZB	+	2,00	-0,51	2	2	2	6,9	6,9	6,9	29,295	0	3,174	3,8E+08	5	B1AK87	B1AK87	26,69	26,54	26,36	26,07	25,91	26,09
sp|P52907|CAZ/	F-actin-capping pr	CAPZA1	+	1,65	-0,51	6	6	5	32,5	32,5	29	32,922	0	62,13	1,5E+09	39	P52907;	P52907	28,68	28,45	28,33	27,85	27,91	28,17
tr|F8W914|F8W	Reticulon;Reticulo	RTN4		0,71	-0,51	2	2	2	13,9	13,9	13,9	37,144	0	27,88	5,8E+08	13	F8W914	F8W914	27,22	27,08	27,01	27,18	26,07	26,52
sp|P61106|RAB	Ras-related proteir	RAB14		0,88	-0,52	5	5	5	33,5	33,5	33,5	23,897	0	22,93	4,1E+08	14	P61106;	P61106;	26,86	26,64	26,29	25,62	26,27	26,32
tr|Q5T7C4|Q5T7	High mobility grou	HMGB1;HMGB1P1		0,80	-0,53	2	2	2	20,9	20,9	20,9	18,311	0	10,23	6,5E+08	5	Q5T7C4	Q5T7C4	27,15	27,79	26,83	26,94	26,50	26,73
tr|H7C2W9|H7C	60S ribosomal pro	RPL31		0,19	-0,54	3	3	3	29,6	29,6	29,6	12,701	0	10,65	4,6E+08	7	H7C2W9	H7C2W9	26,89	26,22	26,13	23,78	27,42	26,41
sp|P35579|MYH	Myosin-9	MYH9		1,23	-0,54	22	22	21	14,9	14,9	14,4	226,53	0	211,3	2,8E+09	110	P35579;	P35579;	29,32	29,25	29,66	29,15	28,88	28,57
sp|O00303|EIF3	Eukaryotic translat	EIF3F		1,04	-0,55	4	4	4	12,9	12,9	12,9	37,563	0	11,67	3,5E+08	10	O00303;	O00303;	26,52	26,33	26,17	26,14	25,37	25,86
sp|P62269|RS18	40S ribosomal pro	RPS18		0,50	-0,56	5	5	5	25	25	25	17,718	0	12,83	8,3E+08	21	P62269;	P62269	27,62	27,37	27,46	26,07	27,74	26,96
sp|P62851|RS25	40S ribosomal pro	RPS25		0,56	-0,56	4	4	4	28	28	28	13,742	0	8,857	9,4E+08	19	P62851	P62851	27,96	27,70	27,54	26,41	27,89	27,22
sp|Q8TCJ2|STT	Dolichyl-diphospho	STT3B		0,88	-0,58	3	3	3	3,9	3,9	3,9	93,673	0	6,445	2,3E+08	7	Q8TCJ2	Q8TCJ2	26,00	25,23	26,21	25,23	25,35	25,11
tr|A0A0A0MS08	Ig gamma-1 chain	IGHG1	+	3,34	-0,62	3	3	3	10,5	10,5	10,5	43,911	0	31,66	2,6E+08	8	A0A0A0	A0A0A0	26,11	26,04	26,05	25,55	25,43	25,37
sp|P04083|ANX/	Annexin A1;Annex	ANXA1		0,79	-0,63	8	8	8	26,6	26,6	26,6	38,714	0	107	9,8E+08	21	P04083;	P04083;	27,66	27,84	28,25	27,91	27,15	26,82
tr|A0A075B6E2|	40S ribosomal pro	RPS19		0,37	-0,64	6	6	6	45,1	45,1	45,1	7,8309	0	10,97	3,6E+08	21	A0A075	A0A075	26,43	26,30	26,02	24,50	26,94	25,39
tr|E7EVA0|E7E\	Microtubule-assoc	MAP4	+	1,36	-0,64	5	5	5	2,7	2,7	2,7	245,44	0	9,691	2,4E+08	11	E7EVA0	E7EVA0	26,24	25,88	25,78	25,62	25,02	25,34
sp|P19338|NUCI	Nucleolin	NCL		0,74	-0,66	24	24	24	34,2	34,2	34,2	76,613	0	256,2	7E+09	138	P19338;	P19338;	31,02	30,84	30,03	29,62	30,50	29,79
sp|P31944|CASI	Caspase-14;Caspa	CASP14		0,73	-0,66	6	6	6	24,4	24,4	24,4	27,679	0	19,94	5,5E+08	24	P31944	P31944	26,70	27,00	27,62	25,90	26,97	26,48
sp|Q96HJ9-2|FN	Putative RNA-binc	LUC7L2;LUC7L		0,81	-0,66	3	3	1	7,2	7,2	3,3	54,223	0	8,791	2,1E+08	6	Q96HJ9-	Q96HJ9-	26,26	25,68	25,04	25,25	24,83	24,90

tr|F6S8N6|F6S8	Protein-L-isoaspar	PCMT1	+	1,42	-0,67	4	4	4	31,2	31,2	31,2	26,626	0	67,41	8,8E+08	18	F6S8N6;	F6S8N6;	28,02	27,78	27,36	27,14	27,17	26,83
sp|P29508|SPB3	Serpin B3	SERPINB3		0,57	-0,69	9	9	3	24,9	24,9	11	44,564	0	26,8	7,6E+08	27	P29508;	P29508;	27,29	26,99	28,49	26,49	27,45	26,76
sp|Q96H79|ZCC	Zinc finger CCCH-	ZC3HAV1L	+	1,59	-0,69	4	4	4	16,7	16,7	16,7	32,962	0	15,37	3,9E+08	19	Q96H79	Q96H79	26,90	26,55	26,24	25,99	25,82	25,80
sp|Q9HA64|KT3	Ketosamine-3-kina	FN3KRP	+	1,85	-0,71	6	6	6	23,9	23,9	23,9	34,412	0	32,44	2,8E+09	43	Q9HA64	Q9HA64	29,59	29,40	29,40	28,54	28,67	29,07
tr|J3QR09|J3QR	Ribosomal protein	RPL19		0,32	-0,72	4	4	4	22,8	22,8	22,8	23,134	0	29,32	4,5E+08	15	J3QR09;	J3QR09;	26,76	26,35	26,33	24,18	27,33	25,77
sp|Q16527|CSR	Cysteine and glyci	CSRP2	+	2,04	-0,73	5	5	5	35,8	35,8	35,8	20,954	0	33,58	2,8E+09	49	Q16527;	Q16527;	29,70	29,29	29,32	28,60	28,65	28,87
sp|P10599|THIO	Thioredoxin	TXN	+	2,17	-0,74	5	5	5	42,9	42,9	42,9	11,737	0	28,86	3E+09	47	P10599;	P10599;	29,46	29,73	29,60	28,64	29,05	28,88
sp|P62906|RL10	60S ribosomal pro	RPL10A		0,41	-0,75	3	3	3	13,4	13,4	13,4	24,831	0	5,087	2,2E+08	6	P62906	P62906	25,46	25,54	25,79	23,50	26,18	24,86
sp|P84103-2|SR	Serine/arginine-ric	SRSF3	+	1,37	-0,77	4	4	3	30,6	30,6	30,6	14,203	0	21,2	1,6E+09	31	P84103-	P84103-	29,08	28,57	28,21	27,72	27,87	27,97
sp|P50454|SERI	Serpin H1	SERPINH1		0,36	-0,78	3	3	3	11,7	11,7	11,7	46,44	0	16,11	2E+08	11	P50454;	P50454;	25,68	25,50	25,12	25,41	25,67	22,88
sp|P04899|GNA	Guanine nucleotid	GNAI2;GNAS;GNAO1;G		0,54	-0,79	2	2	2	7,3	7,3	7,3	40,45	0	2,967	2,3E+08	10	P04899;	P04899;	25,51	25,88	25,58	25,41	25,60	23,59
tr|E9PPU1|E9PF	40S ribosomal pro	RPS3		0,57	-0,80	9	9	9	63,3	63,3	63,3	17,407	0	21,97	1,3E+09	40	E9PPU1	E9PPU1	28,35	28,13	28,14	26,40	28,54	27,29
tr|A0A590UJK4|	Serine/arginine-ric	SRSF6	+	1,56	-0,80	5	5	3	20,8	20,8	11,5	26,306	0	8,505	5,6E+08	19	A0A590U	A0A590U	27,44	27,17	26,95	26,76	26,21	26,19
tr|E7EX73|E7EX	Eukaryotic translat	EIF4G1		0,64	-0,82	2	2	2	1,7	1,7	1,7	158,64	0	8,418	8,2E+07	4	E7EX73;	E7EX73;	24,24	24,39	24,42	24,12	22,38	24,09
sp|P46781|RS9_	40S ribosomal pro	RPS9		0,61	-0,83	10	10	10	43,8	43,8	43,8	22,591	0	16,33	1,2E+09	36	P46781;	P46781;	28,28	27,90	28,11	26,42	28,43	26,96
sp|P05109|S10A	Protein S100-A8;F	S100A8		0,56	-0,83	3	3	3	26,9	26,9	26,9	10,834	0	9,963	9,9E+08	20	P05109	P05109	27,61	27,34	28,86	26,40	27,98	26,95
tr|A0A494C0J7|	Protein-glutamine	TGM3		1,07	-0,85	6	6	6	8,6	8,6	8,6	78,856	0	11,45	4,8E+08	15	A0A494C	A0A494C	26,36	26,98	27,47	25,77	26,42	26,08
sp|P62277|RS13	40S ribosomal pro	RPS13		1,04	-0,85	6	6	6	35,8	35,8	35,8	17,222	0	25,31	1,2E+09	38	P62277;	P62277;	28,47	28,22	27,96	26,92	28,06	27,12
sp|P26373|RL13	60S ribosomal pro	RPL13		0,26	-0,85	7	7	7	29,9	29,9	29,9	24,261	0	48,13	8,4E+08	25	P26373;	P26373;	27,61	26,80	27,34	24,17	28,51	26,53
sp|P04040|CAT	Catalase	CAT		0,93	-0,86	6	6	6	18	18	18	59,755	0	42,37	6,3E+08	18	P04040	P04040	26,82	27,91	27,13	26,05	26,99	26,24
tr|J3KT73|J3KT7	60S ribosomal pro	RPL38		1,03	-0,87	3	3	3	39,1	39,1	39,1	7,5649	0	39,6	1,1E+09	23	J3KT73;	J3KT73;	28,57	28,22	27,91	26,71	27,47	27,90
sp|P53007|TXTF	Tricarboxylate tran	SLC25A1		0,66	-0,88	2	2	2	7,7	7,7	7,7	34,012	0	11,6	1,3E+08	6	P53007	P53007	25,43	25,41	23,72	23,53	24,16	24,22
sp|P42357-2|HU	Histidine ammonia	HAL		0,84	-0,89	2	2	2	3,4	3,4	3,4	64,887	0	2,736	1E+08	8	P42357-	P42357-	24,42	24,57	25,50	23,26	24,48	24,06
sp|Q9HCY8|S10	Protein S100-A14	S100A14		0,81	-0,91	2	2	2	25	25	25	11,662	0	12,49	1,1E+08	9	Q9HCY8	Q9HCY8	24,83	24,64	24,96	24,37	24,46	22,89
sp|Q9UBV8|PEF	Peflin	PEF1	+	2,04	-0,92	4	4	4	12	12	12	30,381	0	8,4	2,5E+08	13	Q9UBV8	Q9UBV8	26,26	25,91	26,20	25,22	24,92	25,48
sp|Q92688-2|AN	Acidic leucine-rich	ANP32B		0,68	-0,93	5	4	4	28,7	22,1	22,1	22,276	0	10,01	6,8E+08	16	Q92688-	Q92688-	27,85	27,98	26,13	26,74	26,20	26,25
sp|P61313|RL15	60S ribosomal pro	RPL15		0,32	-0,93	6	6	6	32,8	32,8	32,8	24,146	0	30,22	4,7E+08	16	P61313;	P61313;	26,80	26,17	26,62	23,39	27,48	25,93
tr|H7C469|H7C4	Cathepsin D;Cathe	CTSD		1,08	-0,93	4	4	4	12,2	12,2	12,2	35,785	0	21,92	3,9E+08	13	H7C469;	H7C469;	26,18	26,83	27,08	25,68	26,33	25,29
tr|Q5JR95|Q5JR	40S ribosomal pro	RPS8		0,56	-0,95	4	4	4	27,7	27,7	27,7	21,879	0	54,49	1,2E+09	27	Q5JR95;	Q5JR95;	28,11	28,19	28,15	25,90	28,52	27,17
sp|P35998|PRS7	26S protease regu	PSMC2		0,39	-0,96	3	3	3	7,6	7,6	7,6	48,633	0	4,264	2,6E+08	5	P35998;	P35998;	26,11	25,78	25,99	26,16	25,88	22,97
sp|P17096|HMG	High mobility grou	HMGA1	+	1,32	-0,96	2	2	1	22,4	22,4	15	11,676	0	16,07	2E+08	8	P17096;	P17096;	26,28	25,54	25,17	24,81	24,52	24,77
tr|H0YEN5|H0YE	40S ribosomal pro	RPS2		0,69	-0,99	4	4	4	22,6	22,6	22,6	21,154	0	29,08	1,1E+09	23	H0YEN5	H0YEN5	28,21	28,15	27,82	26,10	28,29	26,81
sp|Q96P63|SPB	Serpin B12	SERPINB1	+	1,85	-1,03	6	6	6	17,8	17,8	17,8	46,276	0	35,11	9,8E+08	29	Q96P63;	Q96P63;	27,87	28,33	27,99	26,63	27,26	27,21
tr|J3KRG2|J3KR	Gasdermin-A	GSDMA		0,71	-1,04	2	2	2	13,9	13,9	13,9	17,791	0	4,491	7,1E+07	3	J3KRG2	J3KRG2	23,77	23,79	25,43	22,53	23,72	23,61
tr|M0R210|M0R2	40S ribosomal pro	RPS16		0,90	-1,04	5	5	5	40,3	40,3	40,3	14,419	0	16,88	9,6E+08	33	M0R210	M0R210;	28,28	27,53	27,95	26,09	27,80	26,74
tr|H0YM70|H0YM	Proteasome activa	PSME2		0,41	-1,06	5	5	5	23,2	23,2	23,2	26,011	0	25,41	7,5E+08	8	H0YM70	H0YM70	27,56	26,97	27,41	27,30	24,09	27,36
sp|Q6YN16-2|HS	Hydroxysteroid del	HSDL2		0,57	-1,07	2	2	2	10,1	10,1	10,1	37,32	0	18,41	2,3E+08	13	Q6YN16	Q6YN16	26,40	25,36	25,21	25,40	25,25	23,10
sp|Q58FF8|H90B	Putative heat shoc	HSP90AB2P		0,41	-1,08	6	1	1	16,3	3,9	3,9	44,348	0	3,401	4,9E+08	13	Q58FF8	Q58FF8	27,05	26,36	26,55	26,68	26,68	23,37
sp|P46109|CRKL	Crk-like protein	CRKL	+	2,03	-1,14	7	7	7	33	33	33	33,777	0	60,5	1,3E+09	31	P46109	P46109	28,69	28,64	28,46	27,09	27,40	27,88
tr|X6R700|X6R7	Chromatin target c	CHTOP		1,11	-1,17	2	2	2	11,7	11,7	11,7	23,661	0	18,42	1,5E+08	12	X6R700;	X6R700;	25,47	24,58	24,65	23,36	23,29	24,54
tr|J3KTL2|J3KTL	Serine/arginine-ric	SRSF1	+	2,06	-1,19	7	7	7	26,1	26,1	26,1	28,329	0	20,69	8,9E+08	32	J3KTL2;	J3KTL2;	28,36	27,87	27,52	26,65	26,70	26,83
sp|P47929|LEG7	Galectin-7	LGALS7		1,28	-1,23	4	4	4	35,3	35,3	35,3	15,075	0	12,66	6,8E+08	17	P47929	P47929	26,78	27,71	28,26	26,21	26,61	26,22
sp|Q01650|LAT1	Large neutral amir	SLC7A5		1,10	-1,27	2	2	2	6,3	6,3	6,3	55,01	0	16,79	2,4E+08	17	Q01650	Q01650	26,07	25,98	26,28	25,39	23,76	25,37
tr|Q2L6G8|Q2L6	Corneodesmosin	CDSN		0,89	-1,28	3	3	3	9,6	9,6	9,6	51,539	0	45,11	8,9E+08	16	Q2L6G8	Q2L6G8	27,80	28,81	26,77	26,06	27,12	26,35
tr|H3BV85|H3BV	BolA-like protein 2	BOLA2B;BOLA2		0,89	-1,29	2	2	2	46,3	46,3	46,3	6,3612	0	15,71	1,4E+08	5	H3BV85	H3BV85	25,07	25,61	24,99	22,63	24,49	24,68
sp|P53999|TCP4	Activated RNA pol	SUB1	+	1,64	-1,29	7	7	7	46,5	46,5	46,5	14,395	0	28,62	3,7E+09	34	P53999;	P53999	30,31	30,36	29,34	28,88	28,43	28,82
sp|P14923|PLAK	Junction plakoglob	JUP	+	1,58	-1,31	23	23	23	33,8	33,8	33,8	81,744	0	227,4	1,3E+10	179	P14923;	P14923	31,58	32,39	31,25	30,13	30,72	30,44
sp|Q08554-2|DS	Desmocollin-1	DSC1		1,23	-1,31	10	10	10	16,2	16,2	16,2	93,834	0	136,1	4,1E+09	69	Q08554-	Q08554-	30,05	30,85	29,28	28,43	29,16	28,65
sp|P05089|ARG	Arginase-1	ARG1	+	1,66	-1,32	13	13	13	57,1	57,1	57,1	34,735	0	105,6	2,5E+09	53	P05089;	P05089;	28,97	29,84	29,52	27,66	28,54	28,19
sp|O75340-2|PD	Programmed cell	PDCD6	+	1,38	-1,35	2	2	2	10,1	10,1	10,1	21,664	0	4,348	9,5E+07	6	O75340-	O75340-	25,05	24,79	24,77	23,44	22,78	24,33
tr|H3BV11|H3BV	Nuclear pore comp	NUP93		0,62	-1,37	3	3	3	33,8	33,8	33,8	15,23	0	6,954	1,7E+08	4	H3BV11	H3BV11;	25,26	24,99	25,07	25,72	22,79	22,69
tr|H0YN26|H0YN	Acidic leucine-rich	ANP32A		0,83	-1,39	6	6	5	24,9	24,9	24,9	19,997	0	25,62	1,1E+09	17	H0YN26	H0YN26	28,83	28,87	26,61	27,10	26,35	26,68
sp|P15924|DESI	Desmoplakin	DSP	+	1,46	-1,42	102	102	102	32,6	32,6	32,6	331,77	0	323,3	3,1E+10	553	P15924;	P15924;	32,97	33,74	32,27	31,26	31,78	31,67
sp|P26006|ITA3_	Integrin alpha-3;In	ITGA3	+	1,90	-1,42	2	2	2	1,9	1,9	1,9	116,61	0	4,294	1,3E+08	5	P26006;	P26006;	25,07	24,99	25,30	23,18	24,27	23,65
tr|F8W6P5|F8W	Hemoglobin subur	HBB;HBD	+	2,28	-1,45	4	4	4	64,4	64,4	64,4	9,67	0	11,53	3,6E+08	16	F8W6P5	F8W6P5	26,98	26,89	26,22	25,04	25,40	25,31
sp|Q02413|DSG	Desmoglein-1	DSG1	+	1,33	-1,45	18	18	18	24,9	24,9	24,9	113,75	0	313,4	1E+10	124	Q02413;	Q02413	31,20	32,15	30,66	29,37	30,26	30,02
sp|Q01469|FABI	Fatty acid-binding	FABP5		1,17	-1,48	5	5	5	25,9	25,9	25,9	15,164	0	12,38	2,7E+09	33	Q01469;	Q01469	28,87	30,67	28,89	28,09	27,99	27,92
sp|P04259|K2C6	Keratin, type II cyt	KRT6B		0,95	-1,50	52	4	3	62,4	8,7	5,7	60,066	0	25,12	4E+09	10	P04259	P04259	30,51	30,67	28,88	27,88	28,26	29,44
tr|A0A2R8YFD1	Dynamin-like 120	OPA1		0,97	-1,51	3	3	3	8,8	8,8	8,8	72,406	0	8,039	1,5E+08	6	A0A2R8	A0A2R8	25,88	25,15	24,89	24,54	24,37	22,46
sp|Q14574-2|DS	Desmocollin-3	DSC3	+	1,62	-1,58	5	5	5	10,1	10,1	10,1	93,453	0	105,4	8,3E+08	14	Q14574-	Q14574-	27,83	28,64	27,21	26,03	26,32	26,59
sp|Q6UWP8|SB	Suprabasin	SBSN	+	1,38	-1,59	9	9	9	34,9	34,9	34,9	60,54	0	240,2	1,6E+09	56	Q6UWP	Q6UWP8	28,22	29,81	28,22	26,95	27,25	27,26
sp|P04080|CYTB	Cystatin-B	CSTB		1,19	-1,64	2	2	2	24,5	24,5	24,5	11,139	0	8,507	1,9E+08	7	P04080;	P04080;	25,04	25,17	27,04	24,02	24,14	24,17
sp|Q13835-2|PK	Plakophilin-1	PKP1	+	1,88	-1,66	13	13	13	18,5	18,5	18,5	80,496	0	64,99	1,3E+09	40	Q13835-	Q13835-	28,20	29,18	27,97	26,62	27,01	26,75
tr|F8VYN9|F8VY	ADP-ribosylation fa	ARL1	+	1,48	-1,69	2	2	2	12,9	12,9	12,9	21,778	0	5,286	1,5E+08	6	F8VYN9	F8VYN9	25,53	25,66	25,36	23,56	24,84	23,08
sp|Q8WVV4|PO	Protein POF1B	POF1B	+	1,98	-1,71	6	6	6	12,7	12,7	12,7	68,064	0	28,77	6,6E+08	28	Q8WVV4	Q8WVV4	27,36	28,02	27,58	25,41	26,52	25,92
sp|Q9NZT1|CAL	Calmodulin-like pro	CALML5		0,51	-1,78	6	6	6	47,3	47,3	47,3	15,892	0	293,4	1,5E+09	13	Q9NZT1	Q9NZT1	25,86	30,61	26,11	25,65	25,73	25,85
tr|F8VWU4|F8VW	Ras-related protein	RAB5C;RAB5B;RAB5A		0,64	-1,79	2	2	2	24,2	24,2	24,2	9,9152	0	6,226	3,4E+08	13	F8VWU4	F8VWU4	26,44	26,36	26,73	22,22	25,95	26,01
tr|A0A0B4J1Z1|	Serine/arginine-ric	SRSF7		0,86	-1,80	3	2	2	21,9	21,9	21,9	15,763	0	20,81	4E+08	9	A0A0B4	A0A0B4J	26,95	26,94	26,54	25,70	26,21	23,11
tr|H3BPC4|H3BF	SUMO-conjugating	UBE2I		0,90	-1,85	2	2	2	32,9	32,9	32,9	7,8891	0	4,248	1,7E+08	3	H3BPC4	H3BPC4	25,50	25,41	25,28	22,82	22,37	25,45
tr|M0QZC5|M0Q	40S ribosomal pro	RPS11	+	1,82	-2,00	3	3	3	22	22	22	13,997	0	5,098	2,7E+08	18	M0QZC5	M0QZC5	26,31	26,26	26,01	23,99	25,10	23,47
tr|J3QL05|J3QL0	Serine/arginine-ric	SRSF2;SRSF8		0,74	-2,26	4	4	4	32,3	32,3	32,3	15,156	0	21,73	8,5E+08	20	J3QL05;	J3QL05;	28,30	27,85	27,16	26,92	22,79	26,84
tr|A0A0C4DFV9	Protein SET;Prote	SET;SETSIP		1,00	-2,95	4	4	4	19,5	19,5	19,5	31,124	0	13,91	6,5E+08	18	A0A0C4	A0A0C4	27,69	27,93	27,02	27,17	22,57	24,07

Supplementary Table 2. List of siRNAs

Target	Sequence (5'-3')
BRCA1	AGAUAGUUCUACCAGUAAA
ZNF384-1	GCACAUCCGUAUACACUCA
ZNF384-3	GGCAACACAACAAAGAUAA
DNA-PKcs	CUUUAUGGUGGCCAUGGAG
Ku80	CAAGGAUGAGAUUGCUUUAGU
Luciferase	CGUACGCGGAAUACUUCGA
PARP1	CCAUCGAUGUCAACUAUGA
XRCC4	AUAUGUUGGUGAACUGAGA
PARP3	SMARTpool: ON-TARGETplus (Dharmacon)
Ku70	SMARTpool: siGENOME (Dharmacon)

Supplementary Table 3. List of primers

Name	Sequence (5'-3')
HindIII FW	TAAAAAGCTTATATGGAAGAATCTCACTTCAATTCTAAC
KpnI RV	TAATAATGGTACCCTAAGAGCTGGCCAGGTGC
ZNF384-3_siRNAres_HindII Rv	ACTTGAAGGGCTTGTCCTTATTATGCTGTCGTCT
ZNF384-3_siRNAres_KpnI FW	AGACGACAGCATAATAAGGACAAGCCCTTCAAGT
CMV1 FW	TGGCCCGCCTGGCATTATGCC
CD4int RV	GCTGCCCCAGAATCTTCCTCT
M13 FW	GTAAAACGACGGCCAGT
M13 RV	CAGGAAACAGCTATGAC
ZNF384 △N-terminus(1-209) Fw	CGAGCTCAAGCTTATATGGACCATCAGAAAGAC
ZNF384 △N-terminus(1-209) Rv	GCCGTCTTTCTGATGGTCCATATAAGCTTGAGC
ZNF384 △C2H2(205-410) Fw	ATGCTGGAATCAGGGCTGGCAGTGGCCCAG
ZNF384 △C2H2(205-410) Rv	GGCCTGGGCCACTGCCAGCCCTGATTCGTC
ZNF384 △C-terminus(401-516) Fw	CCGCCTGATCTTCAGCCGTTTAAACCCGCT
ZNF384 △C-terminus(401-516) Rv	AGCGGGTTTAAACGGCTGAAGATCAGGCGG
ZNF384 N-terminus Fw	ATTAAAAAGCTTCTATGGAAGAATCTCACTTCAATTCTAACC C
ZNF384 N-terminus Rv	TAATAATGAATTCTTAGAATTCTTAATCATCATCCTCAGGGG AGAGGAC
ZNF384 C-terminus Fw	ATTAAAAAGCTTCTCAACAGGTGCAGGCAGCAG
ZNF384 C-terminus Rv	TAATAATGAATTCCTAAGAGCTGGCCAGGTGC
ZNF384 C2H2 Fw	ATTAAAAAGCTTCTCCCGAGATGAATGACCCTTATGTCC
ZNF384 C2H2 Rv	TAATAATGAATTCTTATGCCGCTGCTGCTGCTG
pET-His6-MBP-ZNF384 Fw	TACTTCCAATCCAATGCAATGGAAGAATCTCACTTCAATTCT AAC
pET-His6-MBP-ZNF384 Rv	TTATCCACTTCCAATGTTATTACTAAGAGCTGGCCAGGTG
pET-His6-MBP-N-term Fw	ATTAAAAATATTATGGAAGAATCTCACTTCAATTCTAACCC
pET-His6-MBP-N-term Rv	TAATAATGGATVVTTAATCATCATCCTCAGGGGAGAGGAC
pET-His6-MBP-C-term Fw	ATTAAAAATATTCAACAGGTGCAGGCAGCAG
pET-His6-MBP-C-term Rv	TAATAATGGATCCCTAAGAGCTGGCCAGGTGC
pET-His6-MBP-C2H2 Fw	ATTAAAAATATTCCCGAGATGAATGACCCTTATGTCC
pET-His6-MBP-C2H2 Rv	TAATAATGGATCCTTATGCCGCTGCTGCTGCTG
ssDNA T-strand / 3'overhang FW / $dsDNA^{3'-T}$ FW	/5Biosg/CGCTATCGTCTACGTCATGATCGCAGAAAAAGAAAA AAAAAGAA
ssDNA A-strand / $dsDNA^{5'T}$ FW	TTCTTTTTTTTTCTTTTTCTGCGATCATGACGTAGACGATAGC G
forward 5'overhang / $dsDNA^{5'T}$ RV	CGCTATCGTCTACGTCATGATCGC
5'overhang RV	/5Biosg/CGCTATCGTCTACGTCATGATCGC
3'overhang RV	/5Biosg/GCGATCATGACGTAGACGATAGCGTTCTTTTTTTTTC TT TTTCT
$dsDNA^{3'-T}$ RV	AGAAAAAGAAAAAAAAAGAACGCTATCGTCTACGTCATGATC GC
RNA U-strand	/5Biosg/GCGAUCAUGACGUAGACGAUAGCGUUCUUUUUUU UUCUUUUUCU

Supplementary Table 4. List of primary antibodies

Protein	Host	Company	IF	WB
BRCA1	Mouse	Santa Cruz (sc-6954)		1:500
DNA-PKcs	Mouse	Abcam (clone 18-2)		1:750
p-DNA-PKcs (S2056)	Rabbit	Abcam (ab18192)		1:750
GFP	Mouse	Roche (11814460001)		1:2500
Ku80	Rabbit	Santa Cruz (H-300, sc-9034)	1:500	1:1000
PAR	Rabbit	Enzo Lifesciences	1:1000	1:2000
Ku70	Mouse	Santa Cruz (sc-17789)		1:1000
RAD51	Mouse	GeneTex (clone 14B4)	1:200	
α-Tubulin	Mouse	Sigma (cloneDM1A, T6199)		1:5000
XRCC4	Rabbit	Gift from D. van Gent	1:500	
XRCC4	Mouse	SAB (40455)		1:1000
ZNF384	Rabbit	Abcam (ab176689)		1:1000
γH2AX	Mouse	Millipore (clone JBW301, 05-636)	1:2000	1:1000
PARP1	Rabbit	Cell signaling (9542S)		1:1000
GFP	Mouse	Sigma (11814460001)		1:1000
H3	Rabbit	Abcam (ab1791)		1:10000
LIG4	Rabbit	Abcam (ab193353)		1:1000
53BP1	Rabbit	Novus Biologicals (NB100-304)		1:2000
γH2AX	Mouse	Upstate (clone JBW301, #05-636)	1:100	
ZNF384	Rabbit	ATLAS antibodies	1:1000	
PARP3	Rabbit	Gift from F. Dentzer		1:10000
Geminin	Rabbit	Proteintech (10802-1AP)	1:400	
ATM	Rabbit	Cell signaling (clone D2E2)		1:2000
pATM (S1981)	Mouse	Cell signaling (4526)		1:2000
CHK1	Mouse	Santa Cruz (clone G-4, sc8408)		1 :1000
pCHK1 (S345)	Rabbit	Cell signaling (clone 133D3)		1 :1000
RPA32	Mouse	Abcam (ab1275)	1 :1000	
PARP2	Mouse	ENZO lifesciences		1 :1000
Pan-ADPr	Rabbit	Sigma (MABE1016)		1 :1500
Actin	Rabbit	Sigma (A5060)		1 :1000
MBP	Mouse	NEB (E8032S)		1 :10000

Supplementary Table 5. List of biotin-labeled DNA substrates

Name	Sequence
ssDNA T-strand	/5Biosg/-<br>GCGATCATGACGTAGACGATAGCGTTCTTTTTTTTTCTTTTTCT
ssDNA A-strand	TTCTTTTTTTTTCTTTTTCTGCGATCATGACGTAGACGATAGCG-<br>/5Biosg
5’overhang	TTCTTTTTTTTTCTTTTTCTGCGATCATGACGTAGACGATAGCG<br>CGCTAGTACTGCATCTGCTATCGC-<br>/5Biosg/
3’overhang	/5Biosg/-<br>GCGATCATGACGTAGACGATAGCGTTCTTTTTTTTTCTTTTTCT<br>CGCTAGTACTGCATCTGCTATCGC
dsDNA$^{3'-T}$	/5Biosg/-<br>GCGATCATGACGTAGACGATAGCGTTCTTTTTTTTTCTTTTTCT<br><br>CGCTAGTACTGCATCTGCTATCGCAAGAAAAAAAAAGAAAAAGA
dsDNA$^{5'T}$	TTCTTTTTTTTTCTTTTTCTGCGATCATGACGTAGACGATAGCG<br>AAGAAAAAAAAAGAAAAAGACGCTAGTACTGCATCTGCTATCGC-<br>/5Biosg/

Supplementary References

1. Sprague, B.L., et al., *Analysis of binding reactions by fluorescence recovery after photobleaching.* Biophys J, 2004. **86**(6): p. 3473-95.
2. Smith, R., et al., *Poly(ADP-ribose)-dependent chromatin unfolding facilitates the association of DNA-binding proteins with DNA at sites of damage.* Nucleic Acids Res, 2019. **47**(21): p. 11250-11267.
