# Supplementary material for: Zinc finger protein ZNF384 is an adaptor of Ku to DNA during classical non-homologous end-joining
Source: Nat Commun. 2021 Nov 12;12:6560. doi: 10.1038/s41467-021-26691-0 (PMC8589989; doi:10.1038/s41467-021-26691-0)
Supplement: Supplementary file 2 — Reporting Summary [file 41467_2021_26691_MOESM2_ESM.pdf]

nature research

Corresponding author(s): Haico van Attikum

Last updated by author(s): Oct 12, 2021

Reporting Summary

Nature Research wishes to improve the reproducibility of the work that we publish. This form provides structure for consistency and transparency in reporting. For further information on Nature Research policies, see our Editorial Policies and the Editorial Policy Checklist.

Statistics

For all statistical analyses, confirm that the following items are present in the figure legend, table legend, main text, or Methods section.

n/a	Confirmed	
☐	☒	The exact sample size (*n*) for each experimental group/condition, given as a discrete number and unit of measurement
☐	☒	A statement on whether measurements were taken from distinct samples or whether the same sample was measured repeatedly
☐	☒	The statistical test(s) used AND whether they are one- or two-sided<br>*Only common tests should be described solely by name; describe more complex techniques in the Methods section.*
☐	☒	A description of all covariates tested
☒	☐	A description of any assumptions or corrections, such as tests of normality and adjustment for multiple comparisons
☐	☒	A full description of the statistical parameters including central tendency (e.g. means) or other basic estimates (e.g. regression coefficient) AND variation (e.g. standard deviation) or associated estimates of uncertainty (e.g. confidence intervals)
☐	☒	For null hypothesis testing, the test statistic (e.g. *F*, *t*, *r*) with confidence intervals, effect sizes, degrees of freedom and *P* value noted<br>*Give P values as exact values whenever suitable.*
☒	☐	For Bayesian analysis, information on the choice of priors and Markov chain Monte Carlo settings
☒	☐	For hierarchical and complex designs, identification of the appropriate level for tests and full reporting of outcomes
☐	☒	Estimates of effect sizes (e.g. Cohen's *d*, Pearson's *r*), indicating how they were calculated

Our web collection on statistics for biologists contains articles on many of the points above.

Software and code

Policy information about availability of computer code

Data collection

1. Andor IQ software (version 3.6)
2. MaxQuant software suite (version 1.5.5.1; Max Planck Institute of Biochemistry) (mass spectrometry)
3. FACSDiva software version 5.0.3 (flow cytometry)
4. ZEN 2012 (blue edition, version 1.1.0.0) microscopy software (wide-field microscopy)
5. LAS-AF Lite version 1.0.0 (confocal microscopy)
6. Licor Odyssey V3.0 (Western blots)
7. Image Studio Lite version 5.2 (Western blots)
8. Zen Black version 14.0.9.201 (on the LSM880)
9. Metamorph version 7.8.2.0 (on the Nikon Spinning Disk)
10. Huygens Professional (version 19.10) (deconvolution of wide-field 3D images)

Data analysis	1. Image J version 1.48 for microscopy image analysis<br>2. Graphs were plotted and analyzed using GraphPad Prism 8 (version.8.4.2)<br>3. Sanger sequence analyzer for junction analysis (from Schimmel et al., EMBO J., 2017)<br>4. Matlab R2014b (version 8.4.0.150421) routine for quantification of chromatin relaxation and protein recruitment assays<br>5. R version 4.0.5 was used for statistical analysis and the generation of boxplots<br>6. RStudio Version 1.4.1106 was used for statistical analysis and the generation of boxplots<br>7. ForteBio Data Analysis software (version 7.1.0.38) for processing of BLI measurements<br>8. Perseus software (v 1.5.5.3) analysis output for Maxquant<br>9. https://github.com/sehuet/Singh-image-processing for protein recruitment analysis

For manuscripts utilizing custom algorithms or software that are central to the research but not yet described in published literature, software must be made available to editors and reviewers. We strongly encourage code deposition in a community repository (e.g. GitHub). See the Nature Research guidelines for submitting code & software for further information.

Data

Policy information about availability of data

All manuscripts must include a data availability statement. This statement should provide the following information, where applicable:

- Accession codes, unique identifiers, or web links for publicly available datasets
- A list of figures that have associated raw data
- A description of any restrictions on data availability

The mass spectrometry proteomics data generated in this study and shown in Fig. 1D and Table S1 have been deposited to the ProteomeXchange Consortium via the PRIDE partner repository (https://www.ebi.ac.uk/pride/). Access can be obtained with the dataset identifier PDX020417 [73]. Additionally, publicly available reference datasets for Homo sapiens (8th June 2020) was used to search against an in sillico digested UniProt reference proteome. Source data are provided with this paper.

Field-specific reporting

Please select the one below that is the best fit for your research. If you are not sure, read the appropriate sections before making your selection.

☒ Life sciences ☐ Behavioural & social sciences ☐ Ecological, evolutionary & environmental sciences

For a reference copy of the document with all sections, see nature.com/documents/nr-reporting-summary-flat.pdf

Life sciences study design

All studies must disclose on these points even when the disclosure is negative.

Sample size	No statistical method was used to predetermine sample size. Sample sizes for the different experimental approaches were chosen based on technical difficulty, feasibility and the throughput of the individual assays. Moreover, the sample sizes are consistent with previous publications.<br><br>1. Microscopy experiments aimed to acquire a minimum of 100 cells per condition. In some cases, however, when fewer cells were acquired, the precise amount of analyzed cells is mentioned in the Figure legends. The sample size was based on previous publications and common practices in the field (2-3 replicates)<br>2. Reporter assays were performed with acquisition of at least 150.000 cells per condition to obtain a clear GFP-positive cell population which accounted for ~2-5% of the total of cells.<br>3. DSB repair junction analysis was done with ~100 sequences per condition to obtain sufficient wild type and mutated sequences. Sample size was based on a previous publication (Schimmel et al., EMBO J., 2017)<br>4. Survival assays were performed with re-seeding cells in triplicate per condition to average out the variation between technical replicates.
Data exclusions	No data was excluded.
Replication	Experiments were performed at least in duplicate, but the majority in triplicate or more to assess the reproducibility. All attempts at reproduction were successful. Standard errors included in the graph indicate the variation between replicates of each experiment.
Randomization	There was no allocation of test subjects for any of the experiments, thus randomization was not applicable to our study. We work with cell cultures and compare specific experimental conditions or wild type versus knockdown/knockout cell lines, which cannot be randomized.
Blinding	Blinding was not applicable to our study. Still for accuracy, microscopy was performed in an unbiased manner, selecting cells on DAPI signal or expression of proteins of interest without bias on output measures. Data analyses were performed using unbiased software programs/ algorithms. Clonogenic survivals were quantified blinded to prevent subjective counting. Co-immunoprecipitation and in vitro binding assays do not need blinding since all data is obtained and presented in an unbiased manner.

Reporting for specific materials, systems and methods

We require information from authors about some types of materials, experimental systems and methods used in many studies. Here, indicate whether each material, system or method listed is relevant to your study. If you are not sure if a list item applies to your research, read the appropriate section before selecting a response.

Materials & experimental systems

n/a	Involved in the study	
☐	☒	Antibodies
☐	☒	Eukaryotic cell lines
☒	☐	Palaeontology and archaeology
☒	☐	Animals and other organisms
☒	☐	Human research participants
☒	☐	Clinical data
☒	☐	Dual use research of concern

Methods

n/a	Involved in the study	
☒	☐	ChIP-seq
☐	☒	Flow cytometry
☒	☐	MRI-based neuroimaging

Antibodies

Antibodies used

1. anti-BRCA1 (mouse monoclonal, Santa Cruz, clone D9, sc-6954)
2. anti-DNA-PKcs (mouse monoclonal, Abcam, clone 18-2, ab1832)
3. anti-p-DNA-PKcs on Ser2056 (rabbit polyclonal, Abcam, ab18192)
4. anti-Ku70 (mouse monoclonal, Santa Cruz, clone E5, sc-17789)
5. anti-Ku80 (rabbit polyclonal, Santa Cruz, clone H-300, sc-9034)
6. anti-GFP (mouse, clones 7.1 and 13.1, Sigma, #11814460001)
7. anti-α-Tubulin (mouse monoclonal, Sigma, clone DM1A, T6199)
8. anti-PAR (rabbit polyclonal, Trevigen, 4336-BPC-100)
9. anti-RAD51 (mouse, Genetex, clone 14B4, GTX70230)
10. anti-XRCC4 (rabbit, received as a gift from D. van Gent and home made by Modesti et al., EMBO J., 1999)
11. anti-XRCC4 (mouse monoclonal, SAB Signalway, 40455)
12. anti-ZNF384 (rabbit polyclonal, Abcam, ab176689)
13. anti-ZNF384 (rabbit polyclonal, ATLAS antibodies, HPA004051)
14. anti-gH2AX (mouse monoclonal, Millipore, clone JBW301, #05-636)
15. anti-PARP1 (rabbit polyclonal, Cell Signaling, #9542S)
16. anti-Histone H3 (rabbit polyclonal, Abcam, ab1791)
17. anti-LIG4 (rabbit monoclonal, Abcam, ab193353)
18. anti-53BP1 (rabbit polyclonal, NOVUS biologicals NB100-304)
19. anti-PARP3 (rabbit, home made by F. Dentzer, Rodriguez-Vargas et al., Cell Death and Disease, 2020)
20. anti-Geminin (rabbit polyclonal, Proteintech, #10802-1AP)
21. anti-ATM (rabbit monoclonal, Cell Signaling, clone D2E2, #2873)
22. anti-pATM on Ser1981 (mouse monoclonal, Cell signaling, clone 10H11.E12, #4526)
23. anti-CHK1 (mouse monoclonal, Santa Cruz, clone G-4, sc-8408)
24. anti-pCHK1 on Ser345 (rabbit monoclonal, from Cell Signaling, clone 133D3, #2348)
25. anti-RPA32 (mouse monoclonal, Abcam, clone 9H8, ab2175)
26. anti-PARP2 (mouse monoclonal, ENZO, clone 4G8, ALX-804-639)
27. anti-rabbit IgG (CF680, Biotium, #20067, VWR 89138-520)
28. anti-mouse IgG (CF770, Biotium, #20077, VWR 89138-532)
29. anti-PAN-ADP-ribose binding reagent (rabbit, Sigma-Aldrich, MABE1016)
30. anti-MBP (mouse monoclonal, NEB, E8032S)

For anti-XRCC4 (SAB Signalway) and antiMBP antibodies suppliers did not provide information about the clone number.

Validation

The following antibodies were validated by us in knockdown / knockout cells: Rabbit anti-ZNF384 from Abcam (ab176689), Rabbit anti-ZNF384 from ATLAS antibodies (HPA004051), Rabbit anti-Ku80 from Santa Cruz (H-300, sc-9034), Mouse anti-XRCC4 from SAB (40455), Mouse anti-PARP2 by ENZO (clone 4G8, ALX-804-639).

The following antibody was commonly used as a loading control: Mouse anti α-Tubulin from Sigma (cloneDM1A, T6199).

The following antibodies were validated in co-IP experiments:
- anti-GFP (mouse, Sigma, #11814460001) validation stated on suppliers website: https://www.sigmaaldrich.com/deepweb/assets/sigmaaldrich/product/documents/294/951/11814460001bul.pdf
- anti-ZNF384 (rabbit polyclonal, Abcam, ab176689) validation stated on suppliers website: https://www.abcam.com/znf384-antibody-ab176689.html
- anti-MBP (mouse monoclonal, NEB, E8032S) validation stated on suppliers website: https://international.neb.com/products/e8032-anti-mbp-monoclonal-antibody#Product%20Information

The following antibodies were validated by western blot analysis assessing their capacity to detect DNA damage-induced protein phosphorylation:
- anti-ATM (rabbit monoclonal, Cell Signaling, clone D2E2, #2873) validation stated on suppliers website: https://www.cellsignal.com/datasheet.jsp?productId=2873&images=1

- anti-pATM on Ser1981 (mouse monoclonal, Cell signaling, clone 10H11.E12, #4526) validation stated on suppliers website: https://www.cellsignal.com/datasheet.jsp?productId=4526&images=1
- anti-CHK1 (mouse monoclonal, Santa Cruz, clone G-4, sc-8408) validation stated on suppliers website: https://datasheets.scbt.com/sc-8408.pdf
- anti-pCHK1 on Ser345 (rabbit monoclonal, from Cell Signaling, clone 133D3, #2348) validation stated on suppliers website: https://www.cellsignal.com/datasheet.jsp?productId=2348&images=1
- anti-DNA-PKcs (mouse monoclonal, Abcam, clone 18-2, ab1832) validation stated on suppliers website: https://www.citeab.com/antibodies/761874-ab1832-anti-dna-pkcs-antibody-18-2
- anti-p-DNA-PKcs on Ser2056 (rabbit polyclonal, Abcam, ab18192) validation stated on suppliers website: https://www.abcam.com/dna-pkcs-phospho-s2056-antibody-ab18192.html

The following antibodies were validated in IR-induced foci and laser micro-irradiation microscopy experiments:
- anti-PAR (rabbit polyclonal, Trevigen, 4336-BPC-100) validation stated on suppliers website: https://trevigen.com/docs/msds/msds_4336-BPC-100%20Anti-PAR%20Polyclonal.pdf
- anti-XRCC4 (rabbit, received as a gift from D. van Gent and home made and validated by Modesti et al., EMBO J., 1999)
- anti-ZNF384 (rabbit polyclonal, ATLAS antibodies, HPA004051) validation stated on suppliers website: https://www.atlasantibodies.com/api/print_datasheet/HPA004051.pdf

The following antibodies were validated as stated on suppliers website:
- anti-PARP1 (rabbit polyclonal, Cell Signalling Technology #9542) validation stated on suppliers website: https://www.cellsignal.com/datasheet.jsp?productId=9542&images=1&protocol=0
- anti-BRCA1 (mouse monoclonal, Santa Cruz, clone D9, sc-6954) validation stated on suppliers website: https://datasheets.scbt.com/sc-6954.pdf
- anti-Ku70 (mouse monoclonal, Santa Cruz, clone E5, sc-17789) validation stated on suppliers website: https://datasheets.scbt.com/sc-17789.pdf
- anti-RAD51 (mouse, Genetex, clone 14B4, GTX70230) validation stated on suppliers website: https://www.genetex.com/Product/Detail/Rad51-antibody-14B4/GTX70230#datasheet
- anti-gH2AX (mouse monoclonal, Millipore, clone JBW301, #05-636) validation stated on suppliers website: https://www.merckmillipore.com/NL/en/product/msds/MM_NF-05-636?Origin=PDP
- anti-Histone H3 (rabbit polyclonal, Abcam, ab1791) validation stated on suppliers website: https://www.abcam.com/histone-h3-antibody-nuclear-marker-and-chip-grade-ab1791.html
- anti-LIG4 (rabbit monoclonal, Abcam, ab193353) validation stated on suppliers website: https://www.abcam.com/dna-ligase-ivlig4-antibody-epr16531-ab193353.html
- anti-53BP1 (rabbit polyclonal, NOVUS biologicals NB100-304) validation stated on suppliers website: https://www.novusbio.com/products/53bp1-antibody_nb100-304#protocols-faqs
- anti-Geminin (rabbit polyclonal, Proteintech, #10802-1AP) validation stated on suppliers website: https://www.ptglab.com/products/GMNN-Antibody-10802-1-AP.htm
- anti-RPA32 (mouse monoclonal, Abcam, clone 9H8, ab2175) validation stated on suppliers website: https://www.abcam.com/rpa32rpa2-antibody-9h8-ab2175.html
- CF680 goat anti-rabbit IgG (Biotium) validation stated on suppliers website: https://biotium.com/product/goat-anti-rabbit-igg-hlhighly-cross-absorbed/?attribute_pa_conjugation=cf680
- CF770 goat anti-mouse IgG (Biotium) validation stated on suppliers website: https://biotium.com/product/goat-anti-rabbit-igg-hlhighly-cross-absorbed/?attribute_pa_conjugation=cf770
- anti-PAN-ADP-ribose binding reagent (rabbit, Sigma-Aldrich, MABE1016) alidation stated on suppliers website: https://www.merckmillipore.com/NL/en/product/Anti-pan-ADP-ribose-binding-reagent,MM_NF-MABE1016?bd=1#anchor_COA

Eukaryotic cell lines

Policy information about cell lines

Cell line source(s)	Human U2OS cells were purchased from ATCC<br>Several cell lines were previously published:<br>1. VH10-SV40 were a gift from Binie Klein (Klein et. al. Experimental Cell Research, 1990), Sylvius Laboratory, Leiden, NL<br>2. Hela and Hela GFP-Ku80 were a gift from Dik van Gent (Mari et. al. PNAS, 2006), Erasmus Medical Center, Rotterdam, NL<br>4. SV40 T-transformed GM639 human fibroblasts (Taty-Taty et. al. Nucleic Acid Res, 2016), Université de Toulouse, UPS, LBCMCP, F-31062 Toulouse, France<br>5. RPE1-hTERT GFP-Ku70 were a gift from Steve Jackson (Britton et. al. J. Cell. Biol, 2013), Wellcome Trust/Cancer Research Gurdon Institute, Cambridge, UK<br>6. U2OS cells with stably integrated EJ5-GFP reporter were a gift from Jeremy Stark (Pierce et. al. Genes Dev, 1999), City of Hope Comprehensive Cancer Center, Duarte, US<br>7. U2OS cells with stably integrated DR-GFP reporter were a gift from Maria Jasin (Bennardo et. al. Plos genetics, 2008), Memorial Sloan Kettering Cancer Center, New York, USA<br>8. U2OS 2-6-5 cells stably expressing ER-mCherry-LacR-FokI-DD were a gift from Roger Greenberg (Tang et. al. Nat Struct Mol Biol, 2013), Perelman School of Medicine at the University of Pennsylvania, Philadelphia, USA<br>9. 129/Ola-derived IB10 mouse embryonic stem cells WT and Ku80-/- were a gift from Marcel Tijsterman (Schimmel et. al. EMBO J, 2017), Leiden University Medical Center, Leiden, NL<br>10. PARP1, PARP2 and PARP1/2 knockout U2OS cells were a gift from Nicholas Lakin (Ronson et. al. Nature Communications, 2018), University of Oxford, Oxford, UK<br>11. HeLa Flp-In/T-Rex and U2OS Flp-In/T-Rex cells, which were generated using the Flp-In/T-REx system (Thermo Fisher Scientific), were a gift of Geert Kops (University Medical Center Utrecht, NL) and Stephen Taylor (University of Manchester, UK)

12. U2OS-2B2 cells with a stably integrated LacO array for use in the PAR- three-hybrid assay were generated previously (Czarna et al., Cell, 2013), and provided by the Butenandt Institute, Ludwig Maximilians University of Munich, Munich, Germany.
13. U2OS AsiSI–ER- cells were a gift from Gaelle Legube (Iacovoni, et. al. EMBO J, 2010), Centre de Biologie Integrative, Toulouse, FR
14. U2OS cells stably expressing cell cycle marker mKO-Cdt1 were previously generated and published by our laboratory (Luijsterburg, de Krijger et al. 2016), Leiden University Medical Center, Leiden, NL

Authentication	Cell lines were authenticated using Short Tandem Repeat (STR) analysis by ATCC services (100% match).
Mycoplasma contamination	All cell lines were routinely and regularly tested for mycoplasma and used only when non-contaminated.
Commonly misidentified lines (See ICLAC register)	No commonly misidentified cell lines were used in this study.

Flow Cytometry

Plots

Confirm that:

- [x] The axis labels state the marker and fluorochrome used (e.g. CD4-FITC).
- [x] The axis scales are clearly visible. Include numbers along axes only for bottom left plot of group (a 'group' is an analysis of identical markers).
- [x] All plots are contour plots with outliers or pseudocolor plots.
- [x] A numerical value for number of cells or percentage (with statistics) is provided.

Methodology

Sample preparation	2 days after I-Sce-I transfection cells carrying a DSB reporter were trypsinized and diluted in PBS supplemented with 2% FBS
Instrument	BD LSRII flow cytometer (BD Bioscience)
Software	FACSDiva software version 5.0.3. (BD Bioscience)
Cell population abundance	At least 150.000 cells/events were acquired for each condition
Gating strategy	To analyze GFP and mCherry positive U2OS cells carrying the DSB reporters (EJ5-GFP, DR-GFP), three initial gates were set in the following sequential plots: 1) SSC-A scatter (Y-axis) set out against FSC-A scatter (X-axis), allowing us to set gate P1 for living cells, 2) FSC-H scatter (Y-axis) set out against FSC-W scatter (X-axis), allowing us to set gate P2, and 3) SSC-H scatter (Y-axis) set out against SSC-W scatter (X-axis), allowing us to set gate P3. Gates P2 and P3 allowed us to exclude doublets. In a fourth plot (GFP-A on Y-axis and mCherry-A on X-axis), mCherry positive cells were scored using a gating based on mCherry negative control cells (gate P4). Subsequent gate P5 was positioned within mCherry-positive population (within gate P4) to gate for GFP positive cells.<br><br>To analyze GFP positive U2OS cells transfected with pEGFP construct in random plasmid integration assays, three initial gates were set in the following sequential plots: 1) SSC-A scatter (Y-axis) set out against FSC-A scatter (X-axis), allowing us to set gate P1 for living cells, 2) FSC-H scatter (Y-axis) set out against FSC-W scatter (X-axis), allowing us to set gate P2, and 3) SSC-H scatter (Y-axis) set out against SSC-W scatter (X-axis), allowing us to set gate P3. Gates P2 and P3 allowed us to exclude doublets. In a fourth plot (SSC-A on Y-axis and GFP-A on X-axis), GFP positive cells were scored using a gating based on GFP negative control cells (gate P4).

- [x] Tick this box to confirm that a figure exemplifying the gating strategy is provided in the Supplementary Information.
